# Multi-ancestry genome-wide association meta-analysis of hepatocellular carcinoma identifies 15 risk loci including *MAP3K9*, *DHRS1*, *MTTP*, and 8q24.21

**DOI:** 10.1016/j.xhgg.2026.100639

**Published:** 2026-06-25

**Authors:** Ifechukwuamaka Chinaka, Annabelle Schofield, Christopher I. Amos, Lewis R. Roberts, Vincent L. Chen, Younghun Han, Manal Hassan, Shishir Shetty, Jake P. Mann

**Affiliations:** 1 Department of Medicine, St James’ Hospital, Dublin, Ireland; 2 Department of Immunology & Immunotherapy, School of Infection, Inflammation and Immunology, College of Medicine and Health, University of Birmingham, Birmingham, UK; 3 Department of Internal Medicine, University of New Mexico, Albuquerque, NM, USA; 4 Division of Gastroenterology and Hepatology, Mayo Clinic, Rochester, MN, USA; 5 Division of Gastroenterology and Hepatology, Department of Internal Medicine, University of Michigan, Ann Arbor, MI, USA; 6 Department of Epidemiology, The University of Texas MD Anderson Cancer Center, Houston, TX, USA; 7 National Institute for Health and Care Research (NIHR) Birmingham Biomedical Research Centre, University of Birmingham, Birmingham, UK; 8 Liver Unit (including small bowel transplantation), Birmingham Women’s and Children’s NHS Foundation Trust, Birmingham, UK

**Keywords:** liver cancer, cirrhosis, steatosis, genomics

## Abstract

Hepatocellular carcinoma (HCC) is the third leading cause of cancer death globally, often arising on a background of cirrhosis. Here, we aimed to establish genetic drivers of all-cause HCC across ancestries in a large meta-analysis. We included 15 cohorts comprising 17,697 HCC affected individuals and 2,715,683 control subjects in this meta-analysis. We found 15 genome-wide significant (*p* < 5 × 10^−8^) germline loci, including in/near *GCKR*, *MTTP*, *ADH5*, 8q24.21 (nearest gene *MYC*), *MAP3K9*, and *GABPB2*, and a further two loci found on transcriptome- and regulome-wide association analyses. *MAP3K9*, *TERT*, and *GABPB2* variants act independently of cirrhosis on both colocalization analysis and sensitivity analyses. There was significant ancestral heterogeneity in six loci including variants in the HLA locus that had divergent effects on HCC risk between East Asian and European ancestries. Fine mapping identified 11 potentially causal coding variants, including p.Leu446Pro (c.1337T>C) in GCKR and p.Asp423Glu (c.1269C>T) in MEN1. *MEN1*, 8q24.21 (nearest gene *MYC*), and *TERT* are all involved in the β-catenin pathway transactivation complex. Transcriptome-wide analysis identified enrichment of germline-encoded *DHRS1* in HCC. Regulome-wide analysis replicated the germline signal for *EPHA2* and found a chromatin-accessible region containing genes *ZNF367* and *HABP4*. Finally, we demonstrated that population-level genetic architecture for HCC overlaps with steatotic and viral liver disease, and individuals with genetic risk for lower body mass index have higher risk of HCC. Genetic risk for HCC is determined by germline susceptibility to β-catenin pathway activation and cirrhosis. HCC is driven by both heterogeneous and homogeneous genetic factors across ancestries.

## Graphical abstract

**Figure:**
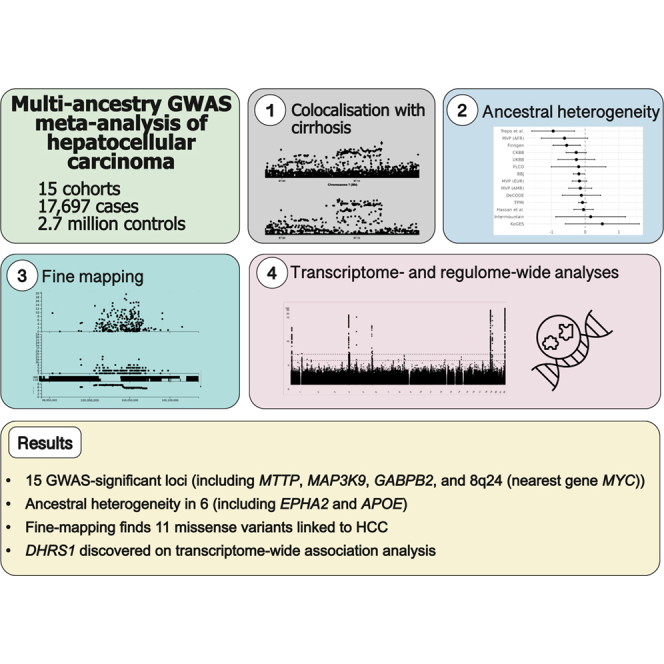

## Teaser

Germline variation contributes to hepatocellular carcinoma risk through susceptibility to β-catenin pathway activation and cirrhosis. This multi-ancestry meta-analysis of 17,697 affected individuals identifies 15 GWAS-significant loci and two further loci through transcriptome- and regulome-wide analyses, including 8q24 (nearest gene *MYC*), *MAP3K9*, and *DHRS1*.

## Introduction

Hepatocellular carcinoma (HCC [MIM: 114550]) is the third most common cause of cancer death globally.^1^ It typically arises on a background of cirrhosis, which can be caused by any etiology of liver disease.^2^ HCC may also develop in non-cirrhotic individuals, particularly in chronic hepatitis B virus (HBV) infection and in metabolic-dysfunction-associated steatotic liver disease (MASLD). Identification of genetic risk factors is critical to mitigating the rapidly growing burden of HCC.^3^

Several previous genome-wide association studies (GWASs) have discovered a total of 35 independent risk loci in individuals of European and East Asian genetic ancestry.^4^^,^^5^^,^^6^^,^^7^ Recent GWASs have demonstrated that combining etiologies of liver disease can increase power to detect variants that perturb common pathways in the mechanisms of liver fibro-inflammation.^8^ For example, an all-cause cirrhosis GWAS identified p.Thr165Pro (c.493A>G) in *MTARC1* (MIM: 614126) as a protective variant, which was not apparent in single-etiology analyses.^9^

Two GWAS meta-analyses of HCC have expanded our understanding of germline variation that drives HCC.^6^^,^^7^ Genes that contribute to HCC can be broadly divided into those that act via cirrhosis and those that are independent of liver fibrosis. Variants in *TM6SF2* (MIM: 606563) and *PNPLA3* (MIM: 609567) are the two most replicated genes that increase risk of cirrhosis liver fibrosis, primarily by increasing hepatic lipid accumulation,^10^ whereas variants in *TERT* (MIM: 187270) appear to directly affect HCC development without any mediation via chronic-liver-disease-related pathways.^7^^,^^11^

Here, we performed a multi-ancestry meta-analysis (MAMA) of HCC GWASs, which included transcriptome-wide and regulome-wide association analyses. In doing so, we identified 15 loci as putative risk genes for HCC and established a baseline for future studies with inclusion of diverse ancestries.

## Material and methods

### Study design and cohort descriptions

We used a single-stage joint meta-analysis design to maximize statistical power: (1) MR-MEGA for heterogeneous effects across ancestries; and (2) sample-size weighted analysis via METAL for homogeneous effects across all ancestries as well as within European (EUR) and East Asian (EAS) ancestries. We used datasets representing the four different ancestry groups EUR, EAS, Admixed American (AMR), and African (AFR), as well as two cohorts of mixed ancestry (which were >70% European, Table S1). GWAS results of Trépo et al.^4^ were acquired through GWAS Catalog (NHGRI: GCST90092003). Summary statistics for Hassan et al.^5^ were previously reported and provided via direct collaboration with the authors. UK Biobank (UKBB), DeCode, and Intermountain summary statistics were obtained from https://www.decode.com/summarydata/ and are reported previously.^12^ The FinnGen GWAS summary statistics were acquired from FinnGen Release 9 (https://www.finngen.fi/en). Taiwan Precision Medicine Initiative (TPMI) summary statistics were acquired from https://pheweb.ibms.sinica.edu.tw/pheno/155.1.^13^^,^^14^ China Kadoorie Biobank (CKBB) summary statistics were acquired from https://pheweb.ckbiobank.org./download/c22.^15^ BioBank Japan (BBJ) summary statistics were acquired from GWAS Catalog (NHGRI: GCST90018638).^16^ Korean BioBank (KoGES) summary statistics were acquired from https://koges.leelabsg.org/.^17^ Prostate, Lung, Colorectal and Ovarian (PLCO) Cancer Screening Trial summary statistics were acquired from https://exploregwas.cancer.gov/plco-atlas.^18^ Millions Veterans Program (MVP) summary statistics were acquired from https://ftp.ncbi.nlm.nih.gov/dbgap/studies/phs002453/analyses/GIA/ as three separate cohorts for EUR, AMR, and AFR, and are reported previously.^19^ All of Us summary statistics were generated through analyses for this study.^20^^,^^21^

GRCh38 were used for analysis and reported throughout. Where GRCh37 was required by specific tools (e.g., FUMA), LiftOver from MungeSumstats^22^ was used for conversion.

### Ethics

This study is a meta-analysis of existing summary-level GWAS data. All primary studies included in this meta-analysis were conducted in accordance with the ethical standards of the responsible relevant institutions, and informed consent was obtained from all participants as described in the original publications. No new data collection or participant recruitment was performed for this study, and therefore no additional ethical approval was required.

### All of Us

Inclusion criteria and genotyping of the All of Us cohort is described in detail elsewhere.^21^ In brief, All of Us is a population-level dataset of approximately 350,000 individuals from the United States. Analysis of the All of Us Research Program dataset was conducted under Research Project ID 65834, in accordance with the All of Us Research Program policies and covered by the All of Us IRB approval. Individuals were included in the current analysis if there was available data on age, sex, and presence or absence of HCC. ICD diagnostic codes (C22.0-ICD03, 155.0-ICD9, and C22.8-ICD10) were used to identify HCC cases. The GWAS was run using Hail^23^ adjusted for age, sex, and the first nine principal components (PCs) of genetic ancestry.

### MAMA

We performed MAMA of GWAS results using MR-MEGA^24^ and METAL.^25^ MR-MEGA accounts for differences in population structure through meta-regression, whereby axes of genetic variation generate covariates for inclusion in the meta-analysis. Four PCs were included in the analysis. MR-MEGA has reduced power to detect associations for variants with homogeneous effects across populations. It is therefore recommended to run MR-MEGA in conjunction with another method, so we used sample-size weighted METAL (as has been reported in two recent meta-analyses^6^^,^^7^) to detect homogeneous allelic effects.

Prior to meta-analysis, all datasets were harmonized to genome build hg38 using MungeSumstats^22^ and R 4.5.0^26^ and included in NCBI GRCh38 (2013-12-17) reference panel. Sex-chromosome data were not available for all cohorts; therefore, only autosomal variants were kept in the results.

To assess potential sample overlap between cohorts of the same ancestry, summary statistics were converted to GRCh37 using MungeSumstats and munged against HapMap3 SNPs using munge_sumstats.py. Pairwise genetic covariance intercepts (gcov_int) were then calculated for all EUR cohort pairs (BCM, deCODE, FinnGen, Intermountain, MVP-EUR, Trépo et al.,^4^ and UKBB) and all EAS cohort pairs (BBJ, CKBB, KoGES, and TPMI) using ldsc.py with EUR and EAS LD score reference panels, respectively. A gcov_int near zero or within the 95% confidence interval (gcov_int ± 1.96 × gcov_int_se) was considered to indicate no meaningful sample overlap (Table S2).

In total, 11,122,532 variants were included in MR-MEGA analyses, as this method has a cohort-number requirement that varies based on the number of axes of variation. Number of variants included in METAL analyses were: 33,182,383 for all cohorts, 22,029,853 for EUR, and 7,735,436 for EAS. Genomic inflations were measured for all cohorts and the meta-analyses. Genomic control was applied to meta-analysis using METAL. All inflation was nominal and below 1.04 for all cohorts and results (highest for MR-MEGA with *λ* = 1.0375 due to increased degrees of freedom in the test (Figure S1). GWAS significance was set at *p* < 5 × 10^−8^ and suggestive threshold set at *p* < 1 × 10^−6^. For variants to be considered GWAS-significant in all ancestry analysis using METAL, they must also reach at least suggestive significance with MR-MEGA. Ancestral (*p*_ANC-HET_) and residual heterogeneity (*p*_RES-HET_) *p* values from MR-MEGA were assessed at all loci reaching suggestive significance (*p* < 1 × 10^−6^, *n* = 76). A Bonferroni-corrected threshold of *p* < 6.6 × 10^−4^ (0.05/76) was applied to declare significant heterogeneity in each analysis.

We identified genomic risk loci within our meta-analysis results using Functional Mapping and Annotation (FUMA) v.1.5.2.^27^^,^^28^ FUMA identifies independent significant SNPs in the GWAS results. FUMA identifies independent significant SNPs by clumping variants at *r*^2^ < 0.1, then defines locus boundaries by merging linkage disequilibrium (LD) blocks of all SNPs in *r*^2^ ≥ 0.6 with any independent significant SNP within 250 kb.

All significant SNPs were compared to the known HCC risk variants reported in previous analyses, including the preprint by Vujkovic et al.^7^ Forest plots and Manhattan plots were generated using R 4.5.0. QQ plots were generated by FUMA.

### Colocalization

To determine whether variants were shared between HCC and cirrhosis, we performed colocalization analysis using summary statistics from the largest available GWAS of cirrhosis.^29^ Summary statistics were standardized using MungeSumstats and converted into variant call format using gwas2vcf for analysis using gwasglue.^30^ Evidence of colocalization due to a common causal variant was defined by PP.H4 ≥0.8.

### Fine mapping

Fine mapping was performed using MR-MEGA, which derives a natural log of Bayes factor (lnBF) per SNP in favor of association, as previously described.^31^ All significant and suggestive SNPs were included for fine mapping. Posterior probabilities (PPs) of driving the association signal at each locus were calculated from the Bayes factor, as previously described.^31^ Credible sets of fewer than five SNPs with sum PP greater than 0.95 were accepted as putative causal variants. Fine-mapped variants were run through Variant Effect Predictor (VEP) to determine functional consequences.^32^

### MAGMA: Gene ontology and tissue enrichment

Results were analyzed using MAGMA^33^ to identify Gene Ontology term enrichment and gene expression data from tissues in GTEx v.8, performed via the GENE2FUNC function of FUMA with default parameters.^28^ Mapped genes were analyzed using gene set analysis for ontology terms from MSigDB v.7 and gene-property analysis for tissue specificity. Results were adjusted for multiple tests using Bonferroni false discovery rate (FDR) correction with alpha of 0.05. The significant ontology terms were filtered for terms that share the same signals. Tissue-level enrichment analysis was carried out using the preprocessed GTEx gene-expression dataset provided by FUMA investigators.

### Transcriptome-wide and regulome-wide association studies

Transcriptome-wide association studies (TWASs) and regulome-wide association studies (RWASs) were performed using FUSION,^34^ which uses tissue-specific (or cancer-specific) expression quantitative trait loci (eQTLs) to determine the effects of germline variation on transcriptome profile (TWAS) or chromatin accessibility (RWAS).^35^ TWAS was performed using (1) GTEx v8 liver precomputed weights and (2) HCC precomputed weights (“LIHC”), as provided by the FUSION investigators, with 1000 Genomes data reference panels. RWAS was performed using known chromatin accessibility profiles for HCC (“LIHC”). All results underwent Benjamini-Hochberg FDR correction with alpha of 0.05.

### LDSC regression analysis across traits

We performed linkage disequilibrium score regression (LDSC) analysis to compare the similarity in genetic architecture across traits. Summary statistics for the largest publicly available GWAS were obtained for cirrhosis (NHGRI: GCST90319878),^29^ liver fat (NHGRI: GCST90016673),^36^ alanine transaminase (ALT; NHGRI: GCST90018943),^16^ hepatitis B viral infection (HBV; NHGRI: GCST90479751),^19^ hepatitis C virus infection (HCV; NHGRI: GCST90018805),^16^ body mass index (BMI; NHGRI: GCST009004),^37^ type 2 diabetes mellitus (T2DM [MIM: 125853]; NHGRI: GCST90444202),^38^ upper gastrointestinal (GI) cancer (gastric and esophageal; NHGRI: GCST90011807),^39^ and colorectal cancer (NHGRI: GCST90129505).^40^ Summary statistics were formatted and converted to GRCh37 using MungeSumstats, then aligned to the 1000 Genomes reference panel. We then ran pairwise regression (--rg) comparisons between all traits.

## Results

### GWAS meta-analyses identify 15 significant loci

**Figure 1:**
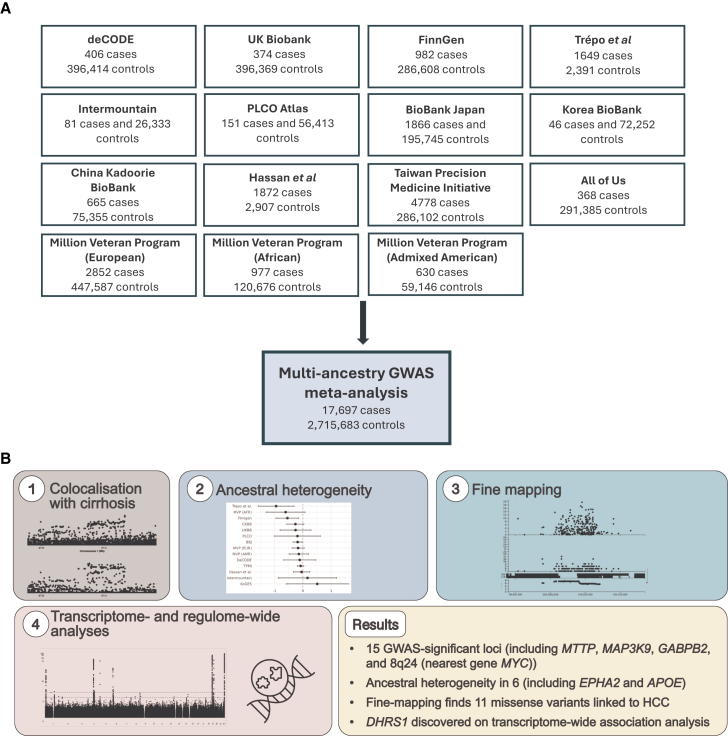
Study overview (A) Fifteen cohorts from four genetic ancestries were included in the meta-analysis, totaling 17,329 hepatocellular carcinoma (HCC) cases. (B) Post-GWAS analysis included colocalization with cirrhosis, multi-marker analysis of genomic annotation (MAGMA) for Gene Ontology and tissue enrichment, fine mapping, and transcriptome-/regulome-wide association studies.

We used four datasets from EAS, seven from EUR, one from AFR, one from AMR, and two from mixed-ancestry cohorts (Figure 1 and Table S1). Overall, this included 17,697 HCC affected individuals and 2,715,683 control subjects in the MAMA. There was no evidence of overlap between cohorts within ancestries based on LDSCs, which were close to zero or within the 95% confidence interval (Table S2).

Following data harmonization and mapping to genome build hg38, MAMA was performed using meta-regression of multi-ethnic genetic association (MR-MEGA) and METAL (including single-ancestry analyses for EUR and EAS cohorts). As previously described, MR-MEGA has greater power to detect heterogeneous effects across different cohorts as well as distinguishing variation in effect estimates from ancestry-level genetic variation. Whereas METAL had better power for identification of homogeneous allelic effects. MR-MEGA also facilitates calculation of posterior probabilities for fine mapping across ancestries using allele frequencies in the different cohorts.

**Figure 2:**
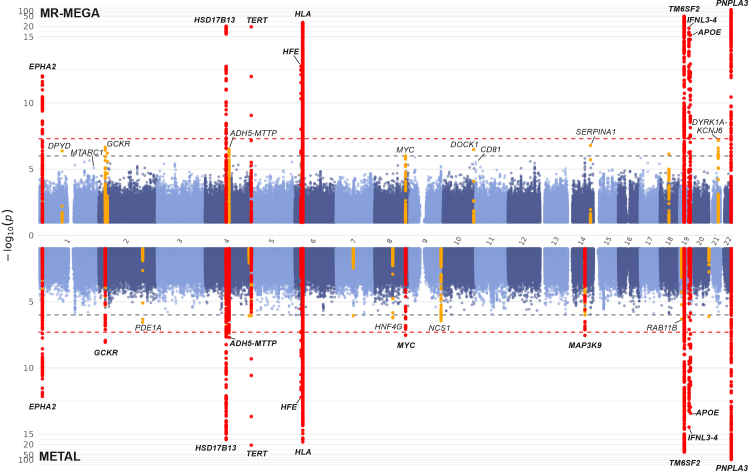
Miami plot of the meta-analysis results from 17,697 HCC affected individuals and 2,715,683 control subjects Results from MR-MEGA (top) and METAL (including all ancestries, bottom), with genome-wide significant (*p* < 5 × 10^−8^) loci in red and top suggestive loci in orange.

In combination, we found 15 loci that met GWAS significance of *p* < 5 × 10^−8^ (Table 1; Figures 2, S1, and S2; Tables S3 and S4). Out of 15 total GWAS-significant loci, nine were observed across both MR-MEGA and METAL models.

**Table 1:** GWAS-significant loci associated with HCC from MR-MEGA and METAL meta-analysis models

rsID	Nearest gene	Genomic position (GRCh38)	Effects	Beta (SE) [MR-MEGA]	*p*(MR-MEGA)	*p*(METAL-all)	*p*(METAL-EUR)	*p*(METAL-EAS)	*p*(ANC-HET)	*p*(RES-HET)	EAF
**Significant**											

rs924204	*EPHA2*	g.16187431A>G (GenBank: NC_000001.11)	+++++-++++-+?+-	0.10 (0.02)	**9**.**20e−13**	**7**.**12e−13**	**1**.**36e−13**	9.47e−3	**5**.**43e−4**	1.23e−1	0.65
rs77018356	*GABPB2*	g.151162485G>C (GenBank: NC_000001.11)	?++++?-+-+-+???	2.90 (3.88)	4.91e−6	1.02e−6	**3**.**84e−8**	2.06e−3	8.97e−2	8.76e−3	0.06
rs780094	*GCKR*	g.27518370T>C (GenBank: NC_000002.12)	-----++-----?-+	−0.08 (0.01)	2.22e−7	**8**.**69e−9**	8.46e−7	9.07e−3	2.22e−1	4.79e−1	0.57
rs4089	*HSD17B13*	g.87300193C>G (GenBank: NC_000004.12)	----+-------?-+	−0.15 (0.02)	**1**.**78e−20**	**2**.**82e−17**	**5**.**74e−18**	4.10e−2	**2**.**01e−9**	1.24e−1	0.3
rs6822348	*ADH5*	g.99132743A>T (GenBank: NC_000004.12)	-----?------???	−0.12 (0.10)	3.12e−7	**4**.**44e−8**	**3**.**43e−8**	3.69e−5	9.90e−1	6.72e−1	0.76
rs112850336	*MTTP*	g.99492512C>T (GenBank: NC_000004.12)	-----+-+---+?--	−0.17 (0.04)	2.57e−6	**2**.**01e−8**	6.83e−5	4.77e−5	5.27e−2	5.65e−1	0.22
rs10069690	*TERT*	g.1279675C>T (GenBank: NC_000005.10)	----++--------+	−0.15 (0.02)	**2**.**50e−20**	**1**.**84e−21**	**6**.**40e−16**	5.77e−5	5.07e−2	8.04e−3	0.25
rs1800562	*HFE*	g.26092913G>A (GenBank: NC_000006.12)	?+++-?+?++-+-??	0.12 (2.89)	**1**.**74e−13**	**5**.**96e−13**	**5**.**58e−14**	9.83e−4	2.26e−2	9.51e−4	0.05
rs1811359	*HLA*	g.33084605G>C (GenBank: NC_000006.12)	+---++----++?++	0.04 (0.01)	**4**.**38e−26**	**8**.**29e−17**	**1**.**18e−8**	**5**.**39e−29**	**1**.**70e−14**	8.76e−1	0.46
rs1561929	*MYC*	g.128555127C>T (GenBank: NC_000008.11)	?++++?++++++???	0.71 (1.70)	1.00e−6	**2**.**96e−8**	8.77e−8	4.28e−4	6.35e−1	5.48e−1	0.88
rs77052733	*MAP3K9*	g.70819179C>T (GenBank: NC_000014.9)	-+++--++++-+?++	0.14 (0.04)	1.54e−6	**2**.**87e−8**	1.22e−6	6.82e−5	2.97e−3	1.55e−1	0.07
rs8107974	*TM6SF2*	g.19277691A>T (GenBank: NC_000019.10)	+++++-++++++?++	0.31 (0.03)	**1**.**95e−48**	**1**.**09e−36**	**2**.**00e−47**	1.63e−2	**5**.**70e−20**	1.74e−2	0.09
rs12983038	*IFNL3*	g.39250484G>A (GenBank: NC_000019.10)	++++--+?++++?+-	0.15 (0.03)	**1**.**91e−17**	**3**.**41e−15**	6.51e−8	5.02e−7	3.83e−3	4.84e−2	0.13
rs429358	*APOE*	g.44908684T>C (GenBank: NC_000019.10)	-----+---------	−0.17 (0.01)	**4**.**93e−16**	**3**.**87e−14**	**1**.**13e−16**	3.16e−2	**9**.**75e−5**	8.42e−1	0.13
rs738408	*PNPLA3*	g.43928850C>T (GenBank: NC_000022.11)	+++++-+++++++++	0.33 (0.02)	**1**.**06e−121**	**2**.**35e−96**	**4**.**41e−96**	**6**.**87e−13**	**1**.**42e−33**	3.18e−2	0.31

**Suggestive**											

rs2642438	*MTARC1*	g.220796686A>G (GenBank: NC_000001.11)	-+++++++++++?+-	0.07 (0.02)	7.01e−5	2.57e−4	5.98e−7	2.05e−2	1.07e−2	2.24e−2	0.77
rs7601754	*STAT4*	g.191075725G>A (GenBank: NC_000002.12)	--+++-++++--?--	−0.01 (0.01)	7.85e−6	5.43e−3	3.11e−4	1.43e−7	**7**.**00e−5**	8.11e−1	0.8
rs59128789	*CD81*	g.2370530T>C (GenBank: NC_000011.10)	++--++?-+--+?+-	0.01 (0.08)	2.97e−6	6.83e−4	3.20e−4	2.08e−3	**1**.**34e−5**	3.34e−1	0.04
rs112635299	*SERPINA1*	g.94371805G>T (GenBank: NC_000014.9)	?+++-?+?+++-???	8.45 (3.89)	1.63e−7	1.11e−6	1.93e−7	3.98e−4	3.64e−2	5.65e−1	0.02

“Effects” indicates the direction of effect on meta-analysis for each study, where “+” represents a positive *β*, “-” a negative *β*, and “?” where this variant was not included in the dataset. Studies included under “Effects” in order (from left to right): Biobank Japan, Hassan et al.,^5^ DeCode, FinnGen, Intermountain, KoGES, PLCO, Trépo et al.,^4^ UKBB, MVP (EUR), MVP (AFR), MVP (AMR), All of Us, TPMI, and CKBB. CHR, chromosome; BP, basepair; NEA, non-effect allele; EA, effect allele. Beta (SE), allelic effect in log odds ratio from MR-MEGA analysis; *p*(MR-MEGA): two-sided *p* value of association from MR-MEGA (chi-squared test with df = 4); *p*(METAL-all), two-sided *p* value of association from sample-size weighted analysis using METAL with all ancestries; *p*(METAL-EUR/EAS), two-sided *p* value of association from sample-size weighted analysis using METAL with European (EUR) or East Asian (EAS) ancestries; *p*(ANC-HET), *p* value for the two-sided ancestral heterogeneity test (chi-squared test with df = 3); *p*(RES-HET), *p* value for the two-sided residual heterogeneity test (chi-squared test with df = 3); EAF, effect allele frequency. Data in bold are significant *p* values (*p* < 5 × 10^−8^ for the two-sided association tests, *p* < 6.6 × 10^−4^ for the heterogeneity tests).

**Figure 3:**
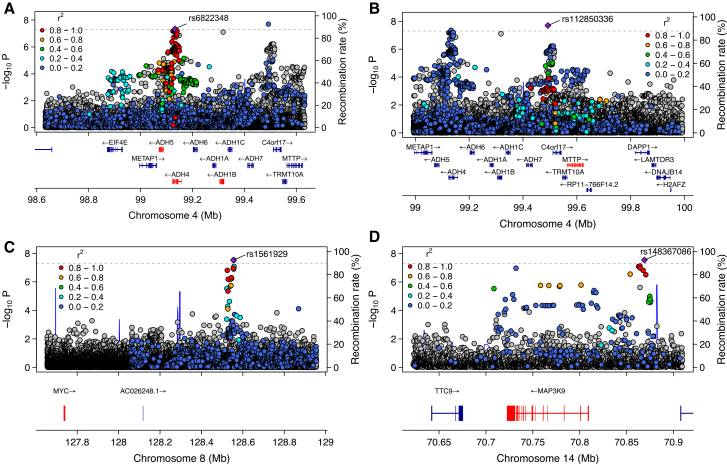
Region plots of genome-wide significant associations LocusZoom plots of genome-wide significant (*p* < 5 × 10^−8^) loci demonstrating individual study effects for *ADH5* (A), *MTTP* (B), 8q24.21 (nearest gene *MYC*) (C), and *MAP3K9* (D).

Three GWAS-significant loci (*GCKR* [MIM: 600842], *ADH-1B* [MIM: 103720]*/-4* [MIM: 103740]*/-5* [MIM: 103710], and *MTTP* [MIM: 157147]) are well-established risk genes for liver disease. *GCKR* (lead SNP rs780094 [g.27518370T>C] [GenBank: NC_000002.12]) is associated with MASLD,^10^ and plasma protein levels of *GCKR* are associated with HCC on Mendelian randomization analyses,^7^ though not with cirrhosis (Table S5). The *ADH-1B/-4/-5* locus (lead SNP rs6822348 [g.99132743A>T] [GenBank: NC_000004.12]) is a genome-wide significant locus for cirrhosis,^29^ confirmed through colocalization analysis (probability of shared causal variant [PP.H4.abf] = 0.90, Table S5), which may be mediated through the effect on alcohol intake (Figure 3A). The causal gene in this region remains unclear as, while typically annotated as *ADH1B* or *ADH4*, rs6822348 has a liver-specific eQTL for *ADH5* (−0.17 normalized effect size, *p* = 8.1 × 10^−8^, from GTEx). It should be noted that Vujkovic et al.^7^ identified a different, coding lead variant in ADH1B, rs1229984 (g.99318162T>A [GenBank: NC_000004.12]) (c.143A>T [GenBank: NM_000668.6] [p.His48Arg]) encoding p.His48Arg to be associated with cirrhosis, which is not in LD with rs6822348 (*R*^2^ < 0.1), suggesting there may be more than one effect in this locus.

*MTTP* (lead SNP rs112850336 [g.99492512C>T] [GenBank: NC_000004.12]) is central to hepatic triglyceride metabolism, which is genome-wide significant for MASLD.^10^ *MTTP* is less than 1 Mb upstream of *ADH5*, but these genes were demonstrated to have independent effects on liver fat in the MASLD GWAS.^10^ Here, we observed separate peaks, and the lead variants (rs6822348 near *ADH5* and rs112850336 near *MTTP*) were not in LD (*R*^2^ = 0.1, Figures 3A and 3B).

SNP rs1561929 (g.128555127C>T [GenBank: NC_000008.11)]) demonstrated consistent effects across all cohorts where present. The nearest protein-coding gene is *MYC* (MIM: 190080) (Figure 3C), located approximately 1 Mb away within the 8q24 locus. *MYC* is amplified or has gain-of-function mutations in 227 out of 373 (61%) HCC individuals based on The Cancer Genome Atlas Program (TCGA) data. *MYC* is a common essential gene (including liver cancer cell lines) in DepMap^41^ and has a probability of having a loss-of-function intolerant (pLI) score of 1.0.^42^

Variants in *MAP3K9* (MIM: 600136) lead SNP rs77052733 (g.70819179C>T [GenBank: NC_000014.9]) were also positively associated with HCC (Figure 3D). 110 of 373 (29.5%) HCC individuals from TCGA have heterozygous deletion of *MAP3K9*.

Lastly, we identified a GWAS-significant locus for EUR ancestry at near *GABPB2* (MIM: 621284) lead SNP rs77018356 (g.151162485G>C [GenBank: NC_000001.11]) (Figures S3A and S3B). There was no evidence of colocalization with variants causing cirrhosis for 8q24.21 (nearest gene *MYC*), *MAP3K9*, *EPHA2* (MIM: 176946), or *GABPB2* loci.

Our analyses also replicated loci in *SERPINA1* (MIM: 107400), *CD81* (MIM: 186845), *STAT4* (MIM: 600558), and *MTARC1* at least suggestive significance (*p* < 1 × 10^−6^), in addition to 57 additional loci (Table S4; Figures S3C and S3D). Prioritization of these loci based on pLI found 13 genes with pLI 0.99–1, including *HNF4G* (MIM: 605966), *MEN1* (MIM: 613733) (Figures S3E and S3F), and *BRINP3*, which may be worthy of further study.

### Cirrhosis-independent effects

Most signals found are in loci previously linked to development of cirrhosis; therefore, we ran a sensitivity analysis in two cohorts with cirrhotic controls (Trépo et al.^4^ and Hassan et al.^5^). We found consistent effects in *APOE* (MIM: 107741), *EPHA2*, *GABPB2*, *HSD17B13* (MIM: 612127), *MAP3K9*, *PNPLA3*, *TERT*, and *TM6SF2*, suggesting that these loci increase risk of HCC even compared to cirrhotic controls (Table S6). There were no variants included in both studies suitable as a proxy for rs1800562 (g.26092913G>A [GenBank: NC_000006.12]) in *HFE* (MIM: 613609). The effects of *ADH5*, *GCKR*, HLA region, *IFNL3* (MIM: 607402), and 8q24.21 (nearest gene *MYC*) were attenuated, suggesting that some of their effect may be via underlying cirrhosis, although there was reduced power in this two-study sensitivity analysis.

### MAMA identifies heterogeneous effects across ancestries

**Figure 4:**
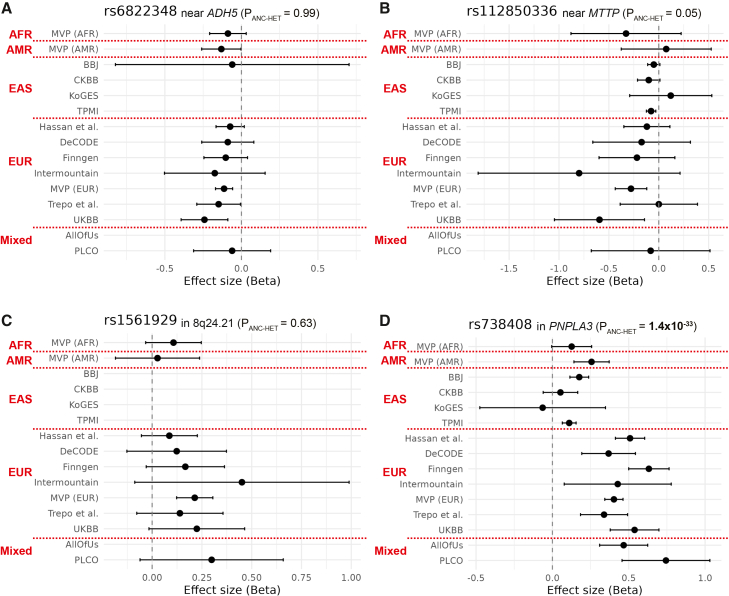
Forest plots of genome-wide significant associations Forest plots of genome-wide significant (*p* < 5 × 10^−8^) loci (top) with forest plots demonstrating individual study effects (below) for *ADH5* (A), *MTTP* (B), rs1561929 (8q24.21 nearest gene *MYC*) (C), and *PNPLA3* (D). rs738408 near *PNPLA3* demonstrated significant ancestral heterogeneity (*p* < 6.6 × 10^−4^). BBJ, BioBank Japan; CKBB, China Kadoorie BioBank; KoGES, Korean BioBank; MVP, Million Veterans Program; PLCO, Prostate, Lung, Colorectal and Ovarian (PLCO) Cancer Screening Trial; TPMI, Taiwan Precision Medicine Initiative; UKBB, UK Biobank. Annotated with genetic ancestry: AFR, African; AMR, Admixed American; EAS, East Asian; EUR, European.

Nine loci showed homogeneous effects across different ancestries (*p*_ANC-HET_ > 6.6 × 10^−4^), including variants in or near *TERT*, *GCKR*, *ADH5*, *MAP3K9*, *MTTP*, and 8q24.21 (Figures 4A–4C and Table 1). For six loci (*PNPLA3*, *TM6SF2*, *EPHA2*, *HSD17B13*, *APOE*, and *HLA*), we found significant ancestral heterogeneity (*p*_ANC-HET_ < 6.6 × 10^−4^) without residual heterogeneity (*p*_RES-HET_ > 6.6 × 10^−4^), suggesting that the results are due to population structural differences instead of other confounders (Figure 4D).

Of note, the *HLA* locus (lead SNP rs1811359; g.33084605G>C [GenBank: NC_000006.12], *p*_ANC-HET_ = 1.7 × 10^−14^) has strongly positive-effect estimates in EAS cohorts while having negative-effect estimates in EUR ancestry cohorts (Figure S3G). The AFR and AMR cohorts demonstrated neutral effects at the *HLA* locus.

Five loci had significant effects in EUR ancestry cohorts and MAMA but no effect in EAS cohorts. The *TM6SF2* locus (lead SNP rs8107974, *p*_ANC-HET_ = 5.7 × 10^−20^) has strongly positive in AMR and EUR ancestry cohorts but no effect in AFR and EAS cohorts (*p*_METAL-EAS_ = 0.016, Figure S3H). Similarly, the *EPHA2*, *APOE*, *HFE*, and *HSD17B13* loci had no significant effects in EAS cohorts (*p* > 1 × 10^−5^) but were directionally consistent with genome-wide significant effects in EUR cohorts. These observations likely reflect the influence of the variants on cirrhosis, as the primary mediator of HCC, in their respective ancestries.

### Fine mapping identifies 11 missense variants

MR-MEGA was used to generate posterior probabilities for fine mapping, which found 28 significant or suggestive loci that had fewer than five variants in the 95% credible set. Within this, we found putative causal coding variants in 11 loci (Tables 2 and S7). This includes the well-established missense mutations in PNPLA3, TM6SF2, HFE, APOE, SERPINA1, and MTARC1. In addition, identified GCKR p.Leu446Pro (c.1337T>C) as the likely causal variant; this missense mutation is strongly associated with insulin resistance and hepatic steatosis.

**Table 2:** Coding variants associated with population risk of HCC identified from fine mapping

chr:start:end	Variant	*p* value	Gene	Protein change	CADD
22:43927717:44014293	g.43928847C>G (GenBank: NC_000022.11) (c.444C>G [GenBank: NM_025225.3] [p.Ile148Met])	1.06e−121 (MR-MEGA)	PNPLA3	p.Ile148Met	13.4
19:18952750:20375946	g.19268740C>T (GenBank: NC_000019.10) (c.499G>A [GenBank: NM_001001524.3] [p.Glu167Lys])	1.95e−48 (MR-MEGA)	TM6SF2	p.Glu167Lys	24.6
19:44883210:44925202	g.44908684T>C (GenBank: NC_000019.10) (c.388T>C [GenBank: NM_000041.4] [p.Cys130Arg])	4.93e−16 (MR-MEGA)	APOE	p.Cys130Arg	16.6
6:25526091:27552168	g.26092913G>A (GenBank: NC_000006.12) (c.845G>A [GenBank: NM_000410.4] [p.Cys282Tyr])	1.74e−13 (MR-MEGA)	HFE	p.Cys282Tyr	27
2:27375230:27529596	g.27508073T>C (GenBank: NC_000002.12) (c.1337T>C [GenBank: NM_001486.4] [p.Leu446Pro])	8.69e−9 (METAL-all)	GCKR	p.Leu446Pro	10.99
10:127419693:127419693	g.127419693G>A (GenBank: NC_000010.11) (c.4657G>A [GenBank: NM_001380.5] [p.Glu1553Lys])	3.35e−7 (MR-MEGA)	DOCK1	p.Glu1553Lys	23.6
19:8359282:8386830	g.8369239G>C (GenBank: NC_000019.10) (c.568G>C [GenBank: NM_139314.3] [p.Glu190Gln])	4.93e−7 (MR-MEGA)	ANGPTL4	p.Glu190Gln	19.01
1:220796686:220800419	g.220796686A>G (GenBank: NC_000001.11) (c.493A>G [GenBank: NM_022746.4] [p.Thr165Pro])	5.98e−7 (METAL-EUR)	MTARC1	p.Thr165Pro	18.3
6:31354452:31359387	g.31355519A>G (GenBank: NC_000006.12) (c.693T>C [GenBank: NM_005514.8] [p.Gly231=])	4.38e−26 (MR-MEGA)	HLA-B	p.Gly231=	–
14:94206394:94378610	g.94378610C>T (GenBank: NC_000014.9) (c.1096G>A [GenBank: NM_000295.5] [p.Glu366Gln])	1.63e−7 (MR-MEGA)	SERPINA1	p.Glu366Gln	24.2
11:64800220:64813774	g.64805130G>A (GenBank: NC_000011.10) (c.1269C>T [GenBank: NM_000244.4] [p.Asp423Glu])	2.25e−6 (MR-MEGA)	MEN1	p.Asp423Glu	19.25

Posterior probabilities were calculated from Bayes scores from MR-MEGA meta-analysis results for all significant and suggestive loci. Loci were filtered for those with fewer than five variants per 95% credible set. Ensembl Variant Effect Predictor was used to identify common coding variants from those credible sets. CADD, combined annotation-dependent depletion score; CHR, chromosome.

Across suggestive loci, we found MEN1 p.Asp423Glu (c.1269C>T) and ANGPTL4 (MIM: 605910) p.Glu190Gln (c.568G>C) missense variants. *MEN1* is a tumor-suppressor gene, where heterozygous loss-of-function mutations cause multiple endocrine neoplasia type 1. *ANGPTL4* is a circulating protein that regulates angiogenesis and inactivation of lipoprotein lipase and therefore influences serum lipid levels.^43^^,^^44^

### *DHRS1* is associated with HCC on transcriptome-wide analysis

To move from statistical association to candidate gene and mechanism, we applied two complementary functional analyses to the meta-analysis results. The GWAS identifies genomic loci associated with HCC risk at a population level but does not itself indicate which genes or regulatory elements mediate that risk. TWAS analysis leverages tissue-specific eQTL data from liver and HCC tissue to test whether germline variation at GWAS loci influences gene expression. RWAS analysis extends this by asking whether germline variants alter chromatin accessibility in HCC tissue, identifying regulatory mechanisms that may mediate risk independently of coding variation. Together, GWAS provides locus discovery, TWAS provides transcriptomic functional annotation, and RWAS provides regulatory annotation: three complementary layers of evidence linking germline variation to HCC risk.

The TWAS using FUSION (Table S9) replicated the above observations for *EPHA2* and *TM6SF2*. In addition, we found a TWAS signal in *DHRS1* (MIM: 610410), lead GWAS-SNP rs2295308 (g.24292599A>C [GenBank: NC_000014.9]) (rs2295308; intergenic/upstream of *DHRS1*, *p*-adj_TWAS_ = 6.3 × 10^−5^) based on MR-MEGA meta-analysis results and HCC-specific eQTL (*p*_eQTL_ = 5.1 × 10^−5^). *DHRS1* is thought to have oxidoreductase activity,^45^ although its function in the liver or malignancy has not yet been characterized.

The RWAS discovered signals for the above GWAS-significant genes *HSD17B13*, *HFE*, *TM6SF2*, and *PNPLA3* (Table S10). In addition, we found a highly significant chromatin accessible region at chr9:96436312–96436813 (lead GWAS-SNP rs16911041 (g.96516265C>A [GenBank: NC_000009.12]) (rs16911041; intergenic), *p*-adj_RWAS_ = 2.7 × 10^−24^). The nearest protein-coding genes are *ZNF367* (MIM: 610160), a transcription factor with tumor-suppressor properties,^46^ and *HABP4* (MIM: 617369), which is implicated in familial colorectal cancer.^47^

### Gene set analysis shows enrichment in liver

We ran MAGMA for Gene Ontology and tissue-level and single-cell expression data.^33^ Enriched Gene Ontology sets in MSigDB v.7.0 were dominated by those related to antigen presentation given mapping to multiple HLA genes (Figure S4A and Table S8). In addition, we observed enrichment of the β-catenin-TCF transactivating complex based on *TERT*, *MEN1*, and histone genes (*p*-adj = 0.03) and GWAS Catalog signatures related to chronic liver disease. Differentially expressed genes were preferentially expressed in liver tissue, based on GTEx data (Figure S4B).

### HCC shares genetic architecture with chronic liver disease but not other gastrointestinal cancers

**Figure 5:**
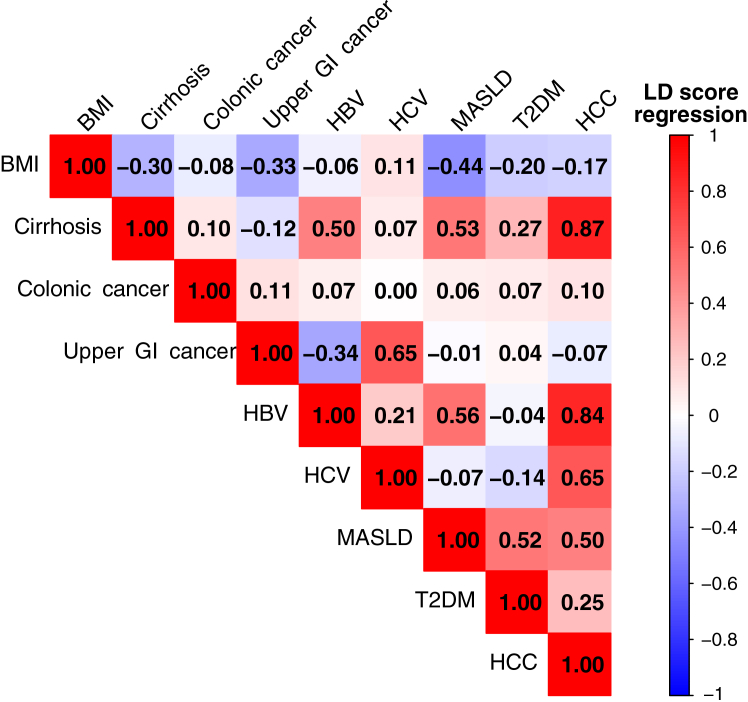
Similarity in genetic architecture between HCC and other gastrointestinal traits LD score regression results between HCC (from this study) and previously published GWASs for chronic liver disease,^16^^,^^19^^,^^29^^,^^36^ metabolic disease (type 2 diabetes mellitus [T2DM],^38^ body mass index [BMI],^37^ and upper gastrointestinal [GI] cancer [esophageal and gastric]),^39^ and colorectal carcinoma.^40^

Finally, we used LD score regression to quantify the similarity in genetic architecture between HCC, liver disorders, and other gastrointestinal cancers (Figure 5). There was a strong positive genomic correlation between HCC and cirrhosis (rg = 0.87, *p* = 5.0 × 10^−10^), HBV (rg = 0.84, *p* = 0.003), HCV (rg = 0.65, *p* = 0.004), and MASLD (rg = 0.50, *p* = 6.0 × 10^−4^), reflecting the underlying drivers of HCC in the population. There was no significant correlation between HCC and upper GI cancer (esophageal and gastric) or colorectal cancer. There were shared genetic determinants between HCC and T2DM (rg = 0.25, *p* = 6.8 × 10^−5^), while there was inverse association between HCC and BMI (rg = −0.17, *p* = 6.0 × 10^−4^), suggesting that genetically lower BMI is linked to increased genetic risk of HCC.

## Discussion

This large GWAS meta-analysis of all-cause HCC has discovered evidence for germline involvement of several genes involved in HCC, most notably 8q24 (nearest gene *MYC*) and *MAP3K9*. We also found genome-wide significant germline associations for HCC in *GCKR*, *ADH5*, and *MTTP*. In addition, we have dissected diverse effects across ancestries and have leveraged these results to fine-map 11 missense variants. Finally, we demonstrate the importance of germline risk for cirrhosis, T2DM, and low BMI as drivers of population risk for HCC.

The 8q24.21 locus has been recognized as a gene desert harboring multiple independent cancer risk loci for prostate,^48^^,^^49^ colorectal,^50^ and breast cancer, none of which align to protein-coding genes.^51^^,^^52^ Functional studies have demonstrated that these loci act as tissue-specific long-range enhancers that physically interact with *MYC* through chromatin looping over several hundred kilobases. The independent signals in different cancer types are not in LD with each other, illustrating that this region harbors multiple tissue-specific regulatory elements. This is of particular relevance in HCC, as *MYC* is a transcription factor that lies downstream of multiple cell-surface signaling pathways, including the WNT-β-catenin pathway,^53^ which is robustly implicated in HCC development.^54^^,^^55^^,^^56^ Variation in 8q24 *MYC* confers increased susceptibility to *WNT* signaling in other malignancies.^57^ We propose that rs1561929 represents an HCC-specific signal at 8q24 operating through an analogous mechanism. It is unclear whether its effect is dependent on cirrhosis or not; there was no evidence of colocalization, but it was not significant on sensitivity analysis using only cirrhotic controls.

The mitogen-activated protein kinase kinase kinases (MAP3K) are a group of proteins that act downstream of cellular stress signals and growth factor receptors. They function as part of a signaling cascade that ultimately leads to pro-growth effects. *MAP3K9* primarily transduces cellular stress through to *JUN* and *GATA4*, which influences mitochondrial cytochrome *c* release and, therefore, apoptosis.^58^^,^^59^ Mutations in *MAP3K9* have been identified in lung cancer and melanoma,^60^^,^^61^ as well as in HCC, via the TCGA dataset. It is not known precisely which stress signals regular *MAP3K9* in the liver, although interactions with a variety of microRNAs have been proposed.^62^^,^^63^

Prevalence of HCC varies greatly across the globe, from 1 in 100,000 in Morocco to 16 in 100,000 in the Republic of Korea.^64^ While this is driven by differences in the prevalence of chronic viral hepatitis, alcohol intake, and obesity, our analysis has uncovered ancestral heterogeneity in germline contribution to HCC risk. Specific HLA loci showed divergent effects in European and East Asian ancestries. Also, in individuals of East Asian ancestry, we observed no significant effect from *TM6SF2*, which was the most significant locus in Europeans.^65^ Some of these differences may reflect the underlying etiology of HCC in these regions (i.e., viral hepatitis versus steatotic liver disease).

Genetically driven accumulation of hepatic lipid droplets has been associated with severity of almost every studied liver disease. In this analysis, we found evidence for six genes (*MTTP*, *PNPLA3*, *TM6SF2*, *HSD17B13*, *GCKR*, and *APOE*) that are all genome-wide risk genes for MASLD.^10^ Gene-environment studies have previously shown that the effect of such variants is amplified by the presence of obesity, T2DM, and excess alcohol consumption.^66^ The stronger effects observed in European cohorts may reflect the higher prevalence of steatotic liver disease in these populations.^67^ It should also be noted that non-cirrhotic HCC is well described in steatotic liver disease.^68^ These data underscore the importance of public health interventions to tackle HCC.

The principal strengths of this study are its size and TWAS/RWAS analyses. The integration of GWAS, TWAS, and RWAS provides three complementary layers of functional evidence. *EPHA2* was identified in both the GWAS and RWAS, providing independent genomic and regulatory support. *DHRS1* emerged specifically through TWAS, demonstrating that germline-encoded transcriptomic variation can identify candidate genes not captured by positional mapping alone. The *ZNF367*/*HABP4* locus at chromosome 9 was identified exclusively through RWAS, representing a chromatin-accessible region that would not have been detected by a GWAS or TWAS. Further mechanistic work is required to understand the function of these loci in HCC development.

This study would be improved by inclusion of individual-participant-level data, which would allow for mediation analyses as performed by Vujkovic et al.^7^ Also, most cohorts used electronic health records for identification of cases, which carries the risk of misclassification. GWAS in the All of Us cohort was performed using Wald logistic regression, which can produce inflated test statistics under case/control imbalance. Although the genomic inflation for this cohort was nominal (*λ* = 1.008) and All of Us contributed no genome-wide significant loci to the meta-analysis, future analyses should consider Firth correction or saddlepoint approximation methods. In addition, we have been unable to robustly identify the causal gene at 8q24.21. We propose that *MYC* is the most likely gene for the rs1561929 variant, but functional work is required to establish this.

Despite the inclusion of multiple ancestries, individuals of European ancestry are still over-represented and account for 49% of HCC cases in this analysis. Individuals of African descent are particularly under-represented, with only 6% of all affected individuals and no data from the continent of Africa, which has some of the highest rates of HCC.^69^ We are also lacking data from the Middle East and South America, where HCC are major causes of cancer death. Addition of data from population biobank studies from these areas will strengthen future MAMA.

Most cohorts included in this analysis had healthy population controls; therefore, this meta-analysis reflects the population-level drivers of HCC, and many loci are already implicated in the development of underlying liver disease (e.g., *TM6SF2*). Our sensitivity analysis with cirrhosis controls and colocalization studies demonstrated that most significant loci, including *MAP3K9*, appear to act independently of cirrhosis. Larger meta-analyses with cirrhotic control individuals or etiology-specific studies may identify further treatment targets.

### Conclusion

HCC is driven by heterogeneous and homogeneous genetic factors across ancestries. Germline susceptibility to β-catenin pathway activation influences HCC risk via *MEN1*, *TERT*, and, potentially, the 8q24 locus (mediated by *MYC*). MAP3K9 and *DHRS1* are HCC risk genes worthy of further investigation. Genes involved in hepatic lipid droplet formation, including *MTTP*, contribute to the risk of HCC. There is a need for further ancestral diversity in liver GWASs.

## Data code and availability

Raw data for Hassan et al.^5^ are available via dbGaP phs001744.v1.p1. All other summary statistics are publicly available via the aforementioned links. Full summary statistics from this analysis can be found on GWAS Catalog (https://www.ebi.ac.uk/gwas/home) using the accession numbers NHGRI: GCST90860789, GCST90860790, GCST90860791, and GCST90860792. All code used in analysis is available at https://github.com/jmann01/HCC-gwas.

## Acknowledgments

J.P.M. is supported by grants from 10.13039/501100024501Birmingham Health Partners, 10.13039/100006662NIHR ACL (CL-2022-09-005), 10.13039/100012361The Royal Society (RG\R1\241312), BSPGHAN-Guts (BSPGHAN2023_01), ESPGHAN, Royal College of Physicians Dame Sheila Sherlock Bursary, and 10.13039/501100022850Little Princess Trust (CCLGA 2024 10 Mann). S.S. is supported by a 10.13039/501100000289Cancer Research UK Advanced Clinician Scientist Fellowship (C53575/A29959). We thank all the participants, investigators, and funders who contributed to the studies included in this meta-analysis. The research was carried out at the 10.13039/501100000272National Institute for Health and Care Research (NIHR) 10.13039/501100018952Birmingham Biomedical Research Centre (BRC).

## Declaration of interests

V.L.C. received grants from AstraZeneca, Takeda, Ipsen, and KOWA, paid to the University of Michigan. S.S. has received honorarium from Astra Zeneca.

## Web resources

China Kadoorie Biobank (CKBB) PheWeb, https://pheweb.ckbiobank.org/download/c22
deCODE/UK Biobank/Intermountain summary statistics, https://www.decode.com/summarydata/
DepMap: The Cancer Dependency Map, https://depmap.org/portal/
FinnGen summary statistics, https://www.finngen.fi/en
GenBank, https://www.ncbi.nlm.nih.gov/genbank/
GWAS Catalog, https://www.ebi.ac.uk/gwas/
gwasglue, https://github.com/MRCIEU/gwasglue
Hail genomic analysis framework, https://github.com/hail-is/hail
HCC GWAS analysis code (this study), https://github.com/jmann01/HCC-gwas
Korean BioBank (KoGES) summary statistics, https://koges.leelabsg.org/
Million Veterans Program (MVP) summary statistics, https://ftp.ncbi.nlm.nih.gov/dbgap/studies/phs002453/analyses/GIA/
Online Mendelian Inheritance in Man (OMIM), https://www.omim.org
PLCO Atlas, https://exploregwas.cancer.gov/plco-atlas
Taiwan Precision Medicine Initiative (TPMI) PheWeb, https://pheweb.ibms.sinica.edu.tw/pheno/155.1

## Supplemental information

Document S1. Figures S1–S4 and captions for Tables S1–S11

Data S1. Tables S1–S11

Document S2. Article plus supplemental information
