# Supplementary material for: Multi-ancestry genome-wide association meta-analysis of hepatocellular carcinoma identifies 15 risk loci including MAP3K9, DHRS1, MTTP, and 8q24.21
Source: HGG Adv. 2026 Jun 25;7(4):100639. doi: 10.1016/j.xhgg.2026.100639 (PMC13445208; doi:10.1016/j.xhgg.2026.100639)
Supplement: Supplementary file 2 [file mmc3.pdf]

HGG Advances

ARTICLE

Multi-ancestry genome-wide association meta-analysis of hepatocellular carcinoma identifies 15 risk loci including *MAP3K9*, *DHRS1*, *MTTP*, and 8q24.21

Ifechukwuamaka Chinaka,[1] Annabelle Schofield,[2] Christopher I. Amos,[3] Lewis R. Roberts,[4] Vincent L. Chen,[5] Younghun Han,[3] Manal Hassan,[6] Shishir Shetty,[7] and Jake P. Mann[2,7,8,9,*]

Summary

Hepatocellular carcinoma (HCC) is the third leading cause of cancer death globally, often arising on a background of cirrhosis. Here, we aimed to establish genetic drivers of all-cause HCC across ancestries in a large meta-analysis. We included 15 cohorts comprising 17,697 HCC affected individuals and 2,715,683 control subjects in this meta-analysis. We found 15 genome-wide significant ($p < 5 \times 10^{-8}$) germline loci, including in/near *GCKR*, *MTTP*, *ADH5*, 8q24.21 (nearest gene *MYC*), *MAP3K9*, and *GABPB2*, and a further two loci found on transcriptome- and regulome-wide association analyses. *MAP3K9*, *TERT*, and *GABPB2* variants act independently of cirrhosis on both colocalization analysis and sensitivity analyses. There was significant ancestral heterogeneity in six loci including variants in the HLA locus that had divergent effects on HCC risk between East Asian and European ancestries. Fine mapping identified 11 potentially causal coding variants, including p.Leu446Pro (c.1337T>C) in GCKR and p.Asp423Glu (c.1269C>T) in MEN1. *MEN1*, 8q24.21 (nearest gene *MYC*), and *TERT* are all involved in the β-catenin pathway transactivation complex. Transcriptome-wide analysis identified enrichment of germline-encoded *DHRS1* in HCC. Regulome-wide analysis replicated the germline signal for *EPHA2* and found a chromatin-accessible region containing genes *ZNF367* and *HABP4*. Finally, we demonstrated that population-level genetic architecture for HCC overlaps with steatotic and viral liver disease, and individuals with genetic risk for lower body mass index have higher risk of HCC. Genetic risk for HCC is determined by germline susceptibility to β-catenin pathway activation and cirrhosis. HCC is driven by both heterogeneous and homogeneous genetic factors across ancestries.

Introduction

Hepatocellular carcinoma (HCC [MIM: 114550]) is the third most common cause of cancer death globally.[1] It typically arises on a background of cirrhosis, which can be caused by any etiology of liver disease.[2] HCC may also develop in non-cirrhotic individuals, particularly in chronic hepatitis B virus (HBV) infection and in metabolic-dysfunction-associated steatotic liver disease (MASLD). Identification of genetic risk factors is critical to mitigating the rapidly growing burden of HCC.[3]

Several previous genome-wide association studies (GWASs) have discovered a total of 35 independent risk loci in individuals of European and East Asian genetic ancestry.[4–7] Recent GWASs have demonstrated that combining etiologies of liver disease can increase power to detect variants that perturb common pathways in the mechanisms of liver fibro-inflammation.[8] For example, an all-cause cirrhosis GWAS identified p.Thr165Pro (c.493A>G) in *MTARC1* (MIM: 614126) as a protective variant, which was not apparent in single-etiology analyses.[9]

Two GWAS meta-analyses of HCC have expanded our understanding of germline variation that drives HCC.[6,7] Genes that contribute to HCC can be broadly divided into those that act via cirrhosis and those that are independent of liver fibrosis. Variants in *TM6SF2* (MIM: 606563) and *PNPLA3* (MIM: 609567) are the two most replicated genes that increase risk of cirrhosis liver fibrosis, primarily by increasing hepatic lipid accumulation,[10] whereas variants in *TERT* (MIM: 187270) appear to directly affect HCC development without any mediation via chronic-liver-disease-related pathways.[7,11]

Here, we performed a multi-ancestry meta-analysis (MAMA) of HCC GWASs, which included transcriptome-wide and regulome-wide association analyses. In doing so, we identified 15 loci as putative risk genes for HCC and established a baseline for future studies with inclusion of diverse ancestries.

[1]Department of Medicine, St James' Hospital, Dublin, Ireland; [2]Department of Immunology & Immunotherapy, School of Infection, Inflammation and Immunology, College of Medicine and Health, University of Birmingham, Birmingham, UK; [3]Department of Internal Medicine, University of New Mexico, Albuquerque, NM, USA; [4]Division of Gastroenterology and Hepatology, Mayo Clinic, Rochester, MN, USA; [5]Division of Gastroenterology and Hepatology, Department of Internal Medicine, University of Michigan, Ann Arbor, MI, USA; [6]Department of Epidemiology, The University of Texas MD Anderson Cancer Center, Houston, TX, USA; [7]National Institute for Health and Care Research (NIHR) Birmingham Biomedical Research Centre, University of Birmingham, Birmingham, UK; [8]Liver Unit (including small bowel transplantation), Birmingham Women's and Children's NHS Foundation Trust, Birmingham, UK
[9]Lead contact
*Correspondence: j.p.mann@bham.ac.uk
https://doi.org/10.1016/j.xhgg.2026.100639.
© 2026 The Author(s). Published by Elsevier Inc. on behalf of American Society of Human Genetics.
This is an open access article under the CC BY license (http://creativecommons.org/licenses/by/4.0/).

Material and methods

Study design and cohort descriptions

We used a single-stage joint meta-analysis design to maximize statistical power: (1) MR-MEGA for heterogeneous effects across ancestries; and (2) sample-size weighted analysis via METAL for homogeneous effects across all ancestries as well as within European (EUR) and East Asian (EAS) ancestries. We used datasets representing the four different ancestry groups EUR, EAS, Admixed American (AMR), and African (AFR), as well as two cohorts of mixed ancestry (which were >70% European, Table S1). GWAS results of Trépo et al.[4] were acquired through GWAS Catalog (NHGRI: GCST90092003). Summary statistics for Hassan et al.[5] were previously reported and provided via direct collaboration with the authors. UK Biobank (UKBB), DeCode, and Intermountain summary statistics were obtained from https://www.decode.com/summarydata/ and are reported previously.[12] The FinnGen GWAS summary statistics were acquired from FinnGen Release 9 (https://www.finngen.fi/en). Taiwan Precision Medicine Initiative (TPMI) summary statistics were acquired from https://pheweb.ibms.sinica.edu.tw/pheno/155.1.[13,14] China Kadoorie Biobank (CKBB) summary statistics were acquired from https://pheweb.ckbiobank.org./download/c22.[15] BioBank Japan (BBJ) summary statistics were acquired from GWAS Catalog (NHGRI: GCST90018638).[16] Korean BioBank (KoGES) summary statistics were acquired from https://koges.leelabsg.org/.[17] Prostate, Lung, Colorectal and Ovarian (PLCO) Cancer Screening Trial summary statistics were acquired from https://exploregwas.cancer.gov/plco-atlas.[18] Millions Veterans Program (MVP) summary statistics were acquired from https://ftp.ncbi.nlm.nih.gov/dbgap/studies/phs002453/analyses/GIA/ as three separate cohorts for EUR, AMR, and AFR, and are reported previously.[19] All of Us summary statistics were generated through analyses for this study.[20,21]

GRCh38 were used for analysis and reported throughout. Where GRCh37 was required by specific tools (e.g., FUMA), LiftOver from MungeSumstats[22] was used for conversion.

Ethics

This study is a meta-analysis of existing summary-level GWAS data. All primary studies included in this meta-analysis were conducted in accordance with the ethical standards of the responsible relevant institutions, and informed consent was obtained from all participants as described in the original publications. No new data collection or participant recruitment was performed for this study, and therefore no additional ethical approval was required.

All of Us

Inclusion criteria and genotyping of the All of Us cohort is described in detail elsewhere.[21] In brief, All of Us is a population-level dataset of approximately 350,000 individuals from the United States. Analysis of the All of Us Research Program dataset was conducted under Research Project ID 65834, in accordance with the All of Us Research Program policies and covered by the All of Us IRB approval. Individuals were included in the current analysis if there was available data on age, sex, and presence or absence of HCC. ICD diagnostic codes (C22.0-ICD03, 155.0-ICD9, and C22.8-ICD10) were used to identify HCC cases. The GWAS was run using Hail[23] adjusted for age, sex, and the first nine principal components (PCs) of genetic ancestry.

MAMA

We performed MAMA of GWAS results using MR-MEGA[24] and METAL.[25] MR-MEGA accounts for differences in population structure through meta-regression, whereby axes of genetic variation generate covariates for inclusion in the meta-analysis. Four PCs were included in the analysis. MR-MEGA has reduced power to detect associations for variants with homogeneous effects across populations. It is therefore recommended to run MR-MEGA in conjunction with another method, so we used sample-size weighted METAL (as has been reported in two recent meta-analyses[6,7]) to detect homogeneous allelic effects.

Prior to meta-analysis, all datasets were harmonized to genome build hg38 using MungeSumstats[22] and R 4.5.0[26] and included in NCBI GRCh38 (2013-12-17) reference panel. Sex-chromosome data were not available for all cohorts; therefore, only autosomal variants were kept in the results.

To assess potential sample overlap between cohorts of the same ancestry, summary statistics were converted to GRCh37 using MungeSumstats and munged against HapMap3 SNPs using munge_sumstats.py. Pairwise genetic covariance intercepts (gcov_int) were then calculated for all EUR cohort pairs (BCM, deCODE, FinnGen, Intermountain, MVP-EUR, Trépo et al.,[4] and UKBB) and all EAS cohort pairs (BBJ, CKBB, KoGES, and TPMI) using ldsc.py with EUR and EAS LD score reference panels, respectively. A gcov_int near zero or within the 95% confidence interval (gcov_int $\pm$ 1.96 $\times$ gcov_int_se) was considered to indicate no meaningful sample overlap (Table S2).

In total, 11,122,532 variants were included in MR-MEGA analyses, as this method has a cohort-number requirement that varies based on the number of axes of variation. Number of variants included in METAL analyses were: 33,182,383 for all cohorts, 22,029,853 for EUR, and 7,735,436 for EAS. Genomic inflations were measured for all cohorts and the meta-analyses. Genomic control was applied to meta-analysis using METAL. All inflation was nominal and below 1.04 for all cohorts and results (highest for MR-MEGA with $\lambda = 1.0375$ due to increased degrees of freedom in the test (Figure S1). GWAS significance was set at $p < 5 \times 10^{-8}$ and suggestive threshold set at $p < 1 \times 10^{-6}$. For variants to be considered GWAS-significant in all ancestry analysis using METAL, they must also reach at least suggestive significance with MR-MEGA. Ancestral ($p_{\text{ANC-HET}}$) and residual heterogeneity ($p_{\text{RES-HET}}$) p values from MR-MEGA were assessed at all loci reaching suggestive significance ($p < 1 \times 10^{-6}$, $n = 76$). A Bonferroni-corrected threshold of $p < 6.6 \times 10^{-4}$ (0.05/76) was applied to declare significant heterogeneity in each analysis.

We identified genomic risk loci within our meta-analysis results using Functional Mapping and Annotation (FUMA) v.1.5.2.[27,28] FUMA identifies independent significant SNPs in the GWAS results. FUMA identifies independent significant SNPs by clumping variants at $r^2 < 0.1$, then defines locus boundaries by merging linkage disequilibrium (LD) blocks of all SNPs in $r^2 \geq 0.6$ with any independent significant SNP within 250 kb.

All significant SNPs were compared to the known HCC risk variants reported in previous analyses, including the preprint by Vujkovic et al.[7] Forest plots and Manhattan plots were generated using R 4.5.0. QQ plots were generated by FUMA.

Colocalization

To determine whether variants were shared between HCC and cirrhosis, we performed colocalization analysis using summary statistics from the largest available GWAS of cirrhosis.[29] Summary

statistics were standardized using MungeSumstats and converted into variant call format using gwas2vcf for analysis using gwasglue.[30] Evidence of colocalization due to a common causal variant was defined by PP.H4 ≥0.8.

Fine mapping

Fine mapping was performed using MR-MEGA, which derives a natural log of Bayes factor (lnBF) per SNP in favor of association, as previously described.[31] All significant and suggestive SNPs were included for fine mapping. Posterior probabilities (PPs) of driving the association signal at each locus were calculated from the Bayes factor, as previously described.[31] Credible sets of fewer than five SNPs with sum PP greater than 0.95 were accepted as putative causal variants. Fine-mapped variants were run through Variant Effect Predictor (VEP) to determine functional consequences.[32]

MAGMA: Gene ontology and tissue enrichment

Results were analyzed using MAGMA[33] to identify Gene Ontology term enrichment and gene expression data from tissues in GTEx v.8, performed via the GENE2FUNC function of FUMA with default parameters.[28] Mapped genes were analyzed using gene set analysis for ontology terms from MSigDB v.7 and gene-property analysis for tissue specificity. Results were adjusted for multiple tests using Bonferroni false discovery rate (FDR) correction with alpha of 0.05. The significant ontology terms were filtered for terms that share the same signals. Tissue-level enrichment analysis was carried out using the preprocessed GTEx gene-expression dataset provided by FUMA investigators.

Transcriptome-wide and regulome-wide association studies

Transcriptome-wide association studies (TWASs) and regulome-wide association studies (RWASs) were performed using FUSION,[34] which uses tissue-specific (or cancer-specific) expression quantitative trait loci (eQTLs) to determine the effects of germline variation on transcriptome profile (TWAS) or chromatin accessibility (RWAS).[35] TWAS was performed using (1) GTEx v8 liver precomputed weights and (2) HCC precomputed weights ("LIHC"), as provided by the FUSION investigators, with 1000 Genomes data reference panels. RWAS was performed using known chromatin accessibility profiles for HCC ("LIHC"). All results underwent Benjamini-Hochberg FDR correction with alpha of 0.05.

LDSC regression analysis across traits

We performed linkage disequilibrium score regression (LDSC) analysis to compare the similarity in genetic architecture across traits. Summary statistics for the largest publicly available GWAS were obtained for cirrhosis (NHGRI: GCST90319878),[29] liver fat (NHGRI: GCST90016673),[36] alanine transaminase (ALT; NHGRI: GCST90018943),[16] hepatitis B viral infection (HBV; NHGRI: GCST90479751),[19] hepatitis C virus infection (HCV; NHGRI: GCST90018805),[16] body mass index (BMI; NHGRI: GCST009004),[37] type 2 diabetes mellitus (T2DM [MIM: 125853]; NHGRI: GCST90444202),[38] upper gastrointestinal (GI) cancer (gastric and esophageal; NHGRI: GCST90011807),[39] and colorectal cancer (NHGRI: GCST90129505).[40] Summary statistics were formatted and converted to GRCh37 using MungeSumstats, then aligned to the 1000 Genomes reference panel. We then ran pairwise regression (–rg) comparisons between all traits.

Results

GWAS meta-analyses identify 15 significant loci

We used four datasets from EAS, seven from EUR, one from AFR, one from AMR, and two from mixed-ancestry cohorts (Figure 1 and Table S1). Overall, this included 17,697 HCC affected individuals and 2,715,683 control subjects in the MAMA. There was no evidence of overlap between cohorts within ancestries based on LDSCs, which were close to zero or within the 95% confidence interval (Table S2).

Following data harmonization and mapping to genome build hg38, MAMA was performed using meta-regression of multi-ethnic genetic association (MR-MEGA) and METAL (including single-ancestry analyses for EUR and EAS cohorts). As previously described, MR-MEGA has greater power to detect heterogeneous effects across different cohorts as well as distinguishing variation in effect estimates from ancestry-level genetic variation. Whereas METAL had better power for identification of homogeneous allelic effects. MR-MEGA also facilitates calculation of posterior probabilities for fine mapping across ancestries using allele frequencies in the different cohorts.

In combination, we found 15 loci that met GWAS significance of $p < 5 \times 10^{-8}$ (Table 1; Figures 2, S1, and S2; Tables S3 and S4). Out of 15 total GWAS-significant loci, nine were observed across both MR-MEGA and METAL models.

Three GWAS-significant loci (*GCKR* [MIM: 600842], *ADH-1B* [MIM: 103720]/*-4* [MIM: 103740]/*-5* [MIM: 103710], and *MTTP* [MIM: 157147]) are well-established risk genes for liver disease. *GCKR* (lead SNP rs780094 [g.27518370T>C] [GenBank: NC_000002.12]) is associated with MASLD,[10] and plasma protein levels of *GCKR* are associated with HCC on Mendelian randomization analyses,[7] though not with cirrhosis (Table S5). The *ADH-1B/-4/-5* locus (lead SNP rs6822348 [g.99132743A>T] [GenBank: NC_000004.12]) is a genome-wide significant locus for cirrhosis,[29] confirmed through colocalization analysis (probability of shared causal variant [PP.H4.abf] = 0.90, Table S5), which may be mediated through the effect on alcohol intake (Figure 3A). The causal gene in this region remains unclear as, while typically annotated as *ADH1B* or *ADH4*, rs6822348 has a liver-specific eQTL for *ADH5* (−0.17 normalized effect size, $p = 8.1 \times 10^{-8}$, from GTEx). It should be noted that Vujkovic et al.[7] identified a different, coding lead variant in ADH1B, rs1229984 (g.99318162T>A [GenBank: NC_000004.12]) (c.143A>T [GenBank: NM_000668.6] [p.His48Arg]) encoding p.His48Arg to be associated with cirrhosis, which is not in LD with rs6822348 ($R^2 < 0.1$), suggesting there may be more than one effect in this locus.

MTTP (lead SNP rs112850336 [g.99492512C>T] [GenBank: NC_000004.12]) is central to hepatic triglyceride metabolism, which is genome-wide significant for MASLD.[10] *MTTP* is less than 1 Mb upstream of *ADH5*,

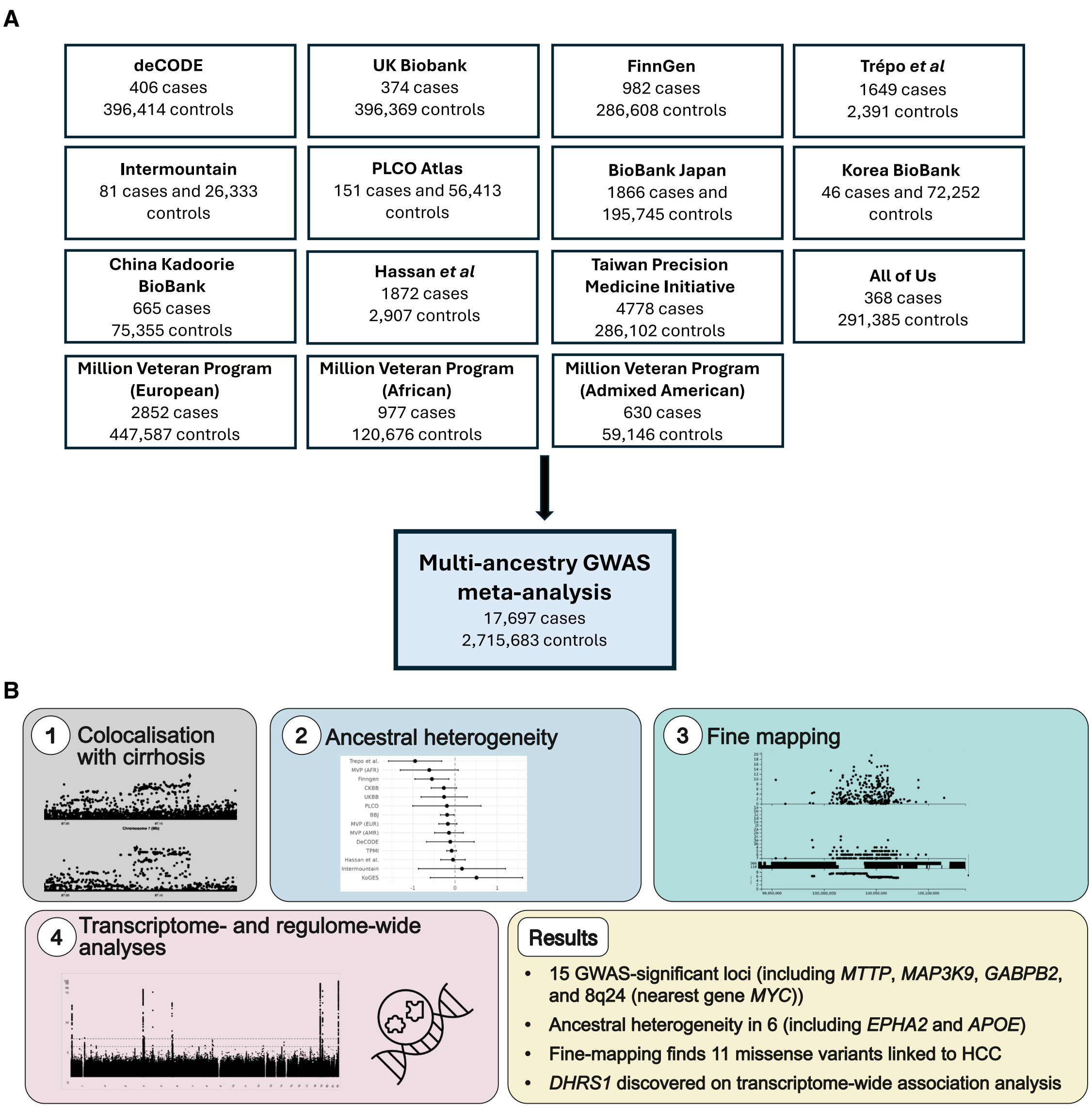

Figure 1. Study overview
(A) Fifteen cohorts from four genetic ancestries were included in the meta-analysis, totaling 17,329 hepatocellular carcinoma (HCC) cases.
(B) Post-GWAS analysis included colocalization with cirrhosis, multi-marker analysis of genomic annotation (MAGMA) for Gene Ontology and tissue enrichment, fine mapping, and transcriptome-/regulome-wide association studies.

but these genes were demonstrated to have independent effects on liver fat in the MASLD GWAS.[10] Here, we observed separate peaks, and the lead variants (rs6822348 near *ADH5* and rs112850336 near *MTTP*) were not in LD ($R^2 = 0.1$, Figures 3A and 3B).

SNP rs1561929 (g.128555127C>T [GenBank: NC_000008.11)]) demonstrated consistent effects across all cohorts where present. The nearest protein-coding gene is *MYC* (MIM: 190080) (Figure 3C), located approximately 1 Mb away within the 8q24 locus. *MYC* is amplified or has gain-of-function mutations in 227 out of 373 (61%) HCC individuals based on The Cancer Genome Atlas Program (TCGA) data. *MYC* is a common essential gene (including liver cancer cell lines) in DepMap[41] and has a probability of having a loss-of-function intolerant (pLI) score of 1.0.[42]

Variants in *MAP3K9* (MIM: 600136) lead SNP rs77052733 (g.70819179C>T [GenBank: NC_000014.9]) were also positively associated with HCC (Figure 3D). 110 of 373 (29.5%) HCC individuals from TCGA have heterozygous deletion of *MAP3K9*.

Lastly, we identified a GWAS-significant locus for EUR ancestry at near *GABPB2* (MIM: 621284) lead SNP rs77018356 (g.151162485G>C [GenBank: NC_000001.11]) (Figures S3A and S3B). There was no evidence of colocalization with variants causing cirrhosis for 8q24.21 (nearest gene *MYC*), *MAP3K9*, *EPHA2* (MIM: 176946), or *GABPB2* loci.

Table 1. GWAS-significant loci associated with HCC from MR-MEGA and METAL meta-analysis models

rsID	Nearest gene	Genomic position (GRCh38)	Effects	Beta (SE) [MR-MEGA]	*p*(MR-MEGA)	*p*(METAL-all)	*p*(METAL-EUR)	*p*(METAL-EAS)	*p*(ANC-HET)	*p*(RES-HET)	EAF
**Significant**											
rs924204	*EPHA2*	g.16187431A>G (GenBank: NC_000001.11)	+++++-+++++--+?+-	0.10 (0.02)	**9.20e−13**	**7.12e−13**	**1.36e−13**	9.47e−3	**5.43e−4**	1.23e−1	0.65
rs77018356	*GABPB2*	g.151162485G>C (GenBank: NC_000001.11)	?++++?-+-+-+???	2.90 (3.88)	4.91e−6	1.02e−6	**3.84e−8**	2.06e−3	8.97e−2	8.76e−3	0.06
rs780094	*GCKR*	g.27518370T>C (GenBank: NC_000002.12)	——++——?-+	−0.08 (0.01)	2.22e−7	**8.69e−9**	8.46e−7	9.07e−3	2.22e−1	4.79e−1	0.57
rs4089	*HSD17B13*	g.87300193C>G (GenBank: NC_000004.12)	——+————?-+	−0.15 (0.02)	**1.78e−20**	**2.82e−17**	**5.74e−18**	4.10e−2	**2.01e−9**	1.24e−1	0.3
rs6822348	*ADH5*	g.99132743A>T (GenBank: NC_000004.12)	——?——???	−0.12 (0.10)	3.12e−7	**4.44e−8**	**3.43e−8**	3.69e−5	9.90e−1	6.72e−1	0.76
rs112850336	*MTTP*	g.99492512C>T (GenBank: NC_000004.12)	——+-+—+?–	−0.17 (0.04)	2.57e−6	**2.01e−8**	6.83e−5	4.77e−5	5.27e−2	5.65e−1	0.22
rs10069690	*TERT*	g.1279675C>T (GenBank: NC_000005.10)	——++————+	−0.15 (0.02)	**2.50e−20**	**1.84e−21**	**6.40e−16**	5.77e−5	5.07e−2	8.04e−3	0.25
rs1800562	*HFE*	g.26092913G>A (GenBank: NC_000006.12)	?+++-?+?++-+-??	0.12 (2.89)	**1.74e−13**	**5.96e−13**	**5.58e−14**	9.83e−4	2.26e−2	9.51e−4	0.05
rs1811359	*HLA*	g.33084605G>C (GenBank: NC_000006.12)	+—++——++?++	0.04 (0.01)	**4.38e−26**	**8.29e−17**	**1.18e−8**	**5.39e−29**	**1.70e−14**	8.76e−1	0.46
rs1561929	*MYC*	g.128555127C>T (GenBank: NC_000008.11)	?++++?++++++???	0.71 (1.70)	1.00e−6	**2.96e−8**	8.77e−8	4.28e−4	6.35e−1	5.48e−1	0.88
rs77052733	*MAP3K9*	g.70819179C>T (GenBank: NC_000014.9)	-+++–+++++-+?++	0.14 (0.04)	1.54e−6	**2.87e−8**	1.22e−6	6.82e−5	2.97e−3	1.55e−1	0.07
rs8107974	*TM6SF2*	g.19277691A>T (GenBank: NC_000019.10)	+++++-+++++++?++	0.31 (0.03)	**1.95e−48**	**1.09e−36**	**2.00e−47**	1.63e−2	**5.70e−20**	1.74e−2	0.09
rs12983038	*IFNL3*	g.39250484G>A (GenBank: NC_000019.10)	++++–+?++++?+-	0.15 (0.03)	**1.91e−17**	**3.41e−15**	6.51e−8	5.02e−7	3.83e−3	4.84e−2	0.13
rs429358	*APOE*	g.44908684T>C (GenBank: NC_000019.10)	——+————	−0.17 (0.01)	**4.93e−16**	**3.87e−14**	**1.13e−16**	3.16e−2	**9.75e−5**	8.42e−1	0.13
rs738408	*PNPLA3*	g.43928850C>T (GenBank: NC_000022.11)	+++++-++++++++++	0.33 (0.02)	**1.06e−121**	**2.35e−96**	**4.41e−96**	**6.87e−13**	**1.42e−33**	3.18e−2	0.31
**Suggestive**											
rs2642438	*MTARC1*	g.220796686A>G (GenBank: NC_000001.11)	-++++++++++++?+-	0.07 (0.02)	7.01e−5	2.57e−4	5.98e−7	2.05e−2	1.07e−2	2.24e−2	0.77
rs7601754	*STAT4*	g.191075725G>A (GenBank: NC_000002.12)	–+++-+++++–?–	−0.01 (0.01)	7.85e−6	5.43e−3	3.11e−4	1.43e−7	**7.00e−5**	8.11e−1	0.8

(*Continued on next page*)

Table 1. *Continued*

rsID	Nearest gene	Genomic position (GRCh38)	Effects	Beta (SE) [MR-MEGA]	*p*(MR-MEGA)	*p*(METAL-all)	*p*(METAL-EUR)	*p*(METAL-EAS)	*p*(ANC-HET)	*p*(RES-HET)	EAF
rs59128789	*CD81*	g.2370530T>C (GenBank: NC_000011.10)	++−++?-+−+?+-	0.01 (0.08)	2.97e−6	6.83e−4	3.20e−4	2.08e−3	**1.34e−5**	3.34e−1	0.04
rs112635299	*SERPINA1*	g.94371805G>T (GenBank: NC_000014.9)	?+++-?+?+++-???	8.45 (3.89)	1.63e−7	1.11e−6	1.93e−7	3.98e−4	3.64e−2	5.65e−1	0.02

"Effects" indicates the direction of effect on meta-analysis for each study, where "+" represents a positive β, "-" a negative β, and "?" where this variant was not included in the dataset. Studies included under "Effects" in order (from left to right): Biobank Japan, Hassan et al.,[5] DeCode, FinnGen, Intermountain, KoGES, PLCO, Trépo et al.,[4] UKBB, MVP (EUR), MVP (AFR), MVP (AMR), All of Us, TPMI, and CKBB. CHR, chromosome; BP, basepair; NEA, non-effect allele; EA, effect allele. Beta (SE), allelic effect in log odds ratio from MR-MEGA analysis; *p*(MR-MEGA): two-sided *p* value of association from MR-MEGA (chi-squared test with df = 4); *p*(METAL-all), two-sided *p* value of association from sample-size weighted analysis using METAL with all ancestries; *p*(METAL-EUR/EAS), two-sided *p* value of association from sample-size weighted analysis using METAL with European (EUR) or East Asian (EAS) ancestries; *p*(ANC-HET), *p* value for the two-sided ancestral heterogeneity test (chi-squared test with df = 3); *p*(RES-HET), *p* value for the two-sided residual heterogeneity test (chi-squared test with df = 3); EAF, effect allele frequency. Data in bold are significant *p* values ($p < 5 \times 10^{-8}$ for the two-sided association tests, $p < 6.6 \times 10^{-4}$ for the heterogeneity tests).

Our analyses also replicated loci in *SERPINA1* (MIM: 107400), *CD81* (MIM: 186845), *STAT4* (MIM: 600558), and *MTARC1* at least suggestive significance ($p < 1 \times 10^{-6}$), in addition to 57 additional loci (Table S4; Figures S3C and S3D). Prioritization of these loci based on pLI found 13 genes with pLI 0.99–1, including *HNF4G* (MIM: 605966), *MEN1* (MIM: 613733) (Figures S3E and S3F), and *BRINP3*, which may be worthy of further study.

Cirrhosis-independent effects

Most signals found are in loci previously linked to development of cirrhosis; therefore, we ran a sensitivity analysis in two cohorts with cirrhotic controls (Trépo et al.[4] and Hassan et al.[5]). We found consistent effects in *APOE* (MIM: 107741), *EPHA2*, *GABPB2*, *HSD17B13* (MIM: 612127), *MAP3K9*, *PNPLA3*, *TERT*, and *TM6SF2*, suggesting that these loci increase risk of HCC even compared to cirrhotic controls (Table S6). There were no variants included in both studies suitable as a proxy for rs1800562 (g.26092913G>A [GenBank: NC_000006.12]) in *HFE* (MIM: 613609). The effects of *ADH5*, *GCKR*, HLA region, *IFNL3* (MIM: 607402), and 8q24.21 (nearest gene *MYC*) were attenuated, suggesting that some of their effect may be via underlying cirrhosis, although there was reduced power in this two-study sensitivity analysis.

MAMA identifies heterogeneous effects across ancestries

Nine loci showed homogeneous effects across different ancestries ($p_{ANC\text{-}HET} > 6.6 \times 10^{-4}$), including variants in or near *TERT*, *GCKR*, *ADH5*, *MAP3K9*, *MTTP*, and 8q24.21 (Figures 4A–4C and Table 1). For six loci (*PNPLA3*, *TM6SF2*, *EPHA2*, *HSD17B13*, *APOE*, and *HLA*), we found significant ancestral heterogeneity ($p_{ANC\text{-}HET} < 6.6 \times 10^{-4}$) without residual heterogeneity ($p_{RES\text{-}HET} > 6.6 \times 10^{-4}$), suggesting that the results are due to population structural differences instead of other confounders (Figure 4D).

Of note, the *HLA* locus (lead SNP rs1811359; g.33084605G>C [GenBank: NC_000006.12], $p_{ANC\text{-}HET} = 1.7 \times 10^{-14}$) has strongly positive-effect estimates in EAS cohorts while having negative-effect estimates in EUR ancestry cohorts (Figure S3G). The AFR and AMR cohorts demonstrated neutral effects at the *HLA* locus.

Five loci had significant effects in EUR ancestry cohorts and MAMA but no effect in EAS cohorts. The *TM6SF2* locus (lead SNP rs8107974, $p_{ANC\text{-}HET} = 5.7 \times 10^{-20}$) has strongly positive in AMR and EUR ancestry cohorts but no effect in AFR and EAS cohorts ($p_{METAL\text{-}EAS} = 0.016$, Figure S3H). Similarly, the *EPHA2*, *APOE*, *HFE*, and *HSD17B13* loci had no significant effects in EAS cohorts ($p > 1 \times 10^{-5}$) but were directionally consistent with genome-wide significant effects in EUR cohorts. These observations likely reflect the influence of the variants on cirrhosis, as the primary mediator of HCC, in their respective ancestries.

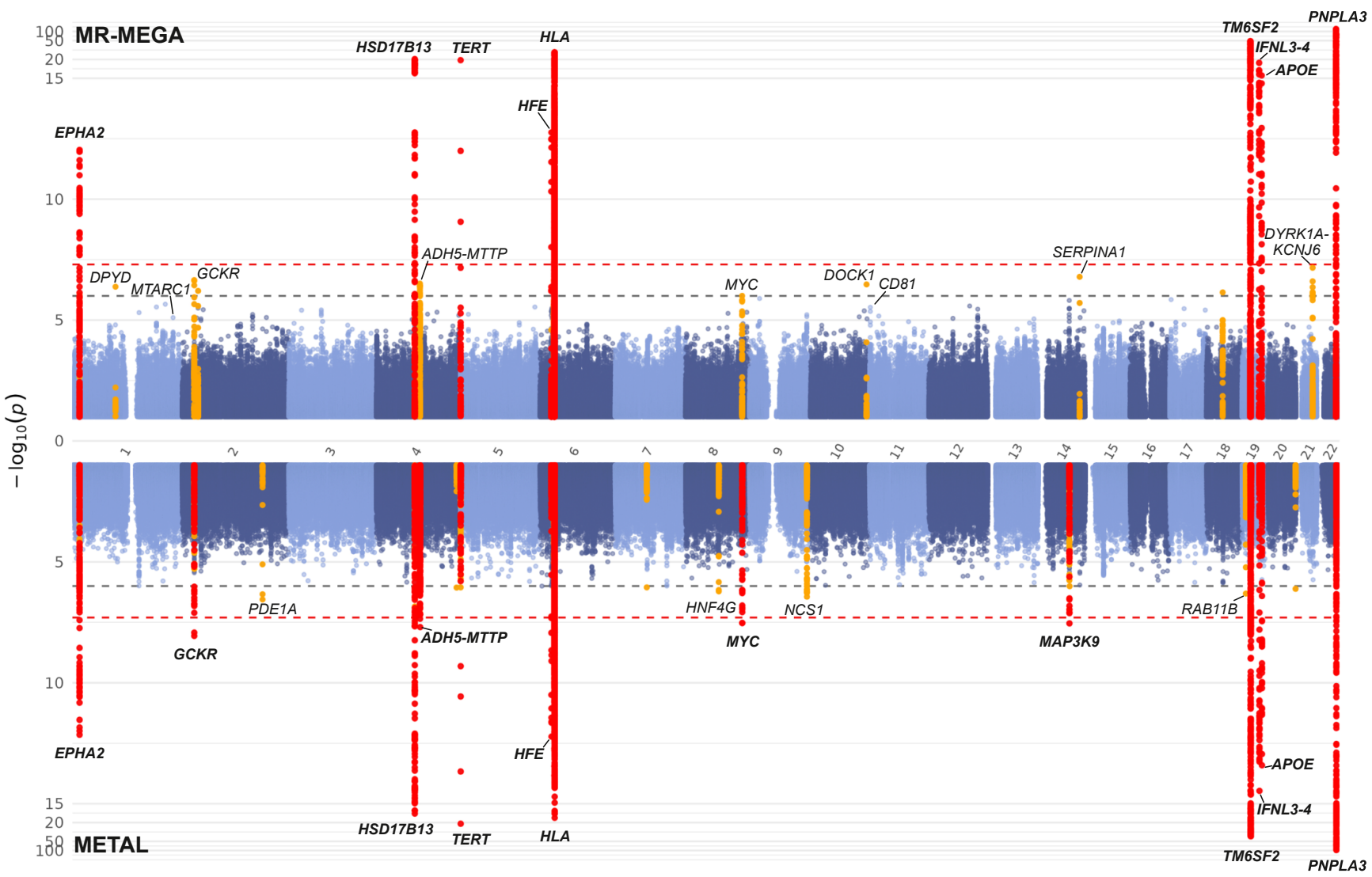

Figure 2. Miami plot of the meta-analysis results from 17,697 HCC affected individuals and 2,715,683 control subjects
Results from MR-MEGA (top) and METAL (including all ancestries, bottom), with genome-wide significant ($p < 5 \times 10^{-8}$) loci in red and top suggestive loci in orange.

Fine mapping identifies 11 missense variants

MR-MEGA was used to generate posterior probabilities for fine mapping, which found 28 significant or suggestive loci that had fewer than five variants in the 95% credible set. Within this, we found putative causal coding variants in 11 loci (Tables 2 and S7). This includes the well-established missense mutations in PNPLA3, TM6SF2, HFE, APOE, SERPINA1, and MTARC1. In addition, identified GCKR p.Leu446Pro (c.1337T>C) as the likely causal variant; this missense mutation is strongly associated with insulin resistance and hepatic steatosis.

Across suggestive loci, we found MEN1 p.Asp423Glu (c.1269C>T) and ANGPTL4 (MIM: 605910) p.Glu190Gln (c.568G>C) missense variants. *MEN1* is a tumor-suppressor gene, where heterozygous loss-of-function mutations cause multiple endocrine neoplasia type 1. *ANGPTL4* is a circulating protein that regulates angiogenesis and inactivation of lipoprotein lipase and therefore influences serum lipid levels.[43,44]

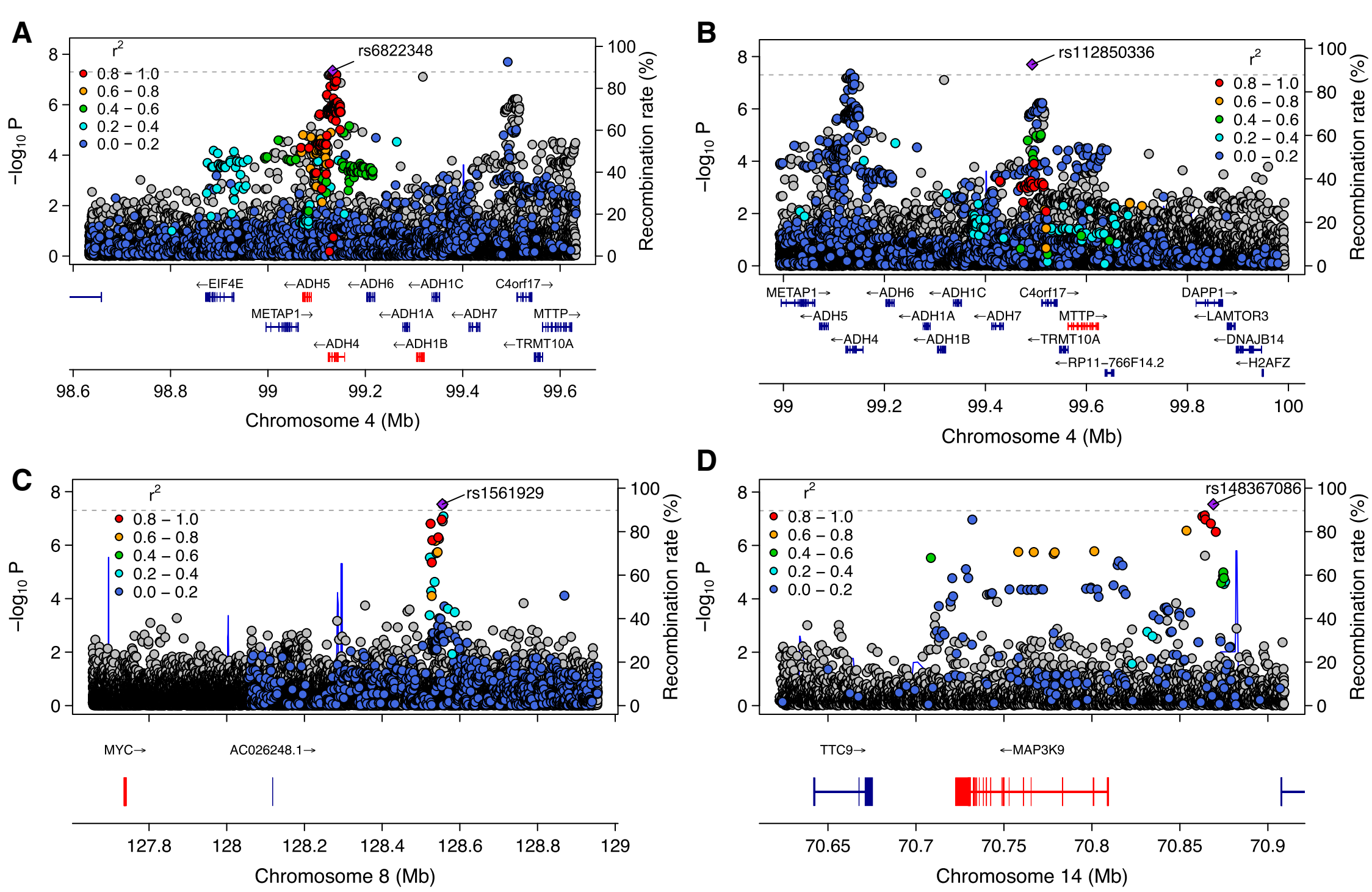

Figure 3. Region plots of genome-wide significant associations
LocusZoom plots of genome-wide significant ($p < 5 \times 10^{-8}$) loci demonstrating individual study effects for *ADH5* (A), *MTTP* (B), 8q24.21 (nearest gene *MYC*) (C), and *MAP3K9* (D).

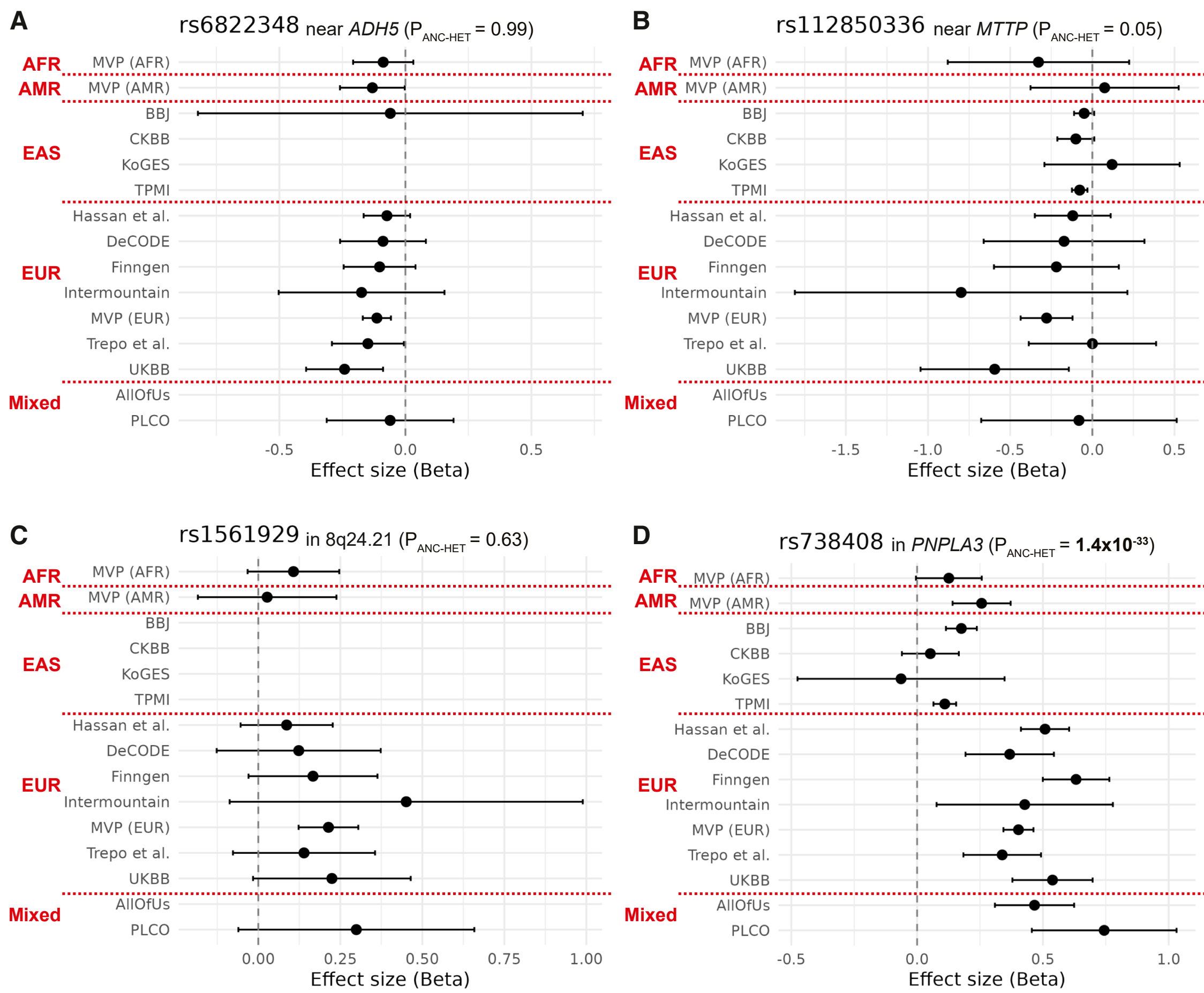

Figure 4. Forest plots of genome-wide significant associations
Forest plots of genome-wide significant ($p < 5 \times 10^{-8}$) loci (top) with forest plots demonstrating individual study effects (below) for *ADH5* (A), *MTTP* (B), rs1561929 (8q24.21 nearest gene *MYC*) (C), and *PNPLA3* (D). rs738408 near *PNPLA3* demonstrated significant ancestral heterogeneity ($p < 6.6 \times 10^{-4}$). BBJ, BioBank Japan; CKBB, China Kadoorie BioBank; KoGES, Korean BioBank; MVP, Million Veterans Program; PLCO, Prostate, Lung, Colorectal and Ovarian (PLCO) Cancer Screening Trial; TPMI, Taiwan Precision Medicine Initiative; UKBB, UK Biobank. Annotated with genetic ancestry: AFR, African; AMR, Admixed American; EAS, East Asian; EUR, European.

DHRS1 is associated with HCC on transcriptome-wide analysis

To move from statistical association to candidate gene and mechanism, we applied two complementary functional analyses to the meta-analysis results. The GWAS identifies genomic loci associated with HCC risk at a population level but does not itself indicate which genes or regulatory elements mediate that risk. TWAS analysis leverages tissue-specific eQTL data from liver and HCC tissue to test whether germline variation at GWAS loci influences gene expression. RWAS analysis extends this by asking whether germline variants alter chromatin accessibility in HCC tissue, identifying regulatory mechanisms that may mediate risk independently of coding variation. Together, GWAS provides locus discovery, TWAS provides transcriptomic functional annotation, and RWAS provides regulatory annotation: three complementary layers of evidence linking germline variation to HCC risk.

The TWAS using FUSION (Table S9) replicated the above observations for *EPHA2* and *TM6SF2*. In addition, we found a TWAS signal in *DHRS1* (MIM: 610410), lead GWAS-SNP rs2295308 (g.24292599A>C [GenBank: NC_000014.9]) (rs2295308; intergenic/upstream of *DHRS1*, $p\text{-adj}_{\text{TWAS}} = 6.3 \times 10^{-5}$) based on MR-MEGA meta-analysis results and HCC-specific eQTL ($p_{\text{eQTL}} = 5.1 \times 10^{-5}$). *DHRS1* is thought to have oxidoreductase activity,[45] although its function in the liver or malignancy has not yet been characterized.

The RWAS discovered signals for the above GWAS-significant genes *HSD17B13*, *HFE*, *TM6SF2*, and *PNPLA3* (Table S10). In addition, we found a highly significant chromatin accessible region at chr9:96436312–96436813 (lead GWAS-SNP rs16911041 (g.96516265C>A [GenBank: NC_000009.12]) (rs16911041; intergenic), $p\text{-adj}_{\text{RWAS}} = 2.7 \times 10^{-24}$). The nearest protein-coding genes are *ZNF367* (MIM: 610160), a transcription factor with

Table 2. Coding variants associated with population risk of HCC identified from fine mapping

chr:start:end	Variant	*p* value	Gene	Protein change	CADD
22:43927717:44014293	g.43928847C>G (GenBank: NC_000022.11) (c.444C>G [GenBank: NM_025225.3] [p.Ile148Met])	1.06e−121 (MR-MEGA)	PNPLA3	p.Ile148Met	13.4
19:18952750:20375946	g.19268740C>T (GenBank: NC_000019.10) (c.499G>A [GenBank: NM_001001524.3] [p.Glu167Lys])	1.95e−48 (MR-MEGA)	TM6SF2	p.Glu167Lys	24.6
19:44883210:44925202	g.44908684T>C (GenBank: NC_000019.10) (c.388T>C [GenBank: NM_000041.4] [p.Cys130Arg])	4.93e−16 (MR-MEGA)	APOE	p.Cys130Arg	16.6
6:25526091:27552168	g.26092913G>A (GenBank: NC_000006.12) (c.845G>A [GenBank: NM_000410.4] [p.Cys282Tyr])	1.74e−13 (MR-MEGA)	HFE	p.Cys282Tyr	27
2:27375230:27529596	g.27508073T>C (GenBank: NC_000002.12) (c.1337T>C [GenBank: NM_001486.4] [p.Leu446Pro])	8.69e−9 (METAL-all)	GCKR	p.Leu446Pro	10.99
10:127419693:127419693	g.127419693G>A (GenBank: NC_000010.11) (c.4657G>A [GenBank: NM_001380.5] [p.Glu1553Lys])	3.35e−7 (MR-MEGA)	DOCK1	p.Glu1553Lys	23.6
19:8359282:8386830	g.8369239G>C (GenBank: NC_000019.10) (c.568G>C [GenBank: NM_139314.3] [p.Glu190Gln])	4.93e−7 (MR-MEGA)	ANGPTL4	p.Glu190Gln	19.01
1:220796686:220800419	g.220796686A>G (GenBank: NC_000001.11) (c.493A>G [GenBank: NM_022746.4] [p.Thr165Pro])	5.98e−7 (METAL-EUR)	MTARC1	p.Thr165Pro	18.3
6:31354452:31359387	g.31355519A>G (GenBank: NC_000006.12) (c.693T>C [GenBank: NM_005514.8] [p.Gly231=])	4.38e−26 (MR-MEGA)	HLA-B	p.Gly231=	–
14:94206394:94378610	g.94378610C>T (GenBank: NC_000014.9) (c.1096G>A [GenBank: NM_000295.5] [p.Glu366Gln])	1.63e−7 (MR-MEGA)	SERPINA1	p.Glu366Gln	24.2
11:64800220:64813774	g.64805130G>A (GenBank: NC_000011.10) (c.1269C>T [GenBank: NM_000244.4] [p.Asp423Glu])	2.25e−6 (MR-MEGA)	MEN1	p.Asp423Glu	19.25

Posterior probabilities were calculated from Bayes scores from MR-MEGA meta-analysis results for all significant and suggestive loci. Loci were filtered for those with fewer than five variants per 95% credible set. Ensembl Variant Effect Predictor was used to identify common coding variants from those credible sets. CADD, combined annotation-dependent depletion score; CHR, chromosome.

tumor-suppressor properties,[46] and *HABP4* (MIM: 617369), which is implicated in familial colorectal cancer.[47]

Gene set analysis shows enrichment in liver

We ran MAGMA for Gene Ontology and tissue-level and single-cell expression data.[33] Enriched Gene Ontology sets in MSigDB v.7.0 were dominated by those related to antigen presentation given mapping to multiple HLA genes (Figure S4A and Table S8). In addition, we observed enrichment of the β-catenin-TCF transactivating complex based on *TERT*, *MEN1*, and histone genes (p-adj = 0.03) and GWAS Catalog signatures related to chronic liver disease. Differentially expressed genes were preferentially expressed in liver tissue, based on GTEx data (Figure S4B).

HCC shares genetic architecture with chronic liver disease but not other gastrointestinal cancers

Finally, we used LD score regression to quantify the similarity in genetic architecture between HCC, liver disorders, and other gastrointestinal cancers (Figure 5). There was a strong positive genomic correlation between HCC and cirrhosis (rg = 0.87, $p = 5.0 \times 10^{-10}$), HBV (rg = 0.84, $p = 0.003$), HCV (rg = 0.65, $p = 0.004$), and MASLD (rg = 0.50, $p = 6.0 \times 10^{-4}$), reflecting the underlying drivers of HCC in the population. There was no significant correlation between HCC and upper GI cancer (esophageal and gastric) or colorectal cancer. There were shared genetic determinants between HCC and T2DM (rg = 0.25, $p = 6.8 \times 10^{-5}$), while there was inverse association between HCC and BMI (rg = −0.17, $p = 6.0 \times 10^{-4}$), suggesting that genetically lower BMI is linked to increased genetic risk of HCC.

Discussion

This large GWAS meta-analysis of all-cause HCC has discovered evidence for germline involvement of several genes involved in HCC, most notably 8q24 (nearest gene *MYC*) and *MAP3K9*. We also found genome-wide

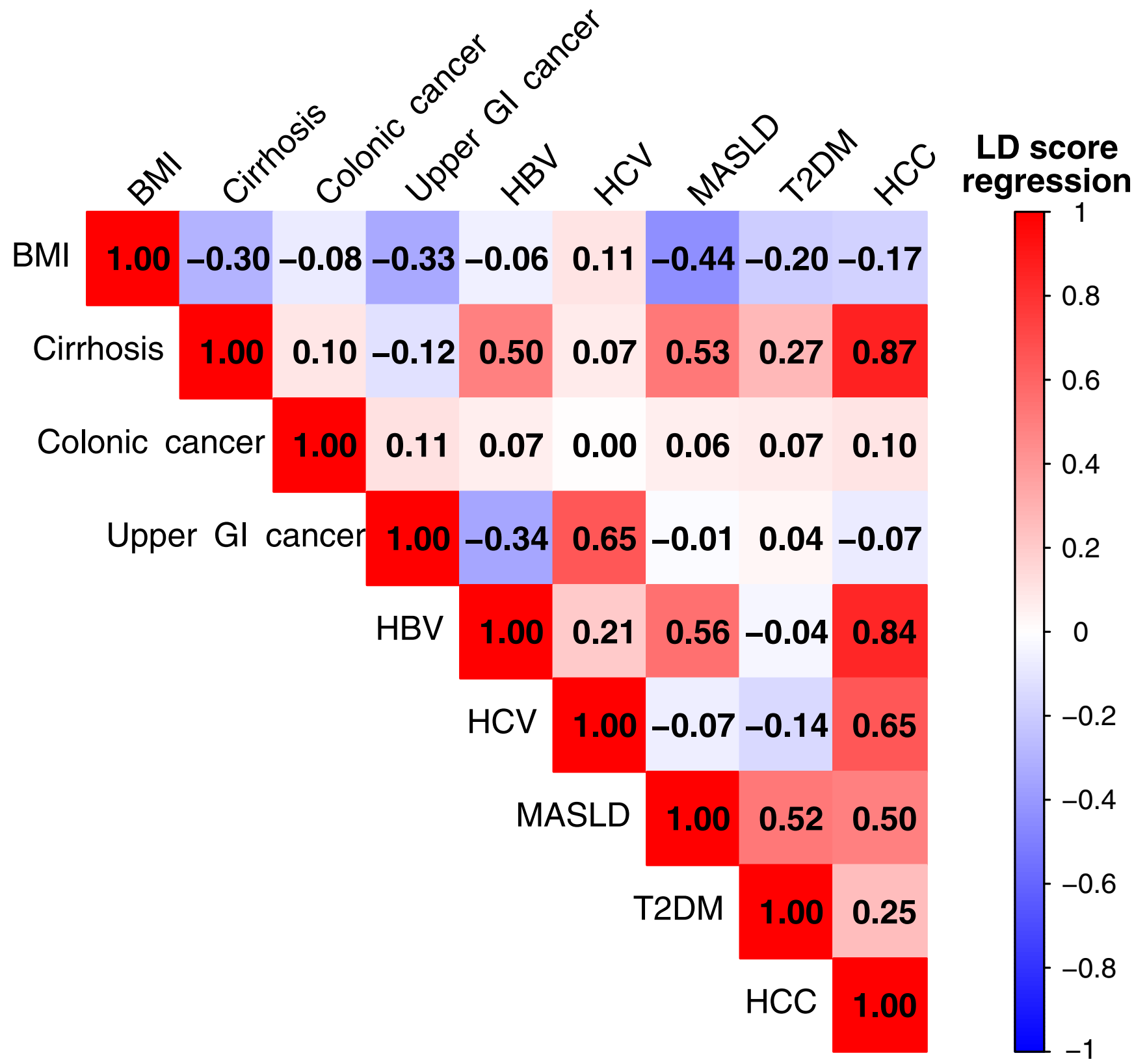

Figure 5. Similarity in genetic architecture between HCC and other gastrointestinal traits
LD score regression results between HCC (from this study) and previously published GWASs for chronic liver disease,[16,19,29,36] metabolic disease (type 2 diabetes mellitus [T2DM],[38] body mass index [BMI],[37] and upper gastrointestinal [GI] cancer [esophageal and gastric]),[39] and colorectal carcinoma.[40]

significant germline associations for HCC in *GCKR*, *ADH5*, and *MTTP*. In addition, we have dissected diverse effects across ancestries and have leveraged these results to fine-map 11 missense variants. Finally, we demonstrate the importance of germline risk for cirrhosis, T2DM, and low BMI as drivers of population risk for HCC.

The 8q24.21 locus has been recognized as a gene desert harboring multiple independent cancer risk loci for prostate,[48,49] colorectal,[50] and breast cancer, none of which align to protein-coding genes.[51,52] Functional studies have demonstrated that these loci act as tissue-specific long-range enhancers that physically interact with *MYC* through chromatin looping over several hundred kilobases. The independent signals in different cancer types are not in LD with each other, illustrating that this region harbors multiple tissue-specific regulatory elements. This is of particular relevance in HCC, as *MYC* is a transcription factor that lies downstream of multiple cell-surface signaling pathways, including the WNT-β-catenin pathway,[53] which is robustly implicated in HCC development.[54–56] Variation in 8q24 *MYC* confers increased susceptibility to *WNT* signaling in other malignancies.[57] We propose that rs1561929 represents an HCC-specific signal at 8q24 operating through an analogous mechanism. It is unclear whether its effect is dependent on cirrhosis or not; there was no evidence of colocalization, but it was not significant on sensitivity analysis using only cirrhotic controls.

The mitogen-activated protein kinase kinase kinases (MAP3K) are a group of proteins that act downstream of cellular stress signals and growth factor receptors. They function as part of a signaling cascade that ultimately leads to pro-growth effects. *MAP3K9* primarily transduces cellular stress through to *JUN* and *GATA4*, which influences mitochondrial cytochrome *c* release and, therefore, apoptosis.[58,59] Mutations in *MAP3K9* have been identified in lung cancer and melanoma,[60,61] as well as in HCC, via the TCGA dataset. It is not known precisely which stress signals regular *MAP3K9* in the liver, although interactions with a variety of microRNAs have been proposed.[62,63]

Prevalence of HCC varies greatly across the globe, from 1 in 100,000 in Morocco to 16 in 100,000 in the Republic of Korea.[64] While this is driven by differences in the prevalence of chronic viral hepatitis, alcohol intake, and obesity, our analysis has uncovered ancestral heterogeneity in germline contribution to HCC risk. Specific HLA loci showed divergent effects in European and East Asian ancestries. Also, in individuals of East Asian ancestry, we observed no significant effect from *TM6SF2*, which was the most significant locus in Europeans.[65] Some of these differences may reflect the underlying etiology of HCC in these regions (i.e., viral hepatitis versus steatotic liver disease).

Genetically driven accumulation of hepatic lipid droplets has been associated with severity of almost every studied liver disease. In this analysis, we found evidence for six genes (*MTTP*, *PNPLA3*, *TM6SF2*, *HSD17B13*, *GCKR*, and *APOE*) that are all genome-wide risk genes for MASLD.[10] Gene-environment studies have previously shown that the effect of such variants is amplified by the presence of obesity, T2DM, and excess alcohol consumption.[66] The stronger effects observed in European cohorts may reflect the higher prevalence of steatotic liver disease in these populations.[67] It should also be noted that non-cirrhotic HCC is well described in steatotic liver disease.[68] These data underscore the importance of public health interventions to tackle HCC.

The principal strengths of this study are its size and TWAS/RWAS analyses. The integration of GWAS, TWAS, and RWAS provides three complementary layers of functional evidence. *EPHA2* was identified in both the GWAS and RWAS, providing independent genomic and regulatory support. *DHRS1* emerged specifically through TWAS, demonstrating that germline-encoded transcriptomic variation can identify candidate genes not captured by positional mapping alone. The *ZNF367*/*HABP4* locus at chromosome 9 was identified exclusively through RWAS, representing a chromatin-accessible region that would not have been detected by a GWAS or TWAS. Further mechanistic work is required to understand the function of these loci in HCC development.

This study would be improved by inclusion of individual-participant-level data, which would allow for mediation analyses as performed by Vujkovic et al.[7] Also, most cohorts used electronic health records for identification of cases, which carries the risk of misclassification. GWAS in the All of Us cohort was performed using Wald logistic regression, which can produce inflated test statistics under case/control imbalance. Although the genomic inflation for this cohort was nominal ($\lambda = 1.008$) and All of Us contributed no genome-wide significant loci to the meta-analysis, future analyses should consider Firth correction or saddlepoint approximation methods. In addition, we have been unable to robustly identify the causal gene at 8q24.21. We propose that *MYC* is the most likely gene for the rs1561929 variant, but functional work is required to establish this.

Despite the inclusion of multiple ancestries, individuals of European ancestry are still over-represented and account for 49% of HCC cases in this analysis. Individuals of African descent are particularly under-represented, with only 6% of all affected individuals and no data from the continent of Africa, which has some of the highest rates of HCC.[69] We are also lacking data from the Middle East and South America, where HCC are major causes of cancer death. Addition of data from population biobank studies from these areas will strengthen future MAMA.

Most cohorts included in this analysis had healthy population controls; therefore, this meta-analysis reflects the population-level drivers of HCC, and many loci are already implicated in the development of underlying liver disease (e.g., *TM6SF2*). Our sensitivity analysis with cirrhosis controls and colocalization studies demonstrated that most significant loci, including *MAP3K9*, appear to act independently of cirrhosis. Larger meta-analyses with cirrhotic control individuals or etiology-specific studies may identify further treatment targets.

Conclusion

HCC is driven by heterogeneous and homogeneous genetic factors across ancestries. Germline susceptibility to β-catenin pathway activation influences HCC risk via *MEN1*, *TERT*, and, potentially, the 8q24 locus (mediated by *MYC*). MAP3K9 and *DHRS1* are HCC risk genes worthy of further investigation. Genes involved in hepatic lipid droplet formation, including *MTTP*, contribute to the risk of HCC. There is a need for further ancestral diversity in liver GWASs.

Data code and availability

Raw data for Hassan et al.[5] are available via dbGaP phs001744.v1.p1. All other summary statistics are publicly available via the aforementioned links. Full summary statistics from this analysis can be found on GWAS Catalog (https://www.ebi.ac.uk/gwas/home) using the accession numbers NHGRI: GCST90860789, GCST90860790, GCST90860791, and GCST90860792. All code used in analysis is available at https://github.com/jmann01/HCC-gwas.

Acknowledgments

J.P.M. is supported by grants from Birmingham Health Partners, NIHR ACL (CL-2022-09-005), The Royal Society (RG\R1\241312), BSPGHAN-Guts (BSPGHAN2023_01), ESPGHAN, Royal College of Physicians Dame Sheila Sherlock Bursary, and Little Princess Trust (CCLGA 2024 10 Mann). S.S. is supported by a Cancer Research UK Advanced Clinician Scientist Fellowship (C53575/A29959). We thank all the participants, investigators, and funders who contributed to the studies included in this meta-analysis. The research was carried out at the National Institute for Health and Care Research (NIHR) Birmingham Biomedical Research Centre (BRC).

Declaration of interests

V.L.C. received grants from AstraZeneca, Takeda, Ipsen, and KOWA, paid to the University of Michigan. S.S. has received honorarium from Astra Zeneca.

Supplemental information

Supplemental information can be found online at https://doi.org/10.1016/j.xhgg.2026.100639.

Web resources

China Kadoorie Biobank (CKBB) PheWeb, https://pheweb.ckbiobank.org/download/c22

deCODE/UK Biobank/Intermountain summary statistics, https://www.decode.com/summarydata/

DepMap: The Cancer Dependency Map, https://depmap.org/portal/

FinnGen summary statistics, https://www.finngen.fi/en

GenBank, https://www.ncbi.nlm.nih.gov/genbank/

GWAS Catalog, https://www.ebi.ac.uk/gwas/

gwasglue, https://github.com/MRCIEU/gwasglue

Hail genomic analysis framework, https://github.com/hail-is/hail

HCC GWAS analysis code (this study), https://github.com/jmann01/HCC-gwas

Korean BioBank (KoGES) summary statistics, https://koges.leelabsg.org/

Million Veterans Program (MVP) summary statistics, https://ftp.ncbi.nlm.nih.gov/dbgap/studies/phs002453/analyses/GIA/

Online Mendelian Inheritance in Man (OMIM), https://www.omim.org

PLCO Atlas, https://exploregwas.cancer.gov/plco-atlas

Taiwan Precision Medicine Initiative (TPMI) PheWeb, https://pheweb.ibms.sinica.edu.tw/pheno/155.1

Received: February 27, 2026
Accepted: June 22, 2026

References

1. Bray, F., Laversanne, M., Sung, H., Ferlay, J., Siegel, R.L., Soerjomataram, I., and Jemal, A. (2024). Global cancer statistics 2022: GLOBOCAN estimates of incidence and mortality worldwide for 36 cancers in 185 countries. CA Cancer J. Clin. *74*, 229–263.
2. Singal, A.G., Kanwal, F., and Llovet, J.M. (2023). Global trends in hepatocellular carcinoma epidemiology: implications for screening, prevention and therapy. Nat. Rev. Clin. Oncol. *20*, 864–884.
3. Llovet, J.M., Pinyol, R., Kelley, R.K., El-Khoueiry, A., Reeves, H.L., Wang, X.W., Gores, G.J., and Villanueva, A. (2022). Molecular pathogenesis and systemic therapies for hepatocellular carcinoma. Nat. Cancer *3*, 386–401.
4. Trépo, E., Caruso, S., Yang, J., Imbeaud, S., Couchy, G., Bayard, Q., Letouzé, E., Ganne-Carrié, N., Moreno, C., Oussalah, A., et al. (2022). Common genetic variation in alcohol-related hepatocellular carcinoma: a case-control genome-wide association study. Lancet Oncol. *23*, 161–171.
5. Hassan, M.M., Li, D., Han, Y., Byun, J., Hatia, R.I., Long, E., Choi, J., Kelley, R.K., Cleary, S.P., Lok, A.S., et al. (2024). Genome-wide association study identifies high-impact susceptibility loci for HCC in North America. Hepatology *80*, 87–101.
6. Ghouse, J., Gellert-Kristensen, H., O'Rourke, C.J., Seidelin, A.-S., Thorleifsson, G., Sveinbjörnsson, G., Tragante, V., Konkwo, C., Brancale, J., Vilarinho, S., et al. (2025). Genome-wide meta-analysis identifies nine loci associated with higher risk of hepatocellular carcinoma development. JHEP Rep. *7*, 101485.
7. Vujkovic, M., Kaplan, D.E., Ghouse, J., Loza, B.-L., Brancale, J., Lewis, A., Zhang, D.Y., Levin, M.G., Veatch, O.J., Johnson, J.P., et al. (2025). Germline variants influence chronic liver disease progression through distinct pathways. Preprint at medRxiv. https://doi.org/10.1101/2025.09.16.25335186.
8. Verweij, N., Haas, M.E., Nielsen, J.B., Sosina, O.A., Kim, M., Akbari, P., De, T., Hindy, G., Bovijn, J., Persaud, T., et al. (2022). Germline Mutations in CIDEB and Protection against Liver Disease. N. Engl. J. Med. *387*, 332–344.
9. Emdin, C.A., Haas, M.E., Khera, A.V., Aragam, K., Chaffin, M., Klarin, D., Hindy, G., Jiang, L., Wei, W.Q., Feng, Q., et al. (2020). A missense variant in Mitochondrial Amidoxime Reducing Component 1 gene and protection against liver disease. PLoS Genet. *16*, e1008629.
10. Chen, Y., Du, X., Kuppa, A., Feitosa, M.F., Bielak, L.F., O'Connell, J.R., Musani, S.K., Guo, X., Kahali, B., Chen, V.L., et al. (2023). Genome-wide association meta-analysis identifies 17 loci associated with nonalcoholic fatty liver disease. Nat. Genet. *55*, 1640–1650.
11. Buch, S., Innes, H., Lutz, P.L., Nischalke, H.D., Marquardt, J.U., Fischer, J., Weiss, K.H., Rosendahl, J., Marot, A., Krawczyk, M., et al. (2023). Genetic variation in TERT modifies the risk of hepatocellular carcinoma in alcohol-related cirrhosis: results from a genome-wide case-control study. Gut *72*, 381–391.
12. Sveinbjornsson, G., Ulfarsson, M.O., Thorolfsdottir, R.B., Jonsson, B.A., Einarsson, E., Gunnlaugsson, G., Rognvaldsson, S., Arnar, D.O., Baldvinsson, M., Bjarnason, R.G., et al. (2022). Multiomics study of nonalcoholic fatty liver disease. Nat. Genet. *54*, 1652–1663.
13. Yang, H.-C., Kwok, P.-Y., Li, L.-H., Liu, Y.-M., Jong, Y.-J., Lee, K.-Y., Wang, D.W., Tsai, M.F., Yang, J.H., Chen, C.H., et al. (2025). The Taiwan Precision Medicine Initiative provides a cohort for large-scale studies. Nature *648*, 117–127.
14. Chen, H.-H., Chen, C.-H., Hou, M.-C., Fu, Y.-C., Li, L.-H., Chou, C.-Y., Yeh, E.C., Tsai, M.F., Chen, C.H., Yang, H.C., et al. (2025). Population-specific polygenic risk scores for people of Han Chinese ancestry. Nature *648*, 128–137.
15. Walters, R.G., Millwood, I.Y., Lin, K., Schmidt Valle, D., McDonnell, P., Hacker, A., Avery, D., Edris, A., Fry, H., Cai, N., et al. (2023). Genotyping and population characteristics of the China Kadoorie Biobank. Cell Genom. *3*, 100361.
16. Sakaue, S., Kanai, M., Tanigawa, Y., Karjalainen, J., Kurki, M., Koshiba, S., Narita, A., Konuma, T., Yamamoto, K., Akiyama, M., et al. (2021). A cross-population atlas of genetic associations for 220 human phenotypes. Nat. Genet. *53*, 1415–1424.
17. Nam, K., Kim, J., and Lee, S. (2022). Genome-wide study on 72,298 individuals in Korean biobank data for 76 traits. Cell Genom. *2*, 100189.
18. Machiela, M.J., Huang, W.-Y., Wong, W., Berndt, S.I., Sampson, J., De Almeida, J., Abubakar, M., Hislop, J., Chen, K.L., Dagnall, C., et al. (2023). GWAS Explorer: an open-source tool to explore, visualize, and access GWAS summary statistics in the PLCO Atlas. Sci. Data *10*, 25.
19. Verma, A., Huffman, J.E., Rodriguez, A., Conery, M., Liu, M., Ho, Y.-L., Kim, Y., Heise, D.A., Guare, L., Panickan, V.A., et al. (2024). Diversity and scale: Genetic architecture of 2068 traits in the VA Million Veteran Program. Science *385*, eadj1182.
20. All of Us Research Program Genomics Investigators (2024). Genomic data in the All of Us Research Program. Nature *627*, 340–346.
21. All of Us Research Program Investigators, Denny, J.C., Rutter, J.L., Goldstein, D.B., Philippakis, A., Smoller, J.W., Jenkins, G., and Dishman, E. (2019). The "All of Us" Research Program. N. Engl. J. Med. *381*, 668–676.
22. Murphy, A.E., Schilder, B.M., and Skene, N.G. (2021). MungeSumstats: a Bioconductor package for the standardization and quality control of many GWAS summary statistics. Bioinformatics *37*, 4593–4596.
23. GitHub - hail-is/hail. Cloud-native genomic dataframes and batch computing [Internet]. GitHub. https://github.com/hail-is/hail.
24. Mägi, R., Horikoshi, M., Sofer, T., Mahajan, A., Kitajima, H., Franceschini, N., McCarthy, M.I., COGENT-Kidney Consortium, T2D-GENES Consortium, and Morris, A.P. (2017). Trans-ethnic meta-regression of genome-wide association studies accounting for ancestry increases power for discovery and improves fine-mapping resolution. Hum. Mol. Genet. *26*, 3639–3650.

25. Willer, C.J., Li, Y., and Abecasis, G.R. (2010). METAL: fast and efficient meta-analysis of genomewide association scans. Bioinformatics *26*, 2190–2191.
26. R Core Team (2019). A Language and Environment for Statistical Computing (Vienna, Austria: R Foundation for Statistical Computing).
27. Watanabe, K., Taskesen, E., van Bochoven, A., and Posthuma, D. (2017). Functional mapping and annotation of genetic associations with FUMA. Nat. Commun. *8*, 1826.
28. Watanabe, K., Umićević Mirkov, M., de Leeuw, C.A., van den Heuvel, M.P., and Posthuma, D. (2019). Genetic mapping of cell type specificity for complex traits. Nat. Commun. *10*, 3222.
29. Ghouse, J., Sveinbjörnsson, G., Vujkovic, M., Seidelin, A.-S., Gellert-Kristensen, H., Ahlberg, G., Tragante, V., Rand, S.A., Brancale, J., Vilarinho, S., et al. (2024). Integrative common and rare variant analyses provide insights into the genetic architecture of liver cirrhosis. Nat. Genet. *56*, 827–837.
30. GitHub - MRCIEU/gwasglue. Linking GWAS data to analytical tools in R [Internet]. GitHub. https://github.com/MRCIEU/gwasglue.
31. Kim, J.J., Vitale, D., Otani, D.V., Lian, M.M., Heilbron, K., Iwaki, H., Lake, J., Solsberg, C.W., Leonard, H., Makarious, M.B., et al. (2024). Multi-ancestry genome-wide association meta-analysis of Parkinson's disease. Nat. Genet. *56*, 27–36.
32. McLaren, W., Gil, L., Hunt, S.E., Riat, H.S., Ritchie, G.R.S., Thormann, A., Flicek, P., and Cunningham, F. (2016). The Ensembl Variant Effect Predictor. Genome Biol. *17*, 122.
33. de Leeuw, C.A., Mooij, J.M., Heskes, T., and Posthuma, D. (2015). MAGMA: generalized gene-set analysis of GWAS data. PLoS Comput. Biol. *11*, e1004219.
34. Gusev, A., Ko, A., Shi, H., Bhatia, G., Chung, W., Penninx, B.W.J.H., Jansen, R., de Geus, E.J.C., Boomsma, D.I., Wright, F.A., et al. (2016). Integrative approaches for large-scale transcriptome-wide association studies. Nat. Genet. *48*, 245–252.
35. Grishin, D., and Gusev, A. (2022). Allelic imbalance of chromatin accessibility in cancer identifies candidate causal risk variants and their mechanisms. Nat. Genet. *54*, 837–849.
36. Liu, Y., Basty, N., Whitcher, B., Bell, J.D., Sorokin, E.P., van Bruggen, N., Thomas, E.L., and Cule, M. (2021). Genetic architecture of 11 organ traits derived from abdominal MRI using deep learning. eLife *10*, e65554. https://doi.org/10.7554/eLife.65554.
37. Pulit, S.L., Stoneman, C., Morris, A.P., Wood, A.R., Glastonbury, C.A., Tyrrell, J., Yengo, L., Ferreira, T., Marouli, E., Ji, Y., et al. (2019). Meta-Analysis of genome-wide association studies for body fat distribution in 694 649 individuals of European ancestry. Hum. Mol. Genet. *28*, 166–174.
38. Huerta-Chagoya, A., Schroeder, P., Mandla, R., Li, J., Morris, L., Vora, M., Alkanaq, A., Nagy, D., Szczerbinski, L., Madsen, J.G.S., et al. (2024). Rare variant analyses in 51,256 type 2 diabetes cases and 370,487 controls reveal the pathogenicity spectrum of monogenic diabetes genes. Nat. Genet. *56*, 2370–2379.
39. Rashkin, S.R., Graff, R.E., Kachuri, L., Thai, K.K., Alexeeff, S.E., Blatchins, M.A., Cavazos, T.B., Corley, D.A., Emami, N.C., Hoffman, J.D., et al. (2020). Pan-cancer study detects genetic risk variants and shared genetic basis in two large cohorts. Nat. Commun. *11*, 4423. https://doi.org/10.1038/s41467-020-18246-6.
40. Fernandez-Rozadilla, C., Timofeeva, M., Chen, Z., Law, P., Thomas, M., Schmit, S., Díez-Obrero, V., Hsu, L., Fernandez-Tajes, J., Palles, C., et al. (2023). Deciphering colorectal cancer genetics through multi-omic analysis of 100,204 cases and 154,587 controls of European and east Asian ancestries. Nat. Genet. *55*, 89–99.
41. DepMap Portal. DepMap: The Cancer Dependency Map Project at Broad Institute. https://depmap.org/portal/.
42. Chen, S., Francioli, L.C., Goodrich, J.K., Collins, R.L., Kanai, M., Wang, Q., Alföldi, J., Watts, N.A., Vittal, C., Gauthier, L.D., et al. (2024). A genomic mutational constraint map using variation in 76,156 human genomes. Nature *625*, 92–100.
43. Gealekman, O., Burkart, A., Chouinard, M., Nicoloro, S.M., Straubhaar, J., and Corvera, S. (2008). Enhanced angiogenesis in obesity and in response to PPARgamma activators through adipocyte VEGF and ANGPTL4 production. Am. J. Physiol. Endocrinol. Metab. *295*, E1056–E1064.
44. Mysling, S., Kristensen, K.K., Larsson, M., Kovrov, O., Bensadouen, A., Jørgensen, T.J., Olivecrona, G., Young, S.G., and Ploug, M. (2016). The angiopoietin-like protein ANGPTL4 catalyzes unfolding of the hydrolase domain in lipoprotein lipase and the endothelial membrane protein GPIHBP1 counteracts this unfolding. eLife *5*, e20958. https://doi.org/10.7554/eLife.20958.
45. Zemanová, L., Navrátilová, H., Andrýs, R., Šperková, K., Andrejs, J., Kozáková, K., Meier, M., Möller, G., Novotná, E., Šafr, M., et al. (2019). Initial characterization of human DHRS1 (SDR19C1), a member of the short-chain dehydrogenase/reductase superfamily. J. Steroid Biochem. Mol. Biol. *185*, 80–89.
46. Jain, M., Zhang, L., Boufraqech, M., Liu-Chittenden, Y., Bussey, K., Demeure, M.J., Wu, X., Su, L., Pacak, K., Stratakis, C.A., and Kebebew, E. (2014). ZNF367 inhibits cancer progression and is targeted by miR-195. PLoS One *9*, e101423.
47. Gray-McGuire, C., Guda, K., Adrianto, I., Lin, C.P., Natale, L., Potter, J.D., Newcomb, P., Poole, E.M., Ulrich, C.M., Lindor, N., et al. (2010). Confirmation of linkage to and localization of familial colon cancer risk haplotype on chromosome 9q22. Cancer Res. *70*, 5409–5418.
48. Yeager, M., Orr, N., Hayes, R.B., Jacobs, K.B., Kraft, P., Wacholder, S., Minichiello, M.J., Fearnhead, P., Yu, K., Chatterjee, N., et al. (2007). Genome-wide association study of prostate cancer identifies a second risk locus at 8q24. Nat. Genet. *39*, 645–649.
49. Haiman, C.A., Patterson, N., Freedman, M.L., Myers, S.R., Pike, M.C., Waliszewska, A., Neubauer, J., Tandon, A., Schirmer, C., McDonald, G.J., et al. (2007). Multiple regions within 8q24 independently affect risk for prostate cancer. Nat. Genet. *39*, 638–644.
50. Pomerantz, M.M., Ahmadiyeh, N., Jia, L., Herman, P., Verzi, M.P., Doddapaneni, H., Beckwith, C.A., Chan, J.A., Hills, A., Davis, M., et al. (2009). The 8q24 cancer risk variant rs6983267 shows long-range interaction with MYC in colorectal cancer. Nat. Genet. *41*, 882–884.
51. Ghoussaini, M., Song, H., Koessler, T., Al Olama, A.A., Kote-Jarai, Z., Driver, K.E., Pooley, K.A., Ramus, S.J., Kjaer, S.K., Hogdall, E., et al. (2008). Multiple loci with different cancer specificities within the 8q24 gene desert. J. Natl. Cancer Inst. *100*, 962–966.
52. Jia, L., Landan, G., Pomerantz, M., Jaschek, R., Herman, P., Reich, D., Yan, C., Khalid, O., Kantoff, P., Oh, W., et al. (2009). Functional enhancers at the gene-poor 8q24 cancer-linked locus. PLoS Genet. *5*, e1000597.

53. Dang, C.V. (2012). MYC on the path to cancer. Cell *149*, 22–35.
54. Calvisi, D.F., Conner, E.A., Ladu, S., Lemmer, E.R., Factor, V.M., and Thorgeirsson, S.S. (2005). Activation of the canonical Wnt/beta-catenin pathway confers growth advantages in c-Myc/E2F1 transgenic mouse model of liver cancer. J. Hepatol. *42*, 842–849.
55. Kim, M., Lee, H.C., Tsedensodnom, O., Hartley, R., Lim, Y.-S., Yu, E., Merle, P., and Wands, J.R. (2008). Functional interaction between Wnt3 and Frizzled-7 leads to activation of the Wnt/beta-catenin signaling pathway in hepatocellular carcinoma cells. J. Hepatol. *48*, 780–791.
56. Bisso, A., Filipuzzi, M., Gamarra Figueroa, G.P., Brumana, G., Biagioni, F., Doni, M., Ceccotti, G., Tanaskovic, N., Morelli, M.J., Pendino, V., et al. (2020). Cooperation Between MYC and β-Catenin in Liver Tumorigenesis Requires Yap/Taz. Hepatology *72*, 1430–1443.
57. Tuupanen, S., Turunen, M., Lehtonen, R., Hallikas, O., Vanharanta, S., Kivioja, T., Björklund, M., Wei, G., Yan, J., Niittymäki, I., et al. (2009). The common colorectal cancer predisposition SNP rs6983267 at chromosome 8q24 confers potential to enhanced Wnt signaling. Nat. Genet. *41*, 885–890. https://doi.org/10.1038/ng.406.
58. Durkin, J.T., Holskin, B.P., Kopec, K.K., Reed, M.S., Spais, C.M., Steffy, B.M., Gessner, G., Angeles, T.S., Pohl, J., Ator, M.A., and Meyer, S.L. (2004). Phosphoregulation of mixed-lineage kinase 1 activity by multiple phosphorylation in the activation loop. Biochemistry *43*, 16348–16355.
59. Xu, Z., Maroney, A.C., Dobrzanski, P., Kukekov, N.V., and Greene, L.A. (2001). The MLK family mediates c-Jun N-terminal kinase activation in neuronal apoptosis. Mol. Cell Biol. *21*, 4713–4724.
60. Fawdar, S., Trotter, E.W., Li, Y., Stephenson, N.L., Hanke, F., Marusiak, A.A., Edwards, Z.C., Ientile, S., Waszkowycz, B., Miller, C.J., and Brognard, J. (2013). Targeted genetic dependency screen facilitates identification of actionable mutations in FGFR4, MAP3K9, and PAK5 in lung cancer. Proc. Natl. Acad. Sci. USA *110*, 12426–12431.
61. Stark, M.S., Woods, S.L., Gartside, M.G., Bonazzi, V.F., Dutton-Regester, K., Aoude, L.G., Chow, D., Sereduk, C., Niemi, N.M., Tang, N., et al. (2011). Frequent somatic mutations in MAP3K5 and MAP3K9 in metastatic melanoma identified by exome sequencing. Nat. Genet. *44*, 165–169.
62. Jun, L., Xuhong, L., and Hui, L. (2023). Circ_SIPA1L1 promotes osteosarcoma progression via miR-379-5p/MAP3K9 axis. Cancer Biother. Radiopharm. *38*, 604–618.
63. Xia, J., Cao, T., Ma, C., Shi, Y., Sun, Y., Wang, Z.P., and Ma, J. (2018). MiR-7 suppresses tumor progression by directly targeting MAP3K9 in pancreatic cancer. Mol. Ther. Nucleic Acids *13*, 121–132.
64. Wu, T., Du, M., Zhang, T., Chen, X., and Liu, Z. (2026). Comparisons of global incidence and risk factor profiles of hepatocellular carcinoma and intrahepatic cholangiocarcinoma. Ann. Hepatol. *31*, 102139.
65. Kozlitina, J., Smagris, E., Stender, S., Nordestgaard, B.G., Zhou, H.H., Tybjærg-hansen, A., Vogt, T.F., Hobbs, H.H., and Cohen, J.C. (2014). Exone-wide association study identifies TM6SF2 variant that confers susceptibility to nonalcoholic fatty liver disease. Nat. Genet. *46*, 352–356.
66. Stender, S., Kozlitina, J., Nordestgaard, B.G., Tybjærg-Hansen, A., Hobbs, H.H., and Cohen, J.C. (2017). Adiposity amplifies the genetic risk of fatty liver disease conferred by multiple loci. Nat. Genet. *49*, 842–847.
67. Feng, G., Targher, G., Byrne, C.D., Yilmaz, Y., Wai-Sun Wong, V., Adithya Lesmana, C.R., Adams, L.A., Boursier, J., Papatheodoridis, G., El-Kassas, M., et al. (2025). Global burden of metabolic dysfunction-associated steatotic liver disease, 2010 to 2021. JHEP Rep. *7*, 101271.
68. Tan, D.J.H., Tamaki, N., Kim, B.K., Wijarnpreecha, K., Aboona, M.B., Faulkner, C., Kench, C., Salimi, S., Sabih, A.H., Lim, W.H., et al. (2025). Prevalence of low FIB-4 in MASLD-related hepatocellular carcinoma: A multicentre study. Aliment. Pharmacol. Ther. *61*, 278–285.
69. Tan, E.Y., Danpanichkul, P., Yong, J.N., Yu, Z., Tan, D.J.H., Lim, W.H., Koh, B., Lim, R.Y.Z., Tham, E.K.J., Mitra, K., et al. (2025). Liver cancer in 2021: Global burden of disease study. J. Hepatol. *82*, 851–860.

HGGA, Volume *7*

Supplemental information

Multi-ancestry genome-wide association meta-analysis of hepatocellular carcinoma identifies 15 risk loci including *MAP3K9*, *DHRS1*, *MTTP*, and 8q24.21

Ifechukwuamaka Chinaka, Annabelle Schofield, Christopher I. Amos, Lewis R. Roberts, Vincent L. Chen, Younghun Han, Manal Hassan, Shishir Shetty, and Jake P. Mann

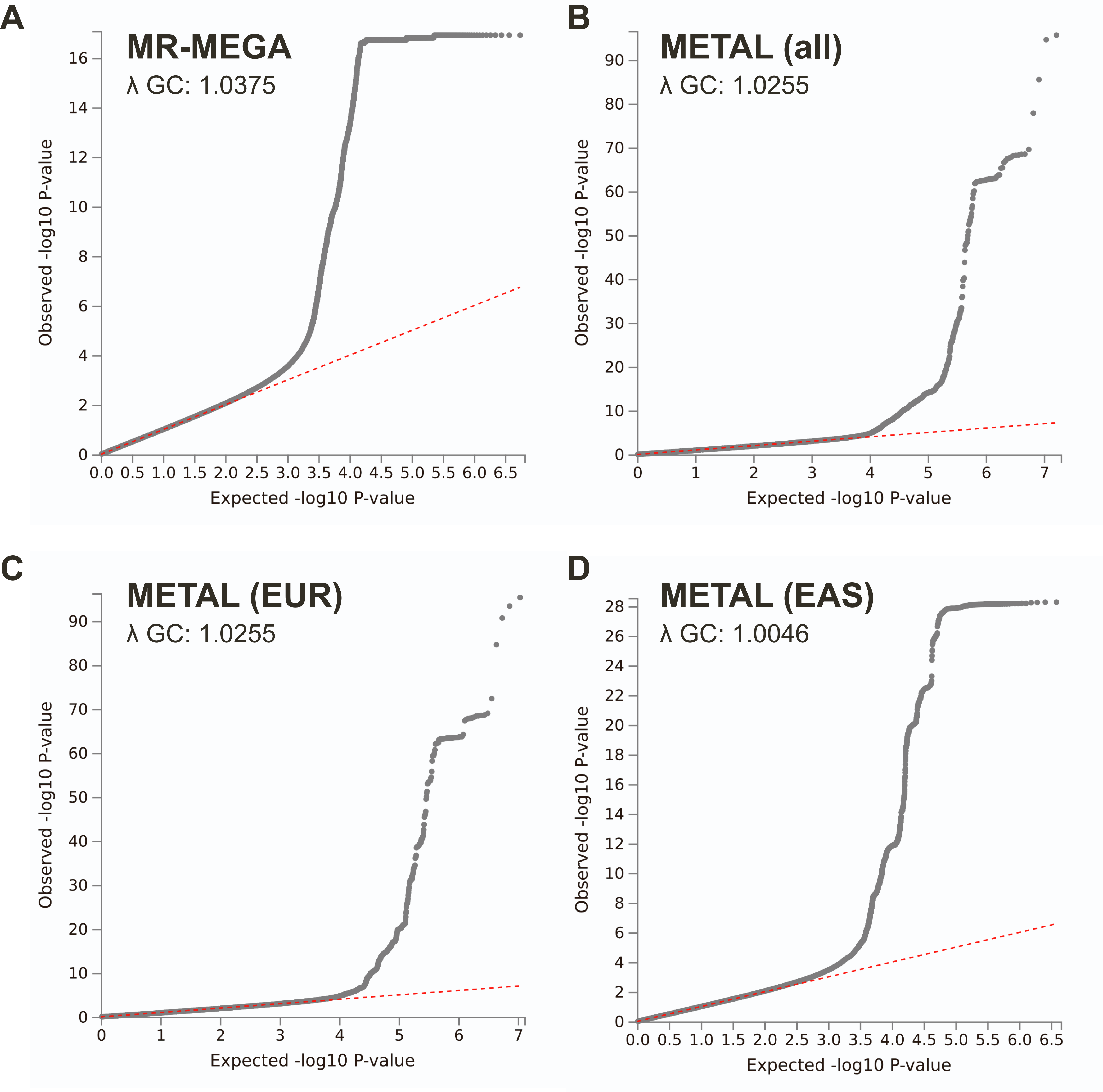

A
MR-MEGA
λ GC: 1.0375
B
METAL (all)
λ GC: 1.0255
C
METAL (EUR)
λ GC: 1.0255
D
METAL (EAS)
λ GC: 1.0046
Observed -log10 P-value
Expected -log10 P-value

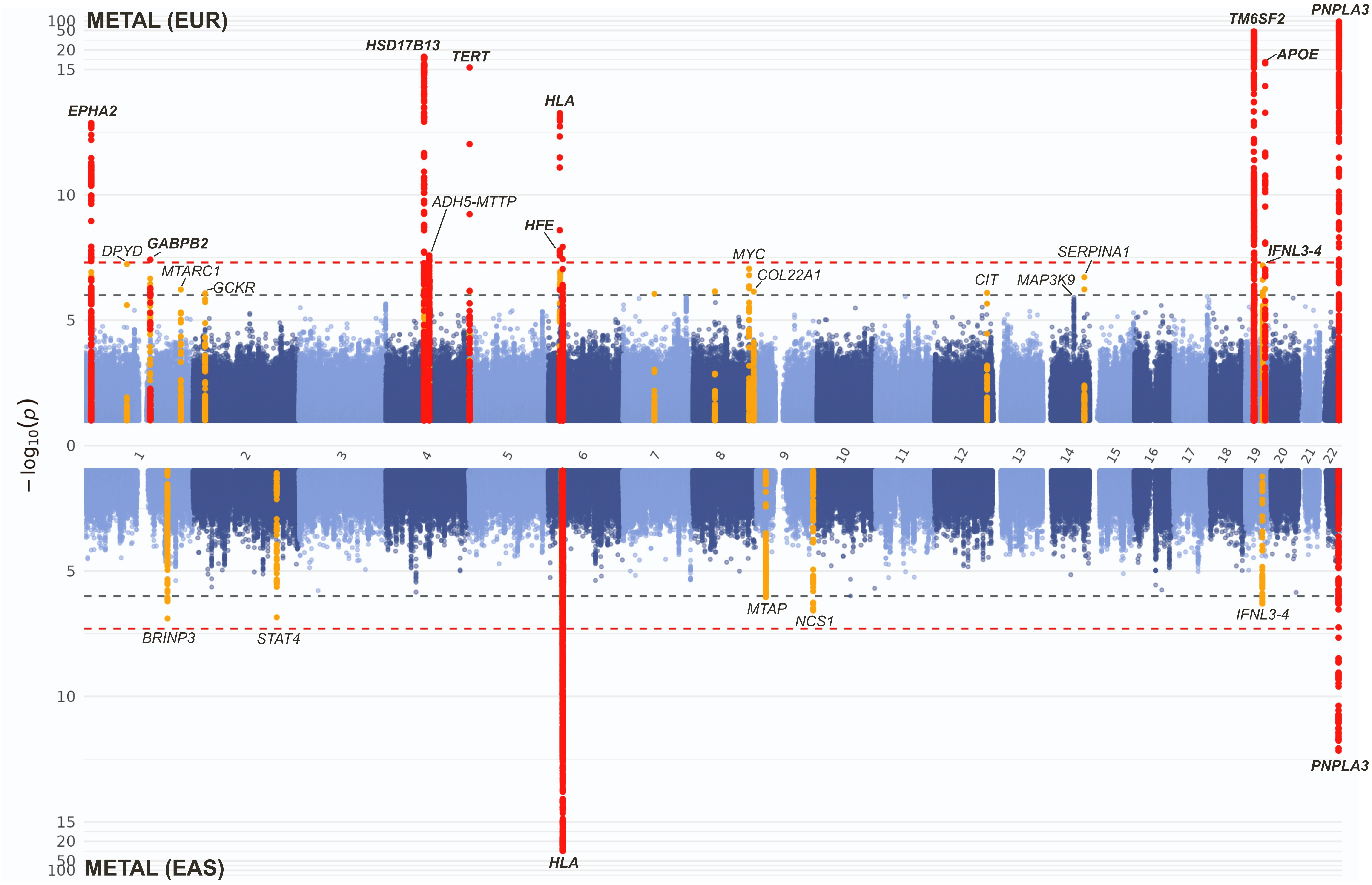
METAL (EUR)
METAL (EAS)
− log10(p)
EPHA2
DPYD
GABPB2
MTARC1
GCKR
HSD17B13
TERT
ADH5-MTTP
HLA
HFE
MYC
COL22A1
CIT
MAP3K9
SERPINA1
TM6SF2
APOE
IFNL3-4
PNPLA3
BRINP3
STAT4
MTAP
NCS1
1
2
3
4
5
6
7
8
9
10
11
12
13
14
15
16
17
18
19
20
21
22
0
5
10
15
20
50
100

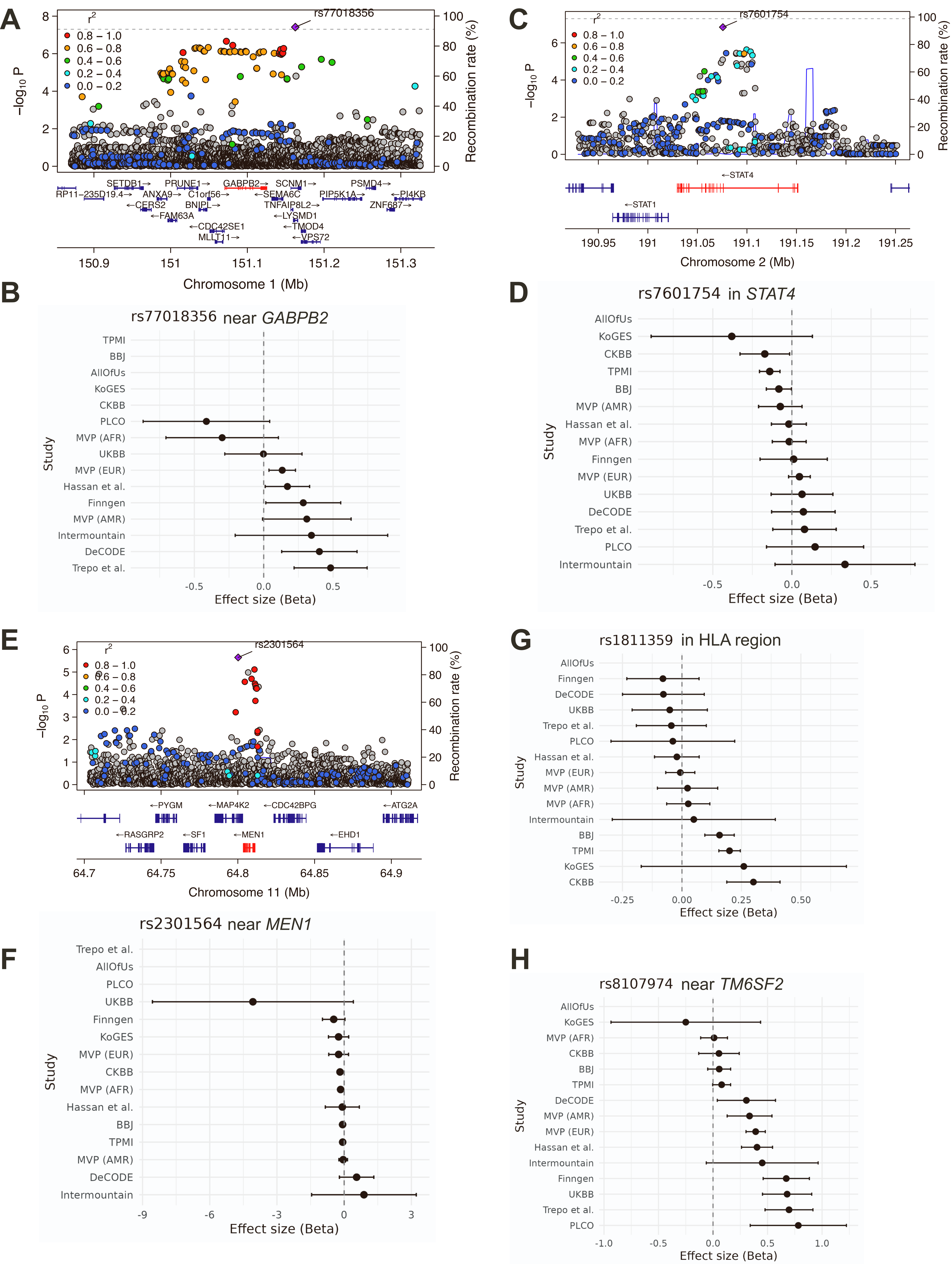
A
rs77018356
Chromosome 1 (Mb)
B
rs77018356 near GABPB2
Effect size (Beta)
Study
C
rs7601754
Chromosome 2 (Mb)
D
rs7601754 in STAT4
Effect size (Beta)
Study
E
rs2301564
Chromosome 11 (Mb)
F
rs2301564 near MEN1
Effect size (Beta)
Study
G
rs1811359 in HLA region
Effect size (Beta)
Study
H
rs8107974 near TM6SF2
Effect size (Beta)
Study

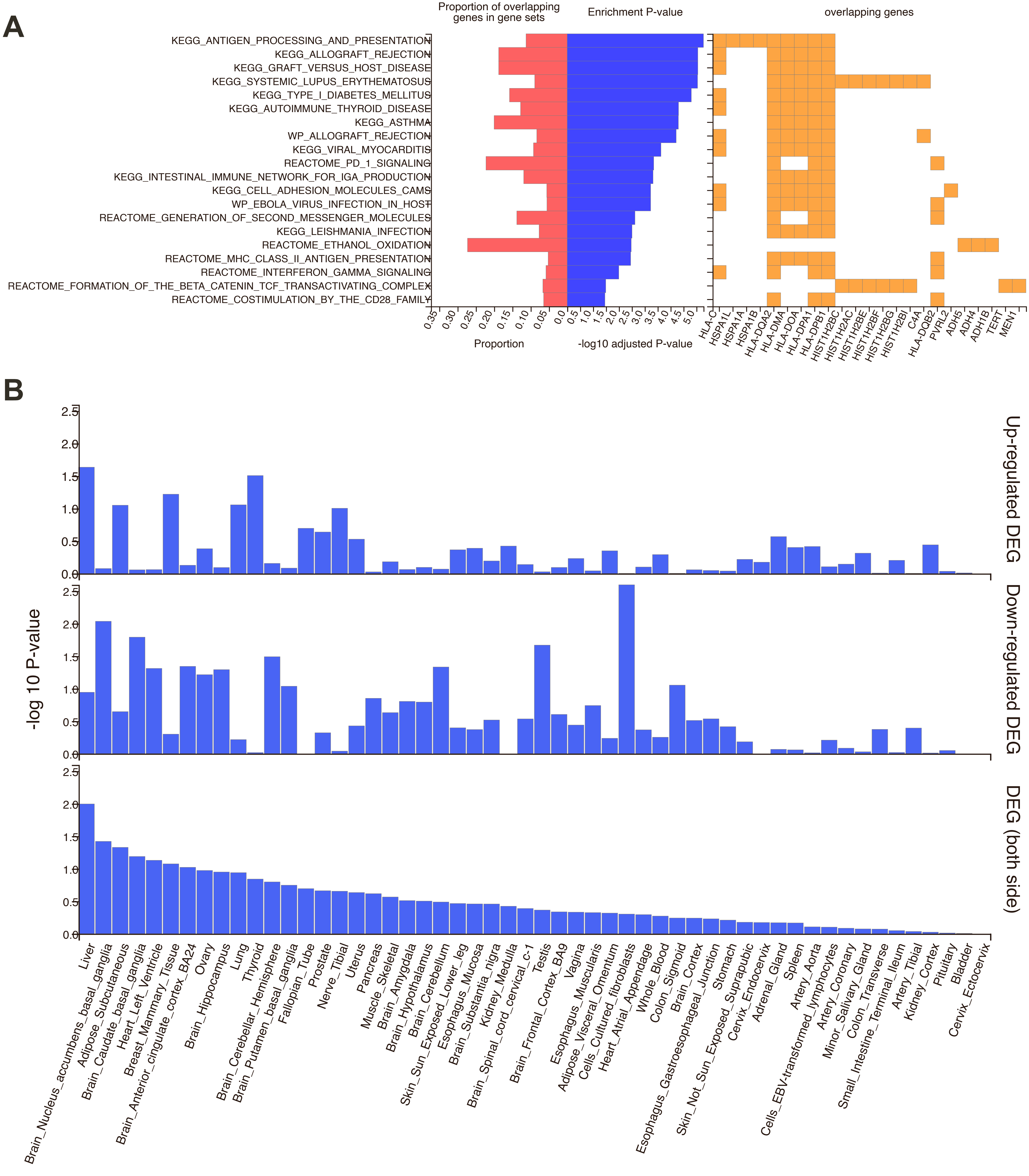
A
Proportion of overlapping genes in gene sets
Enrichment P-value
overlapping genes
KEGG_ANTIGEN_PROCESSING_AND_PRESENTATION
KEGG_ALLOGRAFT_REJECTION
KEGG_GRAFT_VERSUS_HOST_DISEASE
KEGG_SYSTEMIC_LUPUS_ERYTHEMATOSUS
KEGG_TYPE_I_DIABETES_MELLITUS
KEGG_AUTOIMMUNE_THYROID_DISEASE
KEGG_ASTHMA
WP_ALLOGRAFT_REJECTION
KEGG_VIRAL_MYOCARDITIS
REACTOME_PD_1_SIGNALING
KEGG_INTESTINAL_IMMUNE_NETWORK_FOR_IGA_PRODUCTION
KEGG_CELL_ADHESION_MOLECULES_CAMS
WP_EBOLA_VIRUS_INFECTION_IN_HOST
REACTOME_GENERATION_OF_SECOND_MESSENGER_MOLECULES
KEGG_LEISHMANIA_INFECTION
REACTOME_ETHANOL_OXIDATION
REACTOME_MHC_CLASS_II_ANTIGEN_PRESENTATION
REACTOME_INTERFERON_GAMMA_SIGNALING
REACTOME_FORMATION_OF_THE_BETA_CATENIN_TCF_TRANSACTIVATING_COMPLEX
REACTOME_COSTIMULATION_BY_THE_CD28_FAMILY
0.35 0.30 0.25 0.20 0.15 0.10 0.05 0.0
Proportion
0.5 1.0 1.5 2.0 2.5 3.0 3.5 4.0 4.5 5.0
-log10 adjusted P-value
HLA-C
HSPA1L
HSPA1A
HSPA1B
HLA-DQA2
HLA-DMA
HLA-DOA
HLA-DPA1
HLA-DPB1
HIST1H2BC
HIST1H2AC
HIST1H2BE
HIST1H2BF
HIST1H2BG
HIST1H2BI
C4A
HLA-DQB2
PVRL2
ADH5
ADH4
ADH1B
TERT
MEN1
B
-log 10 P-value
Up-regulated DEG
Down-regulated DEG
DEG (both side)
0.0 0.5 1.0 1.5 2.0 2.5
Liver
Brain_Nucleus_accumbens_basal_ganglia
Adipose_Subcutaneous
Brain_Caudate_basal_ganglia
Heart_Left_Ventricle
Breast_Mammary_Tissue
Brain_Anterior_cingulate_cortex_BA24
Ovary
Brain_Hippocampus
Lung
Thyroid
Brain_Cerebellar_Hemisphere
Brain_Putamen_basal_ganglia
Fallopian_Tube
Prostate
Nerve_Tibial
Uterus
Pancreas
Muscle_Skeletal
Brain_Amygdala
Brain_Hypothalamus
Brain_Cerebellum
Skin_Sun_Exposed_Lower_leg
Esophagus_Mucosa
Brain_Substantia_nigra
Kidney_Medulla
Brain_Spinal_cord_cervical_c-1
Testis
Brain_Frontal_Cortex_BA9
Vagina
Esophagus_Muscularis
Adipose_Visceral_Omentum
Cells_Cultured_fibroblasts
Heart_Atrial_Appendage
Whole_Blood
Colon_Sigmoid
Brain_Cortex
Esophagus_Gastroesophageal_Junction
Stomach
Skin_Not_Sun_Exposed_Suprapubic
Cervix_Endocervix
Adrenal_Gland
Spleen
Artery_Aorta
Cells_EBV-transformed_lymphocytes
Artery_Coronary
Minor_Salivary_Gland
Colon_Transverse
Small_Intestine_Terminal_Ileum
Artery_Tibial
Kidney_Cortex
Pituitary
Bladder
Cervix_Ectocervix

<u>Supplementary data</u>

Figure S1. Quantile-quantile (QQ) plots for all four meta-analysis results, annotated with genomic inflation scores (lambda GC) derived from linkage disequilibrium score regression (LDSC) analysis using ldsc.

Figure S2. Miami plot of single-ancestry meta-analysis results. Results from METAL European meta-analysis (8,216 cases and 1,558,609 controls in 7 cohorts, top) and METAL East Asian meta-analysis (7,355 cases and 629,454 controls in 4 cohorts, bottom) with genome-wide significant ($P < 5x10^{-8}$) loci in red and top suggestive loci in orange.

Figure S3. Region plots of suggestive associations. LocusZoom plots of genome-wide significant ($P < 5x10^{-8}$) loci (top) with forest plots demonstrating individual study effects (below) for: *GABPB2* (A-B), *STAT4* (C-D), and *MEN1* (E-F). BBJ, BioBank Japan; CKBB, China Kadoorie BioBank; KoGES; Korean BioBank; MVP, Million Veterans Programme; PLCO, Prostate, Lung, Colorectal and Ovarian (PLCO) Cancer Screening Trial; TPMI, Taiwan Precision Medicine Initiative; and UKBB, UK BioBank.

Figure S4. Pathway analysis results. MAGMA was used to perform gene set analysis using mapped genes from FUMA based on the METAL-all cohorts analysis. Significantly enriched canonical pathways (A) are based on Bonferroni-adjusted P-value <.05. Tissue enrichment was determined using GTEx v8 data based on up-regulation or down-regulation in the GWAS results (B).

Table S1. Summary of characteristics of included cohorts.

Table S2. LDSC genetic covariance intercept (gcov_int) between cohorts with EAS and within EUR ancestries with their 95% confidence intervals (CI).

Table S3. Previously reported significant/suggestive loci for HCC from any cause annotated with results from MR-MEGA meta-analysis from this study.

Table S4. Suggestive and significant loci associated with HCC from MR-MEGA and METAL meta-analysis models. CHR, chromosome; BP, base pair; NEA, non-effect allele; EA, effect allele. BETA(SE), allelic effect in log odds ratio from MR-MEGA analysis; BETA(SE)_METAL, allelic effect in log odds ratio from METAL (all ancestries) analysis;P(MR-MEGA): two-sided P value of association from MR-MEGA (chi-squared test with df = 4); P(METAL-all), two-sided P value of association from sample-size weighted analysis using METAL with all ancestries; P(METAL-EUR/EAS), two-sided P value of association from sample-size weighted analysis using METAL with European (EUR) or East Asian (EAS) ancestries; P(ANC-HET), P value for the two-sided ancestral heterogeneity test (chi-squared test with df = 3); P(RES-HET): P value for the two-sided residual heterogeneity test (chi-squared test with df = 3); EAF, effect allele frequency.

Table S5. Results from colocalisation analyses. Where: PP.H0.abf = Probability neither HCC or cirrhosis are associated; PP.H1.abf = Probability only HCC is associated; PP.H2.abf = Probability only cirrhosis is associated; PP.H3.abf = Probability both traits are associated, but with different causal variants; PP.H4.abf = Probability both traits are associated and share the same causal variant. nsnps = number of SNPs included in coloc analysis.

Table S6. Results from sensitivity analysis in cirrhotic controls: meta-analysis of Trepo et al. and Hassan et al. using METAL with sample size weighting, with focused analysis of previously identified significant and suggestive loci (from any previous analysis). P-values underwent Bonferroni adjustment for multiple tests.

Table S7. Fine-mapping results. Posterior probabilities were calculated from Bayes scores from MR-MEGA meta-analysis results for all significant and suggestive loci. Loci were filtered for those with fewer than 5 variants per 95% credible set. Ensembl Variant Effect

Predictor was used to annotate variants within the credible sets, keeping coding variants and those with high CADD scores. CADD, Combined Annotation Dependent Depletion score; CHR, chromosome; nGWAS-SNPs, number of SNPs with the locus that met significant/suggestive thresholds.

Table S8. Pathway analysis using MAGMA based on mapped genes from FUMA analysis. P-values were Bonferroni adjusted and only significantly enriched pathways are shown.

Table S9. Transcriptome-wide analysis (TWAS) results. FUSION was used to perform TWAS using normal liver tissue and hepatocellular carcinoma (HCC) eQTLs. TWAS p-values were adjusted for multiple testing with Benjamini–Hochberg correction with the alpha of 0.05.

Table S10. Regulome-wide analysis (RWAS) results. FUSION was used to perform RWAS using chromatin accessibility profiles for hepatocellular carcinoma (HCC) eQTLs. RWAS p-values were adjusted for multiple testing with Benjamini–Hochberg correction with the alpha of 0.05.

Table S11. Summary of linkage disequilibrium score regression (LDSC) correlation analyses between hepatocellular carcinoma (HCC) and related traits. BMI, body mass index; HBV, hepatitis B virus; HCV; hepatitis C virus; MASLD, metabolic dysfunction-associated steatotic liver disease; T2DM, type 2 diabetes mellitus; upper GI (gastrointestinal) cancer, oesophageal and gastric cancer.

**SupTab1**	Summary of characteristics of included cohorts.
**SupTab2**	LDSC genetic covariance intercept (gcov_int) between cohorts with EAS and within EUR ancestries with their 95% confidence intervals (CI).
**SupTab3**	Previously reported significant/suggestive loci for HCC from any cause annotated with results from MR-MEGA meta-analysis from this study.
**SupTab4**	Suggestive and significant loci associated with HCC from MR-MEGA and METAL meta-analysis models. CHR, chromosome; BP, base pair; NEA, non-effect allele; EA, effect allele. BETA(SE), allelic effect in log odds ratio from MR-MEGA analysis; BETA(SE)_METAL, allelic effect in log odds ratio from METAL (all ancestries) analysis;P(MR-MEGA): two-sided P value of association from MR-MEGA (chi-squared test with df = 4); P(METAL-all), two-sided P value of association from sample-size weighted analysis using METAL with all ancestries; P(METAL-EUR/EAS), two-sided P value of association from sample-size weighted analysis using METAL with European (EUR) or East Asian (EAS) ancestries; P(ANC-HET), P value for the two-sided ancestral heterogeneity test (chi-squared test with df = 3); P(RES-HET): P value for the two-sided residual heterogeneity test (chi-squared test with df = 3); EAF, effect allele frequency.
**SupTab5**	Results from colocalisation analyses. Where: PP.H0.abf = Probability neither HCC or cirrhosis are associated; PP.H1.abf = Probability only HCC is associated; PP.H2.abf = Probability only cirrhosis is associated; PP.H3.abf = Probability both traits are associated, but with different causal variants; PP.H4.abf = Probability both traits are associated and share the same causal variant. nsnps = number of SNPs included in coloc analysis.
**SupTab6**	Results from sensitivity analysis in cirrhotic controls: meta-analysis of Trepo et al. and Hassan et al. using METAL with sample size weighting, with focused analysis of previously identified significant and suggestive loci (from any previous analysis). P-values underwent Bonferroni adjustment for multiple tests.
**SupTab7**	Fine-mapping results. Posterior probabilities were calculated from Bayes scores from MR-MEGA meta-analysis results for all significant and suggestive loci. Loci were filtered for those with fewer than 5 variants per 95% credible set. Ensembl Variant Effect Predictor was used to annotate variants within the credible sets, keeping coding variants and those with high CADD scores. CADD, Combined Annotation Dependent Depletion score; CHR, chromosome; nGWAS-SNPs, number of SNPs with the locus that met significant/suggestive thresholds.
**SupTab8**	Pathway analysis using MAGMA based on mapped genes from FUMA analysis. P-values were Bonferroni adjusted and only significantly enriched pathways are shown.

**SupTab9**	Transcriptome-wide analysis (TWAS) results. FUSION was used to perform TWAS using normal liver tissue and hepatocellular carcinoma (HCC) eQTLs. TWAS p-values were adjusted for multiple testing with Benjamini–Hochberg correction with the alpha of 0.05.
**SupTab10**	Regulome-wide analysis (RWAS) results. FUSION was used to perform RWAS using chromatin accessibility profiles for hepatocellular carcinoma (HCC) eQTLs. RWAS p-values were adjusted for multiple testing with Benjamini–Hochberg correction with the alpha of 0.05.
**SupTab11**	Summary of linkage disequilibrium score regression (LDSC) correlation analyses between hepatocellular carcinoma (HCC) and related traits. BMI, body mass index; HBV, hepatitis B virus; HCV; hepatitis C virus; MASLD, metabolic dysfunction-associated steatotic liver disease; T2DM, type 2 diabetes mellitus; upper GI (gastrointestinal) cancer, oesophageal and gastric cancer.

Cohort	Ancestry	N Cases	N Control	HCC Definition
Million Veteran Program (African)	AFR	977	120,676	ICD10:C22.0
Million Veteran Program (Admixed American)	AMR	630	59,146	ICD10:C22.0
Biobank Japan	EAS	1866	195,745	ICD10:C22.0, Phecode: 155.1
China Kadoorie BioBank	EAS	665	75,355	ICD10:C22
Korea BioBank	EAS	46	72,252	ICD10:C22.0
Taiwan Precision Medicine Initiative	EAS	4778	286,102	ICD10:C22.0
deCODE	EUR	406	396,414	ICD10:C22.0
FinnGen	EUR	982	286,608	ICD10:C22.0
Hassan et al	EUR	1872	2,907	ICD10:C22.0
Intermountain	EUR	81	26,333	ICD10:C22.0
Million Veteran Program (European)	EUR	2852	447,587	ICD10:C22.0
Trepo et al	EUR	1649	2,391	Imaging or histology
UK Biobank	EUR	374	396,369	ICD10:C22.0
All Of Us	Mixed	368	291,385	ICD10:C22.0
PLCO Atlas	Mixed	151	56,413	ICD10:C22.0
	**Totals**	**17,697**	**2,715,683**	

Genotyping Platform	Reference
MVP 1.0 Custom Axiom Array	https://doi.org/10.1016/j.jclinepi.2015.09.016
MVP 1.0 Custom Axiom Array	https://doi.org/10.1016/j.jclinepi.2015.09.016
Illumina HumanOmniExpressExome v.1.0/v.1.2, HumanOmniExpress v.1.0 and Human Exome BeadChip v.1.0/v.1.1	https://doi.org/10.1016/j.je.2016.12.005
Custom-designed Affymetrix Axiom	https://doi.org/10.1016/j.xgen.2023.100361
KoreaChip Array	https://doi.org/10.1093/ije/dyv316
TPMv1, TPMv2, Axiom Genome-Wide TWB 2.0 Array	https://doi.org/10.1038/s41586-025-09680-x
Illumina Human Global Screening Array and Illumina Human Omni Screening Array	https://doi.org/10.1038/s41588-022-01199-5
Illumina chip arrays, Affymetrix chip arrays	https://doi.org/10.1038/s41586-022-05473-8
Illumina 1M Array	https://doi.org/10.1097/HEP.0000000000000800
Illumina Global screening array family	https://doi.org/10.1038/s41588-022-01199-5
MVP 1.0 Custom Axiom Array	https://doi.org/10.1016/j.jclinepi.2015.09.016
Illumina Human Global Screening Array	https://doi.org/10.1016/S1470-2045(21)00603-3
UK BiLEVE Axiom Array/UK Biobank Axiom Array	https://doi.org/10.1371/journal.pmed.1001779
Short-Read Whole Genome Sequencing (srWGS) using Illumina Kapa HyperPrep kit	https://doi.org/10.1056/NEJMsr1809937
OncoArray, Omni2.5 M, OmniExpress, and Illumina Global Screening Array	https://doi.org/10.1038/s41597-022-01921-2

Comparison	Ancestry	gcov_int (se)	95% CI
BBJ vs CKBB	EAS	0.0015 (0.0044)	[-0.0071 - 0.0101]
Hassan vs decode	EUR	-0.0068 (0.0038)	[-0.0142 - 0.0006]
Hassan vs Finngen	EUR	0.0001 (0.0045)	[-0.0087 - 0.0089]
Hassan vs intermountain	EUR	-0.0086 (0.0044)	[-0.0172 - 0.0000]
Hassan vs Trepo	EUR	-0.0029 (0.0050)	[-0.0127 - 0.0069]
Hassan vs UKBB	EUR	-0.0001 (0.0047)	[-0.0093 - 0.0091]
CKBB vs KoGES	EAS	-0.0038 (0.0042)	[-0.0120 - 0.0044]
CKBB vs TPMI	EAS	0.0038 (0.0043)	[-0.0046 - 0.0122]
decode vs Finngen	EUR	0.0032 (0.0047)	[-0.0060 - 0.0124]
decode vs intermountain	EUR	0.0054 (0.0043)	[-0.0030 - 0.0138]
decode vs Trepo	EUR	0.0004 (0.0047)	[-0.0088 - 0.0096]
decode vs UKBB	EUR	0.0018 (0.0045)	[-0.0070 - 0.0106]
Finngen vs intermountain	EUR	-0.0021 (0.0047)	[-0.0113 - 0.0071]
Finngen vs UKBB	EUR	0.0062 (0.0042)	[-0.0020 - 0.0144]
intermountain vs Trepo	EUR	0.0088 (0.0047)	[-0.0004 - 0.0180]
intermountain vs UKBB	EUR	-0.0011 (0.0046)	[-0.0101 - 0.0079]
TPMI vs BBJ	EAS	0.0034 (0.0048)	[-0.0060 - 0.0128]
Trepo vs UKBB	EUR	-0.0011 (0.0043)	[-0.0095 - 0.0073]

Previously identified loci

rsID	chr	pos	Annotation	Aetiology	GWAS Significance (p)	Source
rs17401966	1	10325413	KIF1B	HBV	1.70E-18	https://doi.org/10.1080/00365521.2016.1245778
rs12057222	1	16182039	EPHA2	All cause	6.10E-09	https://doi.org/10.1101/2025.09.16.25335186
rs187199523	1	194027489	Intergenic	All cause	2.17E-09	https://doi.org/10.3390/ijms242216417
rs2642442	1	220800221	MTARC1	All cause	3.98E-09	https://doi.org/10.1016/j.jhepr.2025.101485
rs708113	1	228005052	WNT3A-WNT	Alcohol	1.11E-08	https://doi.org/10.1016/S1470-2045(21)00603-3
rs7574865	2	191099907	STAT4	HBV	2.48E-10	https://doi.org/10.1038/ng.2483
rs9842969	3	39558686	MOBP	Non viral	1.36E-09	https://doi.org/10.1097/HEP.0000000000000800
rs7628416	3	126376169	KLF15	All cause	1.77E-08	https://doi.org/10.1016/j.jhepr.2025.101485
rs2120243	3	157429779	VEPH1	HBV	2.00E-06	https://doi.org/10.3892/ol.2015.3958
rs140233124	4	6834347	KIAA0232	All cause	4.34E-10	https://doi.org/10.3390/ijms242216417
rs72613567	4	87310241	HSD17B13	All cause	4.40E-17	https://doi.org/10.1101/2025.09.16.25335186
rs144285059	4	136895711	Intergenic	All cause	1.99E-08	https://doi.org/10.3390/ijms242216417
rs17047200	4	166008836	TLL1	HCV	2.66E-08	https://doi.org/10.1053/j.gastro.2017.01.041
rs148610742	4	186288789	F11	All cause	1.43E-08	https://doi.org/10.3390/ijms242216417
rs10069690	5	1279675	TERT	All cause, alc	1.70E-16	https://doi.org/10.1101/2025.09.16.25335186
rs79220007	6	26098246	HFE	All cause	2.10E-14	https://doi.org/10.1101/2025.09.16.25335186
rs2523961	6	29971803	HLA	HBV	7.50E-12	https://doi.org/10.1038/s41598-018-26217-7
rs1110446	6	30103160	HLA	HBV	1.66E-13	https://doi.org/10.1038/s41598-018-26217-7
rs3094137	6	30233096	HLA	HBV	7.09E-09	https://doi.org/10.1038/s41598-018-26217-7
rs2596542	6	31398818	HLA	HCV	4.21E-13	https://doi.org/10.1038/ng.809
rs41270883	6	32660820	HLA	All cause	1.70E-19	https://doi.org/10.1101/2025.09.16.25335186
rs9275224	6	32692101	HLA	HCV	1.45E-12	https://doi.org/10.1097/HEP.0000000000000800
rs9275319	6	32698518	HLA	HBV	2.72E-17	https://doi.org/10.1038/ng.2483
rs10272859	7	90689160	CDK14	HBV	9.46E-10	https://doi.org/10.1158/1078-0432.CCR-17-2537
rs118180127	8	8513430	Intergenic	All cause	7.30E-09	https://doi.org/10.3390/ijms242216417
rs77236434	8	13261659	DLC1	HBV	2.64E-07	https://doi.org/10.1002/cncr.30851
rs117719091	10	6227313	PFKFB3	All cause	3.02E-09	https://doi.org/10.3390/ijms242216417
rs17155112	10	14357172	FRMD4A	All cause	3.02E-08	https://doi.org/10.3390/ijms242216417

rs60299329	11	2370014	CD81	All cause	5.00E-08	https://doi.org/10.1101/2025.09.16.25335186
rs77404202	11	20117743	NAV2	All cause	2.46E-08	https://doi.org/10.3390/ijms242216417
rs6591900	11	80654502	TENM4	All cause	4.70E-08	https://doi.org/10.1101/2025.09.16.25335186
rs1350171	11	86933347	FZD4	HBV	6.48E-06	https://doi.org/10.3892/ol.2015.3958
rs144225287	12	3568611	PRMT8	All cause	5.27E-10	https://doi.org/10.3390/ijms242216417
rs150098717	12	8198462	Intergenic	All cause	1.81E-08	https://doi.org/10.3390/ijms242216417
rs28929474	14	94378610	SERPINA1	All cause	5.50E-07	https://doi.org/10.1016/j.jhepr.2025.101485
rs74333160	14	101184577	Intergenic	All cause	1.42E-12	https://doi.org/10.3390/ijms242216417
rs80115676	17	17375355	Intergenic	All cause	6.34E-09	https://doi.org/10.3390/ijms242216417
rs58542926	19	19268740	TM6SF2	All cause	4.80E-08	https://doi.org/10.1101/2025.09.16.25335186
rs10401969	19	19296909	TM6SF2	Non viral	4.87E-14	https://doi.org/10.1097/HEP.0000000000000800
rs58489806	19	19346108	TM6SF2	Non viral	4.87E-14	https://doi.org/10.1097/HEP.0000000000000800
rs3794991	19	19499787	TM6SF2	Non viral	3.99E-13	https://doi.org/10.1097/HEP.0000000000000800
rs12971396	19	39247226	IFNL3	All cause	3.40E-10	https://doi.org/10.1016/j.jhepr.2025.101485
rs8099917	19	39252525	IFNL4	All cause	1.50E-18	https://doi.org/10.1101/2025.09.16.25335186
rs769449	19	44906745	APOE	All cause	6.30E-12	https://doi.org/10.1101/2025.09.16.25335186
rs429358	19	44908684	APOE	All cause	1.07E-13	https://doi.org/10.1016/j.jhepr.2025.101485
rs6140450	20	7873320	Intergenic	All cause	2.79E-08	https://doi.org/10.3390/ijms242216417
rs455804	21	29773850	GRIK1	HBV	1.86E-08	https://doi.org/10.1371/journal.pgen.1002791
rs1012068	22	31869917	DEPDC5	HCV	1.27E-13	https://doi.org/10.1038/ng.876
rs738409	22	43928847	PNPLA3	All cause	9.60E-63	https://doi.org/10.1101/2025.09.16.25335186
rs2294922	22	43983685	PNPLA3	Non viral	1.21E-31	https://doi.org/10.1097/HEP.0000000000000800
rs2401514	22	43998139	PNPLA3	Non viral	4.13E-18	https://doi.org/10.1097/HEP.0000000000000800

Results from MR-MEGA in this analysis

Effects	beta	se	P	P.value_ance	P.value_resi	EAF	NEA	EA
+-+-++-+---+	-0.00341	0.0171159	0.46124385	0.34289148	0.27842619	0.278829	A	G
++++++++++	0.104957	0.0182888	5.96E-11	0.12047746	0.18157149	0.7621	A	G
-++++++?++-	0.0538465	0.0258894	0.00121832	0.02175536	0.00489535	0.767813	C	T
---+---+--+-?	0.0103104	0.0296963	0.36049545	0.27139314	2.80E-06	0.622817	T	A
+---+++++++	0.0255438	0.0154272	0.0016944	0.07269833	0.46719578	0.717848	T	G
+--+---??-+-?	-0.104514	0.0775399	0.00106022	0.00042025	0.00013063	0.914125	T	C
++?------+--?	-0.0370865	0.0242722	0.31908781	0.45620233	0.02517679	0.688538	C	G
---+?++++-++	0.00875553	0.0143891	0.72311807	0.66530893	0.2757202	0.613105	A	C
+---------+-?-	-0.0331549	0.0179064	0.04408185	0.04876944	0.6270844	0.133588	A	T
+?--+????+??	0.372868	10.0804	0.88559534	0.81112672	0.10635316	0.0179575	C	T
----++--------	-0.152259	0.0235852	2.50E-20	0.05073456	0.00804304	0.249459	C	T
-+++-?+?++-	-0.111172	1.01072	3.30E-13	0.03720415	0.00115007	0.0418883	T	C
+-++++----++	0.00465715	0.0225173	0.85648216	0.80006626	0.25923622	0.136397	G	A
++++-+----++	0.00249424	0.0167849	0.88197755	0.79026163	0.50697681	0.189792	C	T
--+-------+-?-	-0.0242842	0.0164891	0.58895637	0.67525624	0.76953129	0.891487	A	G
+-++++--+--+	0.00062571	0.0153109	0.24099901	0.15545381	0.33246199	0.333828	C	T
-???--?+----?	-0.14479	0.0279862	6.60E-15	1.58E-05	0.27933232	0.169354	C	T
--+-?+++----?	-0.0824356	0.0312784	1.07E-08	0.00012049	8.15E-06	0.578676	A	G
--++-+++--++	-0.0319304	0.0163876	0.00018339	0.00703081	0.65562589	0.166991	A	G
++?-++--+-+-	-0.017219	0.0129946	0.65805601	0.68402626	0.55755272	0.524265	G	C
+-+++--++++-	0.0237763	0.0253356	0.8630791	0.82881623	0.58123886	0.0705429	C	T
+?----????++	-0.757787	0.489429	0.6529875	0.52665929	0.68752429	0.018962	G	A

++++-+--+--+	-0.0303123	0.0765025	1.19E-05	0.0016776	0.28534647	0.0570187	C	T
++++---+-+++	0.0880785	0.0260599	5.55E-05	0.01838863	0.19297409	0.228186	G	A
++-+----+--+?	-0.0005115	0.0164281	0.32738374	0.21887463	0.54524015	0.720145	C	T
?+++-?+?+++-	1.46189	2.3992	1.96E-06	0.08306122	0.49212737	0.0156569	C	T
?---??+???--?	-0.0473682	0.0624105	0.91276496	0.96202095	0.27457353	0.0618326	T	G
++?++++++++	0.331958	0.0383738	1.58E-48	1.12E-14	0.01215293	0.0693607	C	T
+++++-+++++	0.308569	0.0305583	7.86E-48	3.88E-20	0.03635041	0.0915803	T	C
+++++-?++++	0.269057	0.0237299	5.25E-39	4.97E-20	0.22686858	0.130051	C	T
++++++++++-	0.285793	0.0276968	6.19E-43	2.96E-16	0.14290472	0.0814716	C	T
++++?-+-+++-	0.146685	0.0285945	3.39E-15	0.00223053	0.01172487	0.139551	C	G
++++--+-+++-	0.154843	0.0261553	7.65E-17	0.00236008	0.01967066	0.128717	T	G
---------------	-0.185737	0.0168784	1.63E-15	0.00041038	0.90734458	0.093042	G	A
-----+---------	-0.166119	0.0149405	4.93E-16	9.75E-05	0.84161531	0.127383	T	C
-+-++++++++	0.0197745	0.016152	0.12432883	0.09169622	0.46343768	0.737282	A	C
--+------+-+?	-0.015587	0.0161397	0.37423795	0.3488229	0.32883985	0.273242	T	G
+++++-+++++	0.330701	0.0204487	8.71E-123	2.07E-33	0.02556719	0.31229	C	G
+++++-++++-	0.271131	0.0182103	2.62E-85	3.51E-31	0.12050854	0.321135	G	C
+++++-+++++	0.220418	0.0153727	1.95E-48	2.34E-14	0.47085525	0.292532	T	A

rsID	Gene	chr	start	end	LeadVarID
rs924204	EPHA2	1	16119854	16405015	1:16187431:A>G
rs77018356	GABPB2_CTS	1	150567705	151256476	1:151162485:G>C
rs780094	GCKR	2	27375230	27617773	2:27518370:T>C
rs4089	HSD17B13	4	86936370	87392102	4:87300193:C>G
rs6822348	ADH5	4	98961982	99710112	4:99132743:A>T
rs112850336	MTTP	4	98961982	99710112	4:99492512:C>T
rs10069690	TERT	5	1236575	1334754	5:1279675:C>T
rs1800562	HFE	6	25488355	28526448	6:26092913:G>A
rs1811359	HLA	6	30258068	35395959	6:33084605:G>C
rs1561929	MYC	8	128522288	128557753	8:128555127:C>T
rs77052733	MAP3K9	14	70708492	71287739	14:70819179:C>T
rs8107974	TM6SF2	19	18599018	20495333	19:19277691:A>T
rs12983038	IFNL3	19	39238839	39255172	19:39250484:G>A
rs429358	APOE	19	44834661	45120209	19:44908684:T>C
rs738408	PNPLA3	22	43845535	44015164	22:43928850:C>T
rs192102882	DPYD	1	96749676	98696559	1:97730862:G>A
rs642748	BRINP3	1	190559861	190899390	1:190582288:A>G
rs574727703	LEMD1	1	205412769	205412769	1:205412769:G>T
rs2358112	KCNH1	1	210729573	210736926	1:210736550:A>C
rs2642438	MTARC1	1	220796686	220800419	1:220796686:A>G
rs1489153	CRIM1	2	36088389	36128149	2:36128149:C>A
rs139981561	MTA3	2	42480214	42623720	2:42500162:G>A
rs16822975	PDE1A	2	182204531	182256007	2:182256007:C>G
rs7601754	STAT4	2	191051800	191105604	2:191075725:G>A
rs143031340	ANO10	3	43398976	43398976	3:43398976:T>C
rs7619951	LMLN	3	198003629	198109909	3:198078784:G>T
rs181279855	Inter_4	4	64145645	65128154	4:64778403:A>T
rs150997702	UGT2B15	4	68455461	69414805	4:69105792:A>C
rs560414912	TENM3	4	182014188	182014188	4:182014188:C>T
rs549655936	Inter_5_26	5	25239794	26836818	5:26117041:C>T
rs17237359	TTC33	5	40502876	40615982	5:40585446:G>A
rs567431306	Inter_5_109	5	109666349	109995642	5:109911833:C>T
rs115916940	ADAM19	5	157458466	157727319	5:157548355:T>C
rs262883	DOCK2	5	169723174	169723174	5:169723174:T>C
rs117914127	ECI2DT	6	4225007	4260125	6:4246952:T>A
rs116019395	Inter_6_15	6	15539317	15714997	6:15714997:G>T
rs565801566	ROS1	6	117319757	117794087	6:117352023:A>G
rs578159714	Inter_7	7	743269	1008812	7:795344:T>A
rs192291282	EEPD1	7	36116075	37185558	7:36296283:G>A
rs10215153	MET	7	116716022	116857877	7:116759077:G>A
rs7787465	TCAF1	7	143997519	144042910	7:143999157:G>A
rs138704172	SGCZ	8	14120606	14120606	8:14120606:A>C
rs527402601	SNTG1	8	49699490	51575717	8:50111199:G>A
rs559235319	HNF4G	8	75135344	75351134	8:75351134:G>C
rs113176322	CDKN2A	9	21698855	21931559	9:21860825:C>G

rs77595131	LINC03142	9	23005093	23024740	9:23020160:G>A
rs12342193	NCS1	9	130149054	130176696	9:130150220:G>A
rs142566255	PHYH	10	13275785	13276619	10:13275881:C>G
rs140294052	Inter_10	10	34002674	34027686	10:34017127:A>G
rs16934377	KCNMA1	10	77208882	77265261	10:77227406:A>G
rs190142270	ATAD1	10	87840499	88083250	10:87840499:G>A
rs41282902	DOCK1	10	127419693	127419693	10:127419693:G>A
rs77207831	STK32C	10	132272410	132280521	10:132272410:G>A
rs59128789	CD81	11	2370014	2399846	11:2370530:T>C
rs2301564	MEN1	11	64800220	64813774	11:64800220:G>C
rs575579699	C11orf24	11	67541801	69158039	11:68256958:C>T
rs78313558	Inter_12_88	12	87621330	87719983	12:87621330:G>A
rs11106620	Inter_12_92	12	92528655	92554915	12:92546287:G>A
rs577598638	TMEM119	12	108350393	108589439	12:108589439:C>A
rs6491934	Inter_13	13	105118421	105158793	13:105140171:T>C
rs112635299	SERPINA1	14	94206394	94378610	14:94371805:G>T
rs74376607	NUSAP1	15	41276192	41392227	15:41333452:A>T
rs2355900	Inter_16_49	16	49002461	49060260	16:49060260:C>A
rs188950677	Inter_16_61	16	60686100	61978859	16:61072880:C>T
rs182904311	Inter_16_63	16	63095480	63095480	16:63095480:T>G
rs8071333	RAP1GAP2	17	2912430	2912430	17:2912430:G>T
rs11658199	ANKFN1	17	55995887	56037844	17:56002983:A>G
rs2034945	WBP2	17	75850233	76028630	17:75867265:T>A
rs35332090	ENDOV	17	80361810	80430106	17:80363258:G>C
rs35332090	RNF213	17	80361810	80430106	17:80363258:G>C
rs1968334	TPGS2_KIAA1	18	36730286	37261099	18:36763671:T>A
rs148194981	RAB11B	19	8359282	8402690	19:8386830:G>C
rs8122913	Inter_20	20	57786040	57786040	20:57786040:T>G
rs559612992	GATA5	20	62510610	62510610	20:62510610:G>C
rs569227697	KCNJ6	21	37597951	38395133	21:37818137:G>A
rs2542030	GRAMD4	22	46633557	46671371	22:46671371:G>C

Effects	Beta(SE)_MR	P(MR-MEGA	Beta(SE)_ME	P(METAL-all)	P(METAL-EU
+++++-++++-	0.10 (0.02)	9.20e-13	0.006 (0.000	7.12e-13	1.36e-13
?++++?-+-+-+	2.90 (3.88)	4.91e-6	0.011 (0.002	1.02e-6	3.84e-8
-----++-----?-	-0.08 (0.01)	2.22e-7	-0.005 (0.000	8.69e-9	8.46e-7
----+-------?-	-0.15 (0.02)	1.78e-20	-0.007 (0.000	2.82e-17	5.74e-18
-----?------??	-0.12 (0.10)	3.12e-7	-0.006 (0.001	4.44e-8	3.43e-8
-----+-+---+?-	-0.17 (0.04)	2.57e-6	-0.006 (0.001	2.01e-8	6.83e-5
----++--------	-0.15 (0.02)	2.50e-20	-0.009 (0.001	1.84e-21	6.40e-16
?+++-?+?++-	0.12 (2.89)	1.74e-13	0.019 (0.002	5.96e-13	5.58e-14
+---++----++?	0.04 (0.01)	4.38e-26	0.006 (0.000	8.29e-17	1.18e-8
?++++?+++++	-0.71 (1.70)	1.00e-6	0.010 (0.001	2.96e-8	8.77e-8
-+++--++++-+	0.14 (0.04)	1.54e-6	0.007 (0.001	2.87e-8	1.22e-6
+++++-+++++	0.31 (0.03)	1.95e-48	0.018 (0.001	1.09e-36	2.00e-47
++++--+?+++	0.15 (0.03)	1.91e-17	0.010 (0.001	3.41e-15	6.51e-8
-----+--------	-0.17 (0.01)	4.93e-16	-0.010 (0.001	3.87e-14	1.13e-16
+++++-+++++	0.33 (0.02)	1.06e-121	0.019 (0.000	2.35e-96	4.41e-96
?++-+???-+-+	-24.17 (11.03	4.17e-7	0.079 (0.018	1.41e-5	5.80e-8
++--++--++-+	0.03 (0.01)	2.88e-6	0.004 (0.000	4.37e-5	2.37e-4
?+-++?+++-+-	2.59 (3.81)	4.39e-3	0.118 (0.024	1.62e-6	9.20e-4
-??++?-?-++-?	-0.00 (0.02)	2.21e-6	0.084 (0.019	2.71e-5	3.85e-3
-++++++++++	0.07 (0.02)	7.01e-5	0.004 (0.001	2.57e-4	5.98e-7
+++++-++++-	0.00 (0.02)	6.23e-7	-0.013 (0.003	7.26e-5	6.71e-3
?+-+-----+++?	0.08 (0.09)	4.83e-4	0.006 (0.002	1.55e-2	5.55e-5
+++?--???+++	0.52 (0.14)	2.31e-5	0.010 (0.002	2.79e-7	6.15e-4
--+++-++++--	-0.01 (0.01)	7.85e-6	-0.003 (0.001	5.43e-3	3.11e-4
?-++-?-?++++	6.93 (7.75)	6.32e-3	0.036 (0.007	1.66e-6	6.53e-4
?---+-+---++?	-0.08 (0.04)	6.49e-4	-0.005 (0.001	6.91e-4	2.19e-6
?-??++-+++--?	0.07 (0.10)	8.66e-4	0.116 (0.024	2.59e-6	1.08e-5
?-++-????++-?	-182.71 (75.3	1.35e-4	0.027 (0.005	1.45e-6	1.28e-5
?+---?+?++++	5.99 (3.47)	4.81e-4	0.107 (0.021	8.69e-7	9.02e-4
?--+-???-+?-?	-74.90 (4.43)	5.98e-4	0.101 (0.021	2.15e-6	1.47e-4
----++------?-	-0.23 (0.06)	3.35e-4	-0.012 (0.002	2.43e-6	5.38e-4
+-++-+------?	-0.03 (0.02)	2.15e-3	0.236 (0.050	2.52e-6	4.27e-4
+++++--+-+--	0.03 (0.01)	4.60e-4	0.071 (0.014	1.57e-6	1.65e-4
--+++?--+--+?	-0.13 (0.02)	2.89e-6	0.085 (0.024	5.30e-4	2.20e-3
?----????+-+?	-148.70 (52.2	2.41e-3	0.017 (0.003	1.00e-6	1.39e-3
?+?+-?+?-+++	-19.74 (9.08)	6.70e-4	0.068 (0.014	2.93e-6	1.26e-3
?+--+?+??--+?	72.03 (11.48)	9.55e-4	0.126 (0.026	2.08e-6	2.35e-4
?++++???+++	-10.91 (8.90)	2.07e-4	0.058 (0.014	4.43e-5	2.45e-6
-+--+------+?-	-0.00 (0.03)	8.69e-5	0.063 (0.018	5.34e-4	1.37e-6
++-+-+-++++-	0.07 (0.02)	9.80e-5	0.005 (0.001	1.01e-6	2.86e-4
------+-----?+	-0.06 (0.01)	2.27e-5	-0.003 (0.000	2.28e-3	1.21e-6
?--++?-----+?	0.31 (3.72)	8.78e-4	0.103 (0.021	2.14e-6	2.79e-4
??++-???++-+	-17.27 (30.97	2.30e-5	0.078 (0.018	1.67e-5	7.04e-7
??-+-???-++-?	-91.12 (32.02	3.94e-4	0.239 (0.047	6.03e-7	3.44e-5
+-++-+???---?	0.03 (0.09)	2.63e-4	0.007 (0.002	3.53e-3	3.98e-3

?--++?+++++-	3.70 (3.99)	1.28e-6	0.009 (0.0028	1.52e-3	2.23e-3
+-++-++++++	0.07 (0.04)	5.05e-5	0.007 (0.0016	3.62e-7	1.01e-3
-?-?--??---+?-	-0.38 (0.11)	2.65e-4	0.111 (0.0235	2.04e-6	1.18e-3
?+-++?-++++-	4.36 (2.85)	9.53e-4	0.089 (0.0183	1.08e-6	7.98e-5
++++-???--+-?	0.28 (0.16)	1.21e-4	0.016 (0.0067	1.63e-2	2.61e-3
?----?+-----??	-10.12 (8.55)	3.87e-4	-0.018 (0.003	2.49e-6	3.27e-6
?----???-+++?	-104.29 (25.7	3.35e-7	0.024 (0.0108	2.66e-2	6.34e-4
-?-?--???+--?-	-0.05 (0.10)	5.11e-4	-0.006 (0.001	1.01e-4	1.17e-4
++--++?-+--+	0.01 (0.08)	2.97e-6	-0.003 (0.000	6.83e-4	3.20e-4
--+-+-??----?-	-0.14 (0.11)	1.01e-4	-0.006 (0.001	2.25e-6	2.94e-3
?+-+-???+++-	13.34 (12.32)	2.25e-4	0.688 (0.1415	1.14e-6	1.14e-6
?++?+?+++++	2.41 (1.10)	2.45e-4	0.011 (0.0023	2.68e-6	4.68e-5
++++-++-----?	-0.02 (0.02)	2.63e-6	0.466 (0.1456	1.37e-3	2.71e-4
?+-+-????+--?	-28.24 (37.40	1.87e-5	0.055 (0.0162	7.02e-4	3.00e-6
+++++--++-+-	0.06 (0.02)	8.95e-6	0.004 (0.0009	1.40e-6	2.65e-4
?+++-?+?+++-	8.45 (3.89)	1.63e-7	0.024 (0.0050	1.11e-6	1.93e-7
+-+++-+++++	0.09 (0.02)	8.11e-4	0.006 (0.0014	2.74e-6	2.99e-3
+++++--+----?	0.01 (0.02)	1.48e-5	0.004 (0.0012	2.34e-3	1.01e-3
?++++???-++-	-41.23 (5.96)	2.17e-4	0.097 (0.0206	2.59e-6	2.59e-6
?-?+-++----+?	0.01 (0.02)	3.71e-3	0.031 (0.0065	1.76e-6	2.53e-3
++++-++++++	0.04 (0.03)	1.42e-6	-0.006 (0.001	2.86e-4	5.32e-3
+?-+++-?+++-	0.06 (0.02)	1.85e-4	0.005 (0.0010	1.42e-6	3.08e-3
?+??-++++++-	0.06 (0.07)	1.14e-4	0.004 (0.0013	2.36e-3	1.11e-6
----+++------+	-0.11 (0.03)	3.88e-6	-0.006 (0.001	1.23e-4	1.56e-6
----+++------+	-0.11 (0.03)	3.88e-6	-0.006 (0.001	1.23e-4	1.56e-6
?++-+?+?+++-	1.32 (1.56)	7.17e-7	0.010 (0.0021	1.38e-6	1.94e-5
?+-?+???+++-	8.71 (5.83)	2.02e-5	0.057 (0.0114	4.93e-7	2.93e-6
-????-??--++?	-0.05 (0.02)	1.94e-5	-0.003 (0.001	3.96e-2	2.36e-3
+++-?+---+?-?	-0.77 (0.13)	4.91e-3	0.164 (0.0332	7.72e-7	3.34e-4
+++-+-+++++	0.07 (0.01)	6.85e-8	0.195 (0.0408	1.83e-6	1.70e-5
++-+++-+-++-	0.02 (0.02)	6.34e-4	0.029 (0.0061	1.71e-6	2.73e-4

P(METAL-EAS)	P(ANC-HET)	P(RES-HET)	EAF	Significant
9.47e-3	5.43e-4	1.23e-1	0.65	Significant
2.06e-3	8.97e-2	8.76e-3	0.06	Significant
9.07e-3	2.22e-1	4.79e-1	0.57	Significant
4.10e-2	2.01e-9	1.24e-1	0.3	Significant
3.69e-5	9.90E-01	6.72E-01	0.76	Significant
4.77e-5	5.27E-02	5.65E-01	0.22	Significant
5.77e-5	5.07e-2	8.04e-3	0.25	Significant
9.83e-4	2.26e-2	9.51e-4	0.05	Significant
5.39e-29	1.70e-14	8.76e-1	0.46	Significant
4.28e-4	6.35e-1	5.48e-1	0.88	Significant
6.82e-5	2.97e-3	1.55e-1	0.07	Significant
1.63e-2	5.70e-20	1.74e-2	0.09	Significant
5.02e-7	3.83e-3	4.84e-2	0.13	Significant
3.16e-2	9.75e-5	8.42e-1	0.13	Significant
6.87e-13	1.42e-33	3.18e-2	0.31	Significant
3.17e-3	5.11e-2	4.53e-1	2.60e-03	Suggestive
1.28e-7	1.13e-3	8.15e-1	0.41	Suggestive
4.28e-3	2.49e-3	4.21e-1	0.04	Suggestive
5.06e-4	7.19e-7	2.02e-1	0.47	Suggestive
2.05e-2	1.07e-2	2.24e-2	0.77	Suggestive
4.69e-2	2.77e-7	3.73e-1	0.52	Suggestive
2.30e-6	8.85e-4	1.07e-1	0.03	Suggestive
6.99e-4	1.51e-2	2.11e-1	0.06	Suggestive
1.43e-7	7.00e-5	8.11e-1	0.8	Suggestive
1.66e-6	1.30e-2	1.45e-1	0.01	Suggestive
7.32e-3	1.07e-2	2.58e-1	0.12	Suggestive
7.75e-4	3.41e-4	7.82e-4	0.34	Suggestive
1.45e-6	1.70e-4	4.17e-1	2.64e-03	Suggestive
2.84e-3	6.28e-3	9.35e-1	6.78e-03	Suggestive
6.01e-4	6.19e-4	9.68e-1	1.12e-03	Suggestive
7.12e-3	2.88e-1	2.72e-1	0.03	Suggestive
4.45e-3	8.97e-4	3.63e-1	0.29	Suggestive
2.35e-3	2.10e-3	4.40e-1	0.33	Suggestive
4.59e-4	1.46e-6	7.19e-1	0.8	Suggestive
5.50e-6	2.87e-3	6.98e-1	1.24e-03	Suggestive
1.32e-4	2.70e-3	3.43e-1	0.01	Suggestive
2.85e-3	3.83e-4	7.43e-1	0.02	Suggestive
4.23e-4	2.70e-1	8.48e-1	2.05e-03	Suggestive
3.34e-4	3.20e-5	7.21e-1	0.18	Suggestive
6.38e-3	7.29e-1	4.14e-1	0.22	Suggestive
3.90e-2	3.51e-4	8.75e-1	0.47	Suggestive
1.62e-3	2.25e-3	6.09e-1	0.02	Suggestive
2.73e-3	6.24e-1	1.68e-1	8.33e-04	Suggestive
7.75e-3	3.77e-3	8.43e-1	9.91e-04	Suggestive
9.20e-7	2.08e-1	5.38e-1	0.04	Suggestive

1.39e-2	1.59e-3	7.81e-2	0.05	Suggestive
2.64e-7	2.26e-1	3.28e-1	0.08	Suggestive
5.00e-3	1.18e-4	9.83e-1	0.06	Suggestive
1.12e-3	2.59e-1	8.25e-2	0.11	Suggestive
1.02e-6	1.89e-2	5.05e-1	6.59e-03	Suggestive
4.79e-3	2.10e-1	8.96e-4	0.02	Suggestive
2.58e-2	2.09e-4	4.14e-1	3.42e-03	Suggestive
2.05e-6	9.79e-1	9.43e-1	0.09	Suggestive
2.08e-3	1.34e-5	3.34e-1	0.04	Suggestive
2.03e-5	7.07e-1	9.23e-2	0.16	Suggestive
4.30e-4	3.36e-2	6.28e-2	6.88e-03	Suggestive
2.64e-2	6.73e-1	8.72e-1	0.08	Suggestive
1.99e-2	8.70e-7	4.58e-1	0.27	Suggestive
1.30e-2	1.64e-2	6.49e-1	1.21e-03	Suggestive
9.89e-4	1.91e-2	1.08e-1	0.36	Suggestive
3.98e-4	3.64e-2	5.65e-1	0.02	Suggestive
2.53e-4	7.69e-1	5.26e-1	0.11	Suggestive
2.78e-6	1.47e-4	3.80e-1	0.16	Suggestive
1.33e-4	4.07e-3	9.58e-1	1.62e-03	Suggestive
1.76e-6	1.98e-2	9.13e-1	0.7	Suggestive
8.82e-4	4.42e-7	1.30e-1	0.37	Suggestive
1.38e-5	4.79e-1	5.24e-1	0.38	Suggestive
3.33e-3	7.90e-3	4.63e-1	0.18	Suggestive
1.68e-3	2.43e-2	1.81e-1	0.08	Suggestive
1.68e-3	2.43e-2	1.81e-1	0.08	Suggestive
2.84e-2	7.69e-2	7.17e-1	0.16	Suggestive
2.76e-3	1.82e-1	7.59e-1	3.97e-03	Suggestive
1.41e-6	3.98e-5	3.95e-1	0.61	Suggestive
1.87e-3	3.46e-3	8.88e-1	0.4	Suggestive
2.79e-3	5.78e-4	4.26e-1	0.47	Suggestive
1.29e-3	1.10e-3	6.66e-1	0.26	Suggestive

Locus	nsnps	Neither<br>PP.H0.abf	HCC only<br>PP.H1.abf	Cirrhosis only<br>PP.H2.abf	Both, different variant<br>PP.H3.abf
ADH5-MTTP	1273	0.002753832	0.05104029	0.002435572	0.044242039
APOE	1102	2.75E-14	6.31E-08	4.85E-08	0.110288481
EPHA2	1088	4.49E-06	0.967498008	1.44E-07	0.030988825
GABPB2	890	0.531878188	0.422812684	0.011163196	0.008848804
GCKR	823	0.051686818	0.930932401	8.56E-04	0.015422597
HFE	1200	3.41E-12	2.93E-07	1.83E-08	5.69E-04
HLA	2309	8.53E-14	0.004415572	5.55E-12	0.28659341
HSD17B13	1224	1.48E-32	1.79E-23	5.75E-11	0.068799143
IFNL3	1043	6.33E-08	0.241551952	4.33E-08	0.164755288
MAP3K9	1299	0.049936828	0.593740444	0.02747027	0.326614629
MYC	1358	0.121796312	0.719507677	0.004177845	0.024550515
PNPLA3	1062	0	3.56E-256	1.01E-91	0
TERT	1087	0.755948332	0.221082762	0.016994116	0.004969051
TM6SF2	835	1.66E-65	1.03E-39	3.92E-29	0.001430694
ADAM19	569	0.724062636	0.241192858	0.020029518	0.006663992
ANO10	479	0.984773796	0.005464484	0.009548693	5.28E-05
ATAD1	535	0.978104694	0.007273299	0.014216677	1.05E-04
BRINP3	711	0.963072125	0.016086334	0.020213404	3.37E-04
CDKN2A	703	0.942329729	0.041947703	0.014717411	6.55E-04
COL22A1	682	0.980643989	0.008384462	0.010730539	9.16E-05
DOCK1	833	0.979854307	0.006535945	0.013139804	8.73E-05
DOCK2	648	0.982116173	0.007275837	0.010306383	7.61E-05
DPYD	523	0.982959241	0.006372886	0.010430064	6.75E-05
ENDOV	557	0.961173414	0.008144418	0.029874023	2.53E-04
GRAMD4	625	0.976865624	0.014004279	0.008627457	1.23E-04
HNF4G	709	0.975420665	0.009633953	0.01453151	1.43E-04
Inter_10	692	0.97331502	0.009714449	0.016392297	1.63E-04
Inter_12_88	567	0.976546995	0.012077354	0.011036013	1.36E-04
Inter_13	898	0.752519971	0.100404619	0.127395162	0.016994954
Inter_16_49	567	0.976653406	0.011646483	0.011334417	1.35E-04
Inter_16_61	691	0.974580934	0.007208492	0.017780141	1.31E-04
Inter_16_63	678	0.706879231	0.27633052	0.00809354	0.003158357
Inter_3	718	0.971535934	0.007129226	0.020898912	1.53E-04
Inter_4	673	0.544052099	0.420453501	0.014054468	0.010850963
Inter_5_109	707	0.979242053	0.007023654	0.01344016	9.62E-05
Inter_5_26	795	0.972807792	0.006795745	0.020039615	1.40E-04
Inter_5_86	657	0.968439993	0.019771872	0.01121789	2.29E-04
Inter_7	606	0.332952747	0.012550247	0.623202597	0.023483048
KCNH1	626	0.968360872	0.006831054	0.024277131	1.71E-04
KCNJ6	565	0.978090561	0.011506115	0.010059466	1.18E-04
KCNMA1	585	0.982961187	0.007063107	0.009650306	6.91E-05
LEMD1	592	0.980998869	0.005071201	0.013629444	7.02E-05
LINC01811	673	0.665959178	0.3067688	0.015287753	0.007037235
LINC03142	1060	0.838865548	0.137634827	0.017726703	0.002905598
MTARC1	819	5.39E-15	6.86E-17	0.779847407	0.00972305
MYL2	467	1.65E-07	1.66E-09	0.966147295	0.009693584

NCS1	531	0.380344889	0.609155601	0.003387518	0.005423717
NUSAP1	435	0.700486443	0.286751328	0.007326422	0.002996707
PDPR2P	444	0.987923653	0.005579111	0.006325977	3.56E-05
PLSCR4	750	0.974553478	0.005435982	0.019685784	1.10E-04
PSMD11	407	0.979498483	0.006496722	0.013669368	9.04E-05
RAP1GAP2	664	0.980477654	0.008232058	0.010971405	9.19E-05
RBMS3	740	0.960063738	0.005959955	0.03352214	2.08E-04
RNF213	557	0.961173414	0.008144418	0.029874023	2.53E-04
SERPINA1	682	6.12E-07	5.06E-09	0.473011187	0.003382514
SGCZ	1235	0.950564337	0.022897479	0.024745304	5.95E-04
SNTG1	497	0.983232862	0.00751558	0.008974367	6.84E-05
TENM3	689	0.977475568	0.007381987	0.014857681	1.12E-04
TMEM119	483	0.986626431	0.004007576	0.009193255	3.72E-05
TSHB	588	0.968442259	0.02133924	0.009823856	2.16E-04
TTC33	615	0.810549804	0.174726954	0.010679532	0.0023004
UGT2B15	954	0.610407829	0.369209563	0.008070201	0.00487388
WBP2	440	0.965881047	0.023959079	0.009630513	2.39E-04
ZNF438	701	0.981094857	0.006996287	0.011633793	8.28E-05

Both, shared variant
PP.H4.abf
0.899528266
0.889711407
0.001508537
0.025297128
0.001101836
0.999430369
0.708991018
0.931200857
0.593692653
0.002237828
0.129967651
1
0.001005739
0.998569306
0.008050996
1.60E-04
3.00E-04
2.91E-04
3.50E-04
1.49E-04
3.83E-04
2.25E-04
1.70E-04
5.56E-04
3.79E-04
2.71E-04
4.15E-04
2.03E-04
0.002685293
2.31E-04
2.99E-04
0.005538351
2.83E-04
0.01058897
1.98E-04
2.17E-04
3.42E-04
0.007811361
3.60E-04
2.26E-04
2.56E-04
2.30E-04
0.004947034
0.002867324
0.210429543
0.024158955

0.001688275
0.0024391
1.36E-04
2.15E-04
2.45E-04
2.27E-04
2.46E-04
5.56E-04
0.523605682
0.001198006
2.09E-04
1.73E-04
1.36E-04
1.78E-04
0.001743309
0.007438527
2.91E-04
1.92E-04

rsID	Gene	uniqID	Beta(SE)	P(METAL-all)	Category	DIRECTION
rs738408	PNPLA3	22:43928850	0.019 (0.000	2.35E-96	Significant	++
rs8107974	TM6SF2	19:19277691	0.018 (0.001	1.09E-36	Significant	--
rs10069690	TERT	5:1279675:C	-0.009 (0.001	1.84E-21	Significant	--
rs924204	EPHA2	1:16187431:	0.006 (0.000	7.12E-13	Significant	--
rs4089	HSD17B13	4:87300193:	-0.007 (0.000	2.82E-17	Significant	++
rs429358	APOE	19:44908684	-0.010 (0.001	3.87E-14	Significant	++
rs77018356	GABPB2_CTS	1:151162485	0.011 (0.002	1.02E-06	Significant	++
rs77052733	MAP3K9	14:70819179	0.007 (0.001	2.87E-08	Significant	++
rs780094	GCKR	2:27518370:	-0.005 (0.000	8.69E-09	Significant	++
rs1139490	ADH1B	4:99123990:	-0.006 (0.001	2.01E-08	Significant	--
rs1561929	MYC	8:128555127	0.010 (0.001	2.96E-08	Significant	++
rs12983038	IFNL3	19:39250484	0.010 (0.001	3.41E-15	Significant	-+
rs1811359	HLA	6:33084605:	0.006 (0.000	8.29E-17	Significant	--
rs1800562	HFE	6:26092913:	0.019 (0.002	5.96E-13	Significant	NA
rs10215153	MET	7:116759077	0.005 (0.001	1.01E-06	Suggestive	++
rs6491934	Inter_13	13:10514017	0.004 (0.000	1.40E-06	Suggestive	--
rs190142270	ATAD1	10:87840499	-0.018 (0.003	2.49E-06	Suggestive	--
rs11658199	ANKFN1	17:56002983	0.005 (0.001	1.42E-06	Suggestive	++
rs2034945	WBP2	17:75867265	0.004 (0.001	0.00236	Suggestive	++
rs7787465	TCAF1	7:143999157	-0.003 (0.000	0.00228	Suggestive	--
rs17237359	TTC33	5:40585446:	-0.012 (0.002	2.43E-06	Suggestive	--
rs78313558	Inter_12_88	12:87621330	0.011 (0.002	2.68E-06	Suggestive	++
rs7619951	LMLN	3:198078784	-0.005 (0.001	0.000691	Suggestive	--
rs8071333	RAP1GAP2	17:2912430:	-0.006 (0.001	0.000286	Suggestive	++
rs139981561	MTA3	2:42500162:	0.006 (0.002	0.0155	Suggestive	+-
rs35332090	ENDOV	17:80363258	-0.006 (0.001	0.000123	Suggestive	--
rs35332090	RNF213	17:80363258	-0.006 (0.001	0.000123	Suggestive	--
rs1968334	TPGS2_KIAA1	18:36763671	0.010 (0.002	1.38E-06	Suggestive	+-
rs12342193	NCS1	9:130150220	0.007 (0.001	3.62E-07	Suggestive	-+
rs262883	DOCK2	5:169723174	0.085 (0.024	0.00053	Suggestive	++
rs2355900	Inter_16_49	16:49060260	0.004 (0.001	0.00234	Suggestive	++
rs77595131	LINC03142	9:23020160:	0.009 (0.002	0.00152	Suggestive	-+
rs642748	BRINP3	1:190582288	0.004 (0.000	4.37E-05	Suggestive	-+
rs2642438	MTARC1	1:220796686	0.004 (0.001	0.000257	Suggestive	--
rs74376607	NUSAP1	15:41333452	0.006 (0.001	2.74E-06	Suggestive	+-
rs1489153	CRIM1	2:36128149:	-0.013 (0.003	7.26E-05	Suggestive	++
rs11106620	Inter_12_92	12:92546287	0.466 (0.145	0.00137	Suggestive	+-
rs7601754	STAT4	2:191075725	-0.003 (0.001	0.00543	Suggestive	-+
rs59128789	CD81	11:2370530:	-0.003 (0.000	0.000683	Suggestive	-+
rs16822975	PDE1A	2:182256007	0.010 (0.002	2.79E-07	Suggestive	NA
rs148194981	RAB11B	19:8386830:	0.057 (0.011	4.93E-07	Suggestive	NA
rs559235319	HNF4G	8:75351134:	0.239 (0.047	6.03E-07	Suggestive	NA
rs559612992	GATA5	20:62510610	0.164 (0.033	7.72E-07	Suggestive	NA
rs560414912	TENM3	4:182014188	0.107 (0.021	8.69E-07	Suggestive	NA
rs117914127	ECI2DT	6:4246952:T	0.017 (0.003	1.00E-06	Suggestive	NA
rs140294052	Inter_10	10:34017127	0.089 (0.018	1.08E-06	Suggestive	NA
rs112635299	SERPINA1	14:94371805	0.024 (0.005	1.11E-06	Suggestive	NA

rs575579699	C11orf24	11:68256958	0.688 (0.141	1.14E-06	Suggestive	NA
rs150997702	UGT2B15	4:69105792:/	0.027 (0.005	1.45E-06	Suggestive	NA
rs115916940	ADAM19	5:157548355	0.071 (0.014	1.57E-06	Suggestive	NA
rs574727703	LEMD1	1:205412769	0.118 (0.024	1.62E-06	Suggestive	NA
rs143031340	ANO10	3:43398976:	0.036 (0.007	1.66E-06	Suggestive	NA
rs2542030	GRAMD4	22:46671371	0.029 (0.006	1.71E-06	Suggestive	NA
rs182904311	Inter_16_63	16:63095480	0.031 (0.006	1.76E-06	Suggestive	NA
rs569227697	KCNJ6	21:37818137	0.195 (0.040	1.83E-06	Suggestive	NA
rs142566255	PHYH	10:13275881	0.111 (0.023	2.04E-06	Suggestive	NA
rs565801566	ROS1	6:117352023	0.126 (0.026	2.08E-06	Suggestive	NA
rs138704172	SGCZ	8:14120606:/	0.103 (0.021	2.14E-06	Suggestive	NA
rs549655936	Inter_5_26	5:26117041:(	0.101 (0.021	2.15E-06	Suggestive	NA
rs2301564	MEN1	11:64800220	-0.006 (0.001	2.25E-06	Suggestive	NA
rs567431306	Inter_5_109	5:109911833	0.236 (0.050	2.52E-06	Suggestive	NA
rs188950677	Inter_16_61	16:61072880	0.097 (0.020	2.59E-06	Suggestive	NA
rs181279855	Inter_4	4:64778403:/	0.116 (0.024	2.59E-06	Suggestive	NA
rs116019395	Inter_6_15	6:15714997:(	0.068 (0.014	2.93E-06	Suggestive	NA
rs192102882	DPYD	1:97730862:(	0.079 (0.018	1.41E-05	Suggestive	NA
rs527402601	SNTG1	8:50111199:(	0.078 (0.018	1.67E-05	Suggestive	NA
rs2358112	KCNH1	1:210736550	0.084 (0.019	2.71E-05	Suggestive	NA
rs578159714	Inter_7	7:795344:T>/	0.058 (0.014	4.43E-05	Suggestive	NA
rs77207831	STK32C	10:13227241	-0.006 (0.001	0.000101	Suggestive	NA
rs192291282	EEPD1	7:36296283:(	0.063 (0.018	0.000534	Suggestive	NA
rs577598638	TMEM119	12:10858943	0.055 (0.016	0.000702	Suggestive	NA
rs113176322	CDKN2A	9:21860825:(	0.007 (0.002	0.00353	Suggestive	NA
rs16934377	KCNMA1	10:77227406	0.016 (0.006	0.0163	Suggestive	NA
rs41282902	DOCK1	10:12741969	0.024 (0.010	0.0266	Suggestive	NA
rs8122913	Inter_20	20:57786040	-0.003 (0.001	0.0396	Suggestive	NA

**Beta(SE)-cirrhosis**	**P(Cirrhosis)**	**P_adj(cirrhos**
0.03 (0)	2.89E-26	3.76E-25
0.036 (0.00439)	2.18E-16	2.84E-15
-0.016 (0.00282)	2.02E-08	2.63E-07
0.011 (0.00249)	5.99E-06	7.78E-05
-0.012 (0.00277)	1.36E-05	0.00017628
-0.014 (0.0035)	7.63E-05	0.0009919
0.018 (0.00465)	9.25E-05	0.00120237
0.019 (0.00609)	0.00171	0.02223
-0.007 (0.0025)	0.008326	0.108238
-0.007 (0.00265)	0.008984	0.116792
0.007 (0.00384)	0.08408	1
0.004 (0.003)	0.2422	1
-0.002 (0.00269)	0.4705	1
NA	NA	NA
0.01 (0.003)	0.0002727	0.0068175
0.01 (0.003)	0.0003254	0.008135
-0.026 (0.008)	0.0004869	0.0121725
-0.008 (0.00256)	0.001095	0.027375
0.009 (0.003)	0.003857	0.096425
-0.006 (0.003)	0.01745	0.43625
-0.018 (0.008)	0.03032	0.758
0.009 (0.004)	0.0397	0.9925
-0.007 (0.004)	0.08353	1
0.006 (0.004)	0.1906	1
-0.008 (0.006)	0.1942	1
-0.004 (0.004)	0.2801	1
-0.004 (0.004)	0.2801	1
0.003 (0.00336)	0.308	1
-0.007 (0.007)	0.3369	1
-0.003 (0.003)	0.3417	1
0.002 (0.003)	0.4201	1
-0.003 (0.006)	0.5791	1
0.001 (0.002)	0.5846	1
0.001 (0.003)	0.6018	1
0.002 (0.006)	0.7564	1
0.001 (0.003)	0.7578	1
0.001 (0.003)	0.7845	1
0.001 (0.003)	0.7939	1
0.002 (0.012)	0.8559	1
NA	NA	NA
NA	NA	NA
NA	NA	NA
NA	NA	NA
NA	NA	NA
NA	NA	NA
NA	NA	NA
NA	NA	NA

NA	NA	NA
NA	NA	NA
NA	NA	NA
NA	NA	NA
NA	NA	NA
NA	NA	NA
NA	NA	NA
NA	NA	NA
NA	NA	NA
NA	NA	NA
NA	NA	NA
NA	NA	NA
NA	NA	NA
NA	NA	NA
NA	NA	NA
NA	NA	NA
NA	NA	NA
NA	NA	NA
NA	NA	NA
NA	NA	NA
NA	NA	NA
NA	NA	NA
NA	NA	NA
NA	NA	NA
NA	NA	NA
NA	NA	NA
NA	NA	NA
NA	NA	NA

sis)

ChrStartEnd	nGWASSNPs	VarInfo	Gene	Variant	Consequenc	CADD
22:43927717	273	22:43928847	PNPLA3	p.Ile148Met	missense_vai	13.4
19:18952750	508	19:19268740	TM6SF2	p.Glu167Lys	missense_vai	24.6
19:44883210	40	19:44908684	APOE	p.Cys130Arg	missense_vai	16.6
6:25526091:2	27	6:26092913:/	HFE	p.Cys176Tyr	missense_vai	27
2:27375230:2	8	2:27508073:(	GCKR	p.Leu446Pro	missense_vai	10.99
10:12741969	1	10:12741969	DOCK1	p.Glu1553Ly:	missense_vai	23.6
19:8359282:8	3	19:8369239:(	ANGPTL4	p.Glu190Gln	missense_vai	19.01
1:220796686	5	1:220796686	MTARC1	p.Thr165Pro	missense_vai	18.3
6:31354452:3	2	6:31355519:(	HLA-B	p.Gly231=	synonymous_variant	
14:94206394	4	14:94378610	SERPINA1	p.Glu366Gln	missense_vai	24.2
11:64800220	14	11:64805130	MEN1	p.Asp418Glu	missense_vai	19.25
10:87840499	2	10:87840499	ATAD1		intron_variar	4.102
6:33574701:3	21	6:33579653:/	BAK1		intron_variar	5.253
5:169723174	1	5:169723174	DOCK2		intron_variar	4.506
1:97677942:9	2	1:97751120:/	DPYD		intron_variar	0.088
1:16138178:1	126	1:16178825:(	EPHA2-AS1		intron_variar	14.56
18:36748997	4	18:36767873	FHOD3		intron_variar	5.251
6:31779044:3	2073	6:32660695:(	HLA-DQB1		intron_variar	5.696
19:39238839	34	19:39246873	IFNL3		upstream_ge	0.129
10:77208882	3	10:77227406	KCNMA1		intron_variar	1.516
14:70708492	44	14:70778889	MAP3K9		intron_variar	6.826
2:42480214:4	22	2:42538776:(	MTA3		intron_variar	3.049
2:182204531	3	2:182256007	PDE1A		intron_variar	11.19
10:13275785	3	10:13276060	PHYH		downstream_	1.669
17:2912430:2	1	17:2912430:7	RAP1GAP2		intron_variar	3.926
2:191051800	30	2:191103694	STAT4		intron_variar	3.936
10:13227241	2	10:13227241	STK32C		intron_variar	0.417
5:1279675:12	9	5:1279675:T:	TERT		intron_variar	7.843

Category	GeneSet	N_genes	N_overlap	p	adjP	genes
GWAScatalog	Bipolar II disc	16	10	3.06E-20	1.36E-16	NCAN:HAPLN
GWAScatalog	Nonalcoholic	21	10	1.32E-18	2.93E-15	GCKR:C2orf1
Positional_ge	chr6p21	281	19	6.68E-17	2.00E-14	HLA-C:VWA7
GWAScatalog	Chronic hepa	11	7	1.51E-14	2.23E-11	HLA-C:NOTC
GO_cc	GOCC_MHC_	20	8	2.48E-14	2.51E-11	HFE:HLA-C:H
GWAScatalog	Aspartate am	317	17	1.53E-13	1.69E-10	EPHA2:ARHG
GWAScatalog	Autism spect	469	19	7.28E-13	6.45E-10	HFE:PGBD1:I
GWAScatalog	Nonalcoholic	5	5	2.29E-12	1.69E-09	GCKR:HSD17
GWAScatalog	Bipolar I diso	76	10	2.89E-12	1.83E-09	NCAN:HAPLN
GO_cc	GOCC_MHC_	12	6	9.38E-12	4.73E-09	HLA-DQA2:H
GWAScatalog	Cirrhosis (alc	13	6	1.74E-11	9.60E-09	HSD17B13:SI
GO_bp	GOBP_PEPTII	11	6	4.71E-12	3.65E-08	HLA-DQA2:H
GWAScatalog	Triglycerides	149	11	1.35E-10	6.64E-08	GCKR:C2orf1
GWAScatalog	Percent liver	9	5	2.84E-10	1.26E-07	C2orf16:TM6
GWAScatalog	Cirrhosis (mu	4	4	4.98E-10	1.84E-07	HSD17B13:SI
GWAScatalog	Liver fat cont	4	4	4.98E-10	1.84E-07	GCKR:TM6SF
GO_bp	GOBP_PEPTII	15	6	5.02E-11	1.95E-07	HLA-DQA2:H
GWAScatalog	Low density li	223	12	7.30E-10	2.49E-07	FBXO42:GCK
GWAScatalog	Alanine amin	281	13	8.67E-10	2.74E-07	EPHA2:ARHG
KEGG	KEGG_ANTIG	71	8	1.72E-09	3.20E-07	HLA-C:HSPA
KEGG	KEGG_ALLOG	32	6	8.52E-09	5.28E-07	HLA-C:HLA-D
KEGG	KEGG_GRAFT	32	6	8.52E-09	5.28E-07	HLA-C:HLA-D
KEGG	KEGG_SYSTE	90	8	1.16E-08	5.41E-07	HIST1H2BC:I
GWAScatalog	LDL choleste	147	10	2.18E-09	6.43E-07	GCKR:TMPRS
GO_mf	GOMF_MHC_	21	6	5.32E-10	9.43E-07	HLA-DQA2:H
KEGG	KEGG_TYPE_I	38	6	2.54E-08	9.43E-07	HLA-C:HLA-D
GWAScatalog	Asthma and r	81	8	5.00E-09	1.38E-06	PGBD1:HLA-C
GO_bp	GOBP_ANTIG	65	8	8.35E-10	2.16E-06	HFE:HLA-C:H
GWAScatalog	Lung cancer	170	10	8.90E-09	2.32E-06	ADH1B:TERT:
KEGG	KEGG_AUTOI	47	6	9.53E-08	2.95E-06	HLA-C:HLA-D
KEGG	KEGG_ASTHN	25	5	1.13E-07	3.00E-06	HLA-DQA2:H
GWAScatalog	Ulcerative cc	357	13	1.54E-08	3.80E-06	HLA-C:VWA7:
GWAScatalog	Hepatitis B	7	4	1.73E-08	4.02E-06	HLA-C:HLA-D
GWAScatalog	Total cholest	184	10	1.90E-08	4.20E-06	GCKR:TMPRS
GO_bp	GOBP_ANTIG	26	6	2.22E-09	4.29E-06	HLA-DQA2:H
GO_mf	GOMF_MHC_	30	6	5.63E-09	4.38E-06	HLA-DQA2:H
GO_mf	GOMF_MHC_	6	4	7.42E-09	4.38E-06	HLA-DQA2:H
GWAScatalog	Cholesterol	99	8	2.48E-08	5.23E-06	GCKR:HFE:H
Canonical_Pa	KEGG_ANTIG	71	8	1.72E-09	5.31E-06	HLA-C:HSPA
Positional_ge	chr19p13	547	15	4.82E-08	7.23E-06	ANGPTL4:ME
Canonical_Pa	KEGG_ALLOG	32	6	8.52E-09	8.77E-06	HLA-C:HLA-D
Canonical_Pa	KEGG_GRAFT	32	6	8.52E-09	8.77E-06	HLA-C:HLA-D
Canonical_Pa	KEGG_SYSTE	90	8	1.16E-08	8.99E-06	HIST1H2BC:I
GWAScatalog	Bipolar disor	473	14	5.55E-08	1.12E-05	C2orf16:ZNF
Curated_gen	KEGG_ANTIG	71	8	1.72E-09	1.12E-05	HLA-C:HSPA
GO_bp	GOBP_ANTIG	32	6	8.52E-09	1.32E-05	HLA-DQA2:H
Canonical_Pa	KEGG_TYPE_I	38	6	2.54E-08	1.57E-05	HLA-C:HLA-D

KEGG	KEGG_VIRAL_	65	6	6.84E-07	1.59E-05	HLA-C:HLA-D
GO_bp	GOBP_ANTIG	34	6	1.25E-08	1.62E-05	HLA-DQA2:H
GWAScatalo	Type 1 diabet	24	5	9.05E-08	1.74E-05	HLA-C:NOTC
Curated_gen	KEGG_GRAFT	32	6	8.52E-09	1.84E-05	HLA-C:HLA-D
Curated_gen	KEGG_ALLOG	32	6	8.52E-09	1.84E-05	HLA-C:HLA-D
Curated_gen	KEGG_SYSTE	90	8	1.16E-08	1.89E-05	HIST1H2BC:I
GWAScatalo	Cerebral amy	3	3	1.07E-07	1.90E-05	TOMM40:APC
GWAScatalo	Cognitive imp	3	3	1.07E-07	1.90E-05	TOMM40:APC
GO_cc	GOCC_LUME	22	5	5.65E-08	1.90E-05	HLA-C:HLA-D
GWAScatalo	Fasting trigly	11	4	1.60E-07	2.73E-05	GCKR:SUGP1
Curated_gen	KEGG_TYPE_I	38	6	2.54E-08	3.29E-05	HLA-C:HLA-D
KEGG	KEGG_INTEST	42	5	1.69E-06	3.50E-05	HLA-DQA2:H
GO_bp	GOBP_ANTIG	103	8	3.39E-08	3.76E-05	HFE:HLA-C:H
GWAScatalo	Postprandial	12	4	2.40E-07	3.93E-05	GCKR:SUGP1
GWAScatalo	Liver enzyme	134	8	2.63E-07	4.16E-05	ARHGEF19:PI
KEGG	KEGG_CELL_	125	7	2.35E-06	4.37E-05	HLA-C:HLA-D
Canonical_P	KEGG_AUTOI	47	6	9.53E-08	4.91E-05	HLA-C:HLA-D
Canonical_P	KEGG_ASTHN	25	5	1.13E-07	4.98E-05	HLA-DQA2:H
GO_cc	GOCC_LUME	28	5	2.06E-07	5.20E-05	HLA-C:HLA-D
GO_bp	GOBP_ANTIG	43	6	5.49E-08	5.32E-05	HLA-DQA2:H
Canonical_P	WP_ALLOGRA	84	7	1.57E-07	6.05E-05	HLA-C:C4A:H
GWAScatalo	Serum metab	323	11	4.19E-07	6.10E-05	GCKR:TMPRS
GWAScatalo	Cingulate cor	4	3	4.28E-07	6.10E-05	TOMM40:APC
GWAScatalo	Response to t	4	3	4.28E-07	6.10E-05	MAU2:GATAD
GWAScatalo	Response to I	14	4	4.81E-07	6.65E-05	HLA-C:HLA-D
TF_targets	CIITA_TARGE	1027	20	6.56E-08	7.31E-05	RSG1:PRUNE
GWAScatalo	Asthma	772	16	7.36E-07	9.87E-05	HLA-C:VWA7:
Curated_gen	KEGG_AUTOI	47	6	9.53E-08	0.00010315	HLA-C:HLA-D
Curated_gen	KEGG_ASTHN	25	5	1.13E-07	0.00010462	HLA-DQA2:H
Wikipathway	WP_ALLOGRA	84	7	1.57E-07	0.00011476	HLA-C:C4A:H
GWAScatalo	LDL choleste	216	9	9.61E-07	0.00012506	ARHGEF19:G
Curated_gen	WP_ALLOGRA	84	7	1.57E-07	0.00012711	HLA-C:C4A:H
GWAScatalo	Pulmonary ar	5	3	1.07E-06	0.00013408	PDE1A:HLA-D
GWAScatalo	Inflammatory	605	14	1.09E-06	0.00013408	GCKR:HLA-C
Canonical_P	KEGG_VIRAL_	65	6	6.84E-07	0.00023482	HLA-C:HLA-D
GO_bp	GOBP_B_CEI	135	8	2.79E-07	0.00024022	C4A:HLA-DQ
GWAScatalo	Alcohol cons	6	3	2.12E-06	0.00024728	GCKR:ADH5:
GWAScatalo	Waist Circun	6	3	2.12E-06	0.00024728	GCKR:C2orf1
KEGG	KEGG_LEISHI	65	5	1.51E-05	0.0002548	HLA-DQA2:H
GWAScatalo	Platelet cour	557	13	2.50E-06	0.00028383	GCKR:HSD17
GWAScatalo	Lipid traits	21	4	2.80E-06	0.00031002	GCKR:TOMM
GWAScatalo	Age-related r	48	5	3.33E-06	0.0003595	TNXB:ATF6B:I
GWAScatalo	C-reactive pr	22	4	3.41E-06	0.00035955	GCKR:TOMM
GWAScatalo	Alzheimer's d	7	3	3.70E-06	0.00036415	TOMM40:APC
GWAScatalo	Cerebrospina	7	3	3.70E-06	0.00036415	TOMM40:APC
GWAScatalo	Alzheimer's d	7	3	3.70E-06	0.00036415	TOMM40:APC
GWAScatalo	Sarcoidosis (	23	4	4.12E-06	0.00039594	TNXB:NOTCH
Canonical_P	REACTOME_I	18	4	1.45E-06	0.00044765	HLA-DQA2:H

Canonical_P	KEGG_INTES	42	5	1.69E-06	0.00047552	HLA-DQA2:H
Curated_gen	KEGG_VIRAL_	65	6	6.84E-07	0.00048678	HLA-C:HLA-D
Curated_gen	GAURNIER_P	66	6	7.49E-07	0.00048678	HLA-C:HLA-D
GWAScatalo	Total triglyce	8	3	5.90E-06	0.00053321	GCKR:ANGPT
GWAScatalo	Liver fibrosis	8	3	5.90E-06	0.00053321	HFE:TM6SF2:
GWAScatalo	Severe aplast	8	3	5.90E-06	0.00053321	HLA-DQA2:H
GO_cc	GOCC_COPI	83	6	2.92E-06	0.00058855	HLA-C:HLA-D
Canonical_P	KEGG_CELL_	125	7	2.35E-06	0.0005894	HLA-C:HLA-D
Canonical_P	WP_EBOLA_V	126	7	2.48E-06	0.0005894	HLA-C:HLA-D
GO_bp	GOBP_IMMU	68	6	8.96E-07	0.00068988	HLA-DQA2:H
GO_bp	GOBP_LYMPI	281	10	9.79E-07	0.00068988	HFE:HLA-C:C
GWAScatalo	Serum metab	58	5	8.58E-06	0.00075962	GCKR:PVRL2:
GWAScatalo	Oral cavity ca	9	3	8.83E-06	0.00076578	C2orf16:ZNF
GWAScatalo	Schizophreni	627	13	9.02E-06	0.00076772	HFE:TNXB:NC
GWAScatalo	Triglyceride l	534	12	9.24E-06	0.00077183	GCKR:C2orf1
GWAScatalo	Plateletcrit	446	11	9.46E-06	0.00077508	GCKR:ADH5:
GWAScatalo	Type 2 diabet	541	12	1.05E-05	0.00084753	BNIPL:GCKR:
Curated_gen	REACTOME_I	18	4	1.45E-06	0.00085539	HLA-DQA2:H
GWAScatalo	Medication u	61	5	1.10E-05	0.00087065	GCKR:TMPRS
GO_bp	GOBP_ADAPT	292	10	1.39E-06	0.00089488	HFE:HLA-C:C
Wikipathway	WP_EBOLA_V	126	7	2.48E-06	0.0009088	HLA-C:HLA-D
Curated_gen	KEGG_INTES	42	5	1.69E-06	0.00091623	HLA-DQA2:H
GO_cc	GOCC_ER_TO	53	5	5.48E-06	0.00092122	HLA-C:HLA-D
GO_mf	GOMF_ALCO	6	3	2.12E-06	0.00094072	ADH5:ADH4:
GWAScatalo	Persistent he	10	3	1.26E-05	0.00095859	HLA-DQB2:H
GWAScatalo	Carotid intim	10	3	1.26E-05	0.00095859	TOMM40:APC
GWAScatalo	Cerebrospina	31	4	1.42E-05	0.00106515	CDH8:TOMM
Curated_gen	KEGG_CELL_	125	7	2.35E-06	0.0011504	HLA-C:HLA-D
Curated_gen	WP_EBOLA_V	126	7	2.48E-06	0.0011504	HLA-C:HLA-D
GWAScatalo	Reticulocyte	388	10	1.70E-05	0.00122874	GCKR:ADH1E
GWAScatalo	Alcohol use d	11	3	1.72E-05	0.00122874	GCKR:ADH4:
GWAScatalo	HIV-1 control	11	3	1.72E-05	0.00122874	HLA-C:TNXB:
GWAScatalo	Liver enzyme	173	7	1.99E-05	0.00139908	EPHA2:ARHG
GWAScatalo	Ideal cardiov	2	2	2.28E-05	0.00140586	APOE:APOC1
GWAScatalo	Hippocampa	2	2	2.28E-05	0.00140586	TOMM40:APC
GWAScatalo	Serum type 1	2	2	2.28E-05	0.00140586	TOMM40:APC
GWAScatalo	Cognitive dec	2	2	2.28E-05	0.00140586	TOMM40:APC
GWAScatalo	Hepatitis B (v	2	2	2.28E-05	0.00140586	HLA-DPA1:HL
GWAScatalo	Verbal memo	2	2	2.28E-05	0.00140586	TOMM40:APC
GWAScatalo	Verbal memo	2	2	2.28E-05	0.00140586	TOMM40:APC
GWAScatalo	Lipoprotein-a	12	3	2.29E-05	0.00140586	GCKR:APOE:A
GWAScatalo	Cerebrospina	12	3	2.29E-05	0.00140586	TOMM40:APC
Cell_type_sig	DURANTE_AD	76	6	1.74E-06	0.00144031	HLA-DQA2:H
GO_mf	GOMF_PEPTI	24	4	4.92E-06	0.00174401	HFE:HLA-C:H
GWAScatalo	Cerebrospina	13	3	2.96E-05	0.00179631	TOMM40:APC
GWAScatalo	HDL choleste	510	11	3.27E-05	0.00195567	TMPRSS11E:K
GO_cc	GOCC_CLATI	65	5	1.51E-05	0.00217207	HLA-DQA2:H
GWAScatalo	Alzheimer's d	14	3	3.76E-05	0.00218834	TOMM40:APC

GWAScatalo	Cerebrospina	14	3	3.76E-05	0.00218834	TOMM40:APC
Canonical_P	REACTOME_(	29	4	1.08E-05	0.00238355	HLA-DQA2:H
Reactome	REACTOME_I	18	4	1.45E-06	0.00239616	HLA-DQA2:H
GWAScatalo	HDL choleste	196	7	4.43E-05	0.00254572	TMPRSS11E:/
Chemical_ar	GAURNIER_P	66	6	7.49E-07	0.00255192	HLA-C:HLA-D
GO_mf	GOMF_ALCO	9	3	8.83E-06	0.00260659	ADH5:ADH4:
Canonical_P	KEGG_LEISH	65	5	1.51E-05	0.00310419	HLA-DQA2:H
GWAScatalo	Apolipoprote	203	7	5.53E-05	0.00313693	ARHGEF19:G
GWAScatalo	Logical memo	16	3	5.74E-05	0.00313693	TOMM40:APC
GWAScatalo	C-reactive pr	16	3	5.74E-05	0.00313693	GCKR:HLA-C
GWAScatalo	Alzheimer's d	16	3	5.74E-05	0.00313693	TOMM40:APC
Canonical_P	REACTOME_I	11	3	1.72E-05	0.00332487	ADH5:ADH4:
GWAScatalo	Apolipoprote	367	9	6.74E-05	0.00345468	GCKR:TMPRS
GWAScatalo	Nonalcoholic	3	2	6.83E-05	0.00345468	PNPLA3:SAMI
GWAScatalo	Relative prot	3	2	6.83E-05	0.00345468	GCKR:ADH1E
GWAScatalo	Esophageal c	3	2	6.83E-05	0.00345468	ADH4:ADH1E
GWAScatalo	Apolipoprote	3	2	6.83E-05	0.00345468	APOE:APOC1
GWAScatalo	Cerebrospina	17	3	6.95E-05	0.00345468	TOMM40:APC
GWAScatalo	Palmitoleic a	17	3	6.95E-05	0.00345468	GCKR:C2orf1
GWAScatalo	Type 1 diabet	17	3	6.95E-05	0.00345468	HSPA1L:TNXE
Canonical_P	REACTOME_I	115	6	1.92E-05	0.00349496	HLA-DQA2:H
GO_bp	GOBP_ETHAN	8	3	5.90E-06	0.00352044	ADH5:ADH4:
GWAScatalo	Logical memo	18	3	8.31E-05	0.00395353	TOMM40:APC
GWAScatalo	Cerebrospina	18	3	8.31E-05	0.00395353	TOMM40:APC
GWAScatalo	C-reactive pr	18	3	8.31E-05	0.00395353	GCKR:NCAN:
GWAScatalo	Cardiovascul	18	3	8.31E-05	0.00395353	GCKR:HFE:TC
GWAScatalo	Squamous ce	93	5	8.53E-05	0.00401601	PGBD1:HLA-(
Curated_gen	REACTOME_(	29	4	1.08E-05	0.00467608	HLA-DQA2:H
GWAScatalo	High light sca	393	9	0.00011353	0.00525882	GCKR:ADH1E
GWAScatalo	C-reactive pr	20	3	0.00011528	0.00525882	GCKR:NCAN:
GWAScatalo	Quantitative	20	3	0.00011528	0.00525882	TOMM40:APC
Positional_ge	chr1q21	203	7	5.53E-05	0.00553271	PRUNE:BNIPI
GWAScatalo	Longevity	54	4	0.0001312	0.00579277	NOTCH4:TON
GWAScatalo	Antineutroph	21	3	0.00013402	0.00579277	HLA-DPA1:HL
GWAScatalo	Posterior cor	4	2	0.00013615	0.00579277	TOMM40:APC
GWAScatalo	Body mass in	4	2	0.00013615	0.00579277	GCKR:TOMM
GWAScatalo	Facial emotio	4	2	0.00013615	0.00579277	ROS1:GOPC
GWAScatalo	Hepatic lipid	4	2	0.00013615	0.00579277	SUGP1:PNPL/
GWAScatalo	Alcohol-relat	4	2	0.00013615	0.00579277	TM6SF2:PNPI
GWAScatalo	Metabolic sy	104	5	0.00014483	0.00610364	GCKR:ANGPT
Curated_gen	KEGG_LEISH	65	5	1.51E-05	0.00611702	HLA-DQA2:H
GO_cc	GOCC_CLATI	83	5	4.95E-05	0.00624206	HLA-DQA2:H
GWAScatalo	High light sca	409	9	0.0001533	0.00639544	GCKR:ADH1E
GWAScatalo	Phospholipid	22	3	0.00015465	0.00639544	GCKR:AGPAT
Curated_gen	REACTOME_I	11	3	1.72E-05	0.00657759	ADH5:ADH4:
Curated_gen	REACTOME_I	115	6	1.92E-05	0.00693808	HLA-DQA2:H
Positional_ge	chr4q23	19	3	9.83E-05	0.00737459	ADH5:ADH4:
GO_bp	GOBP_LEUKC	380	10	1.42E-05	0.00785695	HFE:HLA-C:C

Reactome	REACTOME_(	29	4	1.08E-05	0.00795075	HLA-DQA2:H
Reactome	REACTOME_I	11	3	1.72E-05	0.00795075	ADH5:ADH4:
Reactome	REACTOME_I	115	6	1.92E-05	0.00795075	HLA-DQA2:H
GWAScatalo	Gamma gluta	60	4	0.00019791	0.00810894	EPHA2:ARHG
GWAScatalo	Mental healt	24	3	0.00020184	0.00819402	TOMM40:APC
GWAScatalo	Resting heart	112	5	0.00020501	0.00824711	GCKR:MET:FI
GWAScatalo	Hepatitis C (:	5	2	0.0002262	0.00895061	HLA-DQA2:IF
GWAScatalo	Periodontal c	25	3	0.00022857	0.00895061	FHOD3:TPGS
GWAScatalo	Triglyceride l	25	3	0.00022857	0.00895061	GCKR:SUGP1
GO_cc	GOCC_COAT	293	8	8.32E-05	0.00933185	HLA-C:HLA-D
GWAScatalo	Triglyceride l	26	3	0.00025749	0.00999453	GCKR:SUGP1
Canonical_P	REACTOME_I	86	5	5.87E-05	0.01007617	HLA-C:HLA-D
GWAScatalo	Bitter alcoho	66	4	0.00028617	0.01101119	GCKR:ADH1E
GWAScatalo	Rosacea sym	121	5	0.00029368	0.01120297	KLHL8:HSD17
GO_bp	GOBP_IMMU	118	6	2.23E-05	0.01150251	HLA-DQA2:H
Cancer_mod	MODULE_14:	13	3	2.96E-05	0.01182526	HFE:HLA-C:H
Cancer_mod	MODULE_23	540	11	5.49E-05	0.01182526	SEMA6C:GCI
GWAScatalo	Alanine trans	28	3	0.00032219	0.01201876	KLHL8:SERPII
GWAScatalo	Carotid intim	28	3	0.00032219	0.01201876	TOMM40:APC
GWAScatalo	Factor VII act	6	2	0.00033824	0.01201876	GCKR:ADH4
GWAScatalo	White coat e	6	2	0.00033824	0.01201876	HLA-DPB1:CI
GWAScatalo	Gout vs asym	6	2	0.00033824	0.01201876	GCKR:ADH1E
GWAScatalo	Brain imaging	6	2	0.00033824	0.01201876	TOMM40:APC
GWAScatalo	Parental long	6	2	0.00033824	0.01201876	APOE:APOC1
GWAScatalo	EGFR mutati	6	2	0.00033824	0.01201876	TERT:HLA-DP
GWAScatalo	Alcohol cons	69	4	0.00033951	0.01201876	GCKR:ADH5:
GWAScatalo	Cerebrospina	29	3	0.00035812	0.01257667	TOMM40:APC
GWAScatalo	Cerebral amy	30	3	0.00039653	0.01381598	TOMM40:APC
KEGG	KEGG_FATTY_	40	3	0.00093205	0.01433846	ADH5:ADH4:
KEGG	KEGG_TYROS	41	3	0.00100215	0.01433846	ADH5:ADH4:
GWAScatalo	Body mass in	31	3	0.00043749	0.01500692	TOMM40:APC
GWAScatalo	Lipid metabo	31	3	0.00043749	0.01500692	GCKR:APOE:/
TF_targets	NFY_Q6_01	250	8	2.72E-05	0.01515635	HIST1H2BC:I
GO_cc	GOCC_BLOC	105	5	0.0001515	0.01528658	HSPA1L:HSP/
GWAScatalo	Pediatric nor	7	2	0.00047205	0.01606798	PNPLA3:SAMI
GWAScatalo	Automobile s	32	3	0.00048107	0.01612693	TNXB:ATF6B:
GWAScatalo	Body fat perc	32	3	0.00048107	0.01612693	TOMM40:APC
GWAScatalo	Lamb consun	33	3	0.00052735	0.0174143	TOMM40:APC
GWAScatalo	Hematologic	33	3	0.00052735	0.0174143	GCKR:PNPLA
GWAScatalo	Prostate can	297	7	0.00057069	0.01870609	TERT:C4A:TN:
Curated_gen	TARTE_PLASN	359	9	5.69E-05	0.01906157	ADH4:HSPA1
Curated_gen	REACTOME_I	86	5	5.87E-05	0.01906157	HLA-C:HLA-D
Reactome	REACTOME_I	86	5	5.87E-05	0.01941668	HLA-C:HLA-D
GO_cc	GOCC_ENDC	182	6	0.00024553	0.0202119	HLA-C:HLA-D
GO_cc	GOCC_VACU	436	9	0.00024661	0.0202119	HLA-DQA2:H
GO_cc	GOCC_COAT	184	6	0.00026041	0.0202119	HLA-C:HLA-D
GWAScatalo	Lymphoma	8	2	0.00062744	0.02041476	HLA-DQA2:HI
GO_cc	GOCC_PLASI	548	10	0.0002969	0.02131128	HFE:HLA-C:H

GO_cc	GOCC_CLATI	123	5	0.00031682	0.02131128	HLA-DQA2:H
GO_bp	GOBP_ADAPT	434	10	4.40E-05	0.0213362	HFE:HLA-C:C
GWAScatalo;	Cerebrospina	36	3	0.00068297	0.02205942	TOMM40:APC
GWAScatalo;	Red cell distr	404	8	0.00072524	0.02325503	GCKR:HFE:AI
GWAScatalo;	Hypertriglyce	37	3	0.00074066	0.0234101	GCKR:C2orf1
GWAScatalo;	Regular atter	37	3	0.00074066	0.0234101	ADH5:ADH4:A
GWAScatalo;	C-reactive pı	85	4	0.00075008	0.02353968	GCKR:SERPIN
Computatior	MODULE_143	13	3	2.96E-05	0.02354078	HFE:HLA-C:H
Computatior	MODULE_23	540	11	5.49E-05	0.02354078	SEMA6C:GCI
GWAScatalo;	Serum alkalir	312	7	0.00076303	0.0237776	GCKR:TMPRS
GWAScatalo;	Metabolically	9	2	0.00080418	0.02454143	GCKR:APOC1
GWAScatalo;	Body mass in	9	2	0.00080418	0.02454143	GCKR:TOMM
GWAScatalo;	Parental long	9	2	0.00080418	0.02454143	APOE:APOC1
GO_mf	GOMF_ANTIC	50	4	9.70E-05	0.02454333	HFE:HLA-C:H
GWAScatalo;	Hemoglobin l	316	7	0.00082213	0.0249174	HFE:PGBD1:I
Cancer_mod	MODULE_84	515	10	0.00018056	0.02594086	ADH1B:HLA-(
GWAScatalo;	Sj√∂gren's sy	39	3	0.00086514	0.02604236	HLA-DQA2:HI
GO_cc	GOCC_CLATI	201	6	0.00041759	0.02633447	HLA-DQA2:H
GWAScatalo;	Refractive er	1391	16	0.00089042	0.02662222	GCKR:HFE:P(
GWAScatalo;	Urate levels	236	6	0.00096805	0.02874913	GCKR:PDE1A
GWAScatalo;	Wegener's gr	10	2	0.00100209	0.02898375	HLA-DOA:HL/
GWAScatalo;	Alcohol consı	10	2	0.00100209	0.02898375	GCKR:ADH1E
GWAScatalo;	Thyrotoxic hy	10	2	0.00100209	0.02898375	HLA-DQA2:HI
GWAScatalo;	Hippocampa	41	3	0.00100215	0.02898375	TOMM40:APC
Cell_type_sig	FAN_EMBRY(	90	5	7.30E-05	0.03027991	HLA-C:HLA-D
GWAScatalo;	Hematocrit	330	7	0.00105808	0.03040252	GCKR:TERT:H
GWAScatalo;	Cerebrospina	42	3	0.00107551	0.03050714	TOMM40:APC
GWAScatalo;	Verbal declar	42	3	0.00107551	0.03050714	TOMM40:APC
Cell_type_sig	AIZARANI_LIV	99	5	0.00011478	0.03175657	ADH4:ADH1E
Canonical_P	REACTOME_I	60	4	0.00019791	0.03218692	TERT:HIST1H
GWAScatalo;	Alcohol consı	11	2	0.00122095	0.03397919	GCKR:ADH1E
GWAScatalo;	Fasting blooc	11	2	0.00122095	0.03397919	GCKR:C2orf1
GWAScatalo;	Liver volume	11	2	0.00122095	0.03397919	GCKR:MAU2
KEGG	KEGG_RETIN	57	3	0.00260263	0.03457781	ADH5:ADH4:
Canonical_P	REACTOME_(	62	4	0.00022476	0.03472523	HLA-DQA2:H
GWAScatalo;	Hippocampa	45	3	0.00131565	0.03638603	TOMM40:APC
GO_cc	GOCC_CELL_	832	12	0.00061721	0.03663316	EPHA2:ROBC
GWAScatalo;	Alzheimer's d	100	4	0.00137536	0.03780102	TNXB:TOMM4
GO_bp	GOBP_ETHAN	18	3	8.31E-05	0.03788465	ADH5:ADH4:
KEGG	KEGG_GLYC(	61	3	0.00315735	0.03820109	ADH5:ADH4:
KEGG	KEGG_AXON_	127	4	0.00328611	0.03820109	EPHA2:SEMA
GWAScatalo;	Prothrombin	12	2	0.00146056	0.0398824	GCKR:C2orf1
GWAScatalo;	Brain morphc	1049	13	0.00146911	0.0398824	ROBO2:HFE:I
KEGG	KEGG_METAE	65	3	0.00377948	0.04135192	ADH5:ADH4:
KEGG	KEGG_DRUG	67	3	0.00411657	0.0425379	ADH5:ADH4:
GWAScatalo;	Alzheimer's d	48	3	0.00158707	0.04256226	TOMM40:APC
GWAScatalo;	Type 2 diabet	48	3	0.00158707	0.04256226	GCKR:TM6SF
GO_cc	GOCC_MHC_	9	2	0.00080418	0.0450789	HFE:HLA-C

Cell_type_sig	HAY_BONE_I	178	6	0.00021778	0.04518983	EPHA2:NOTC
GWAScatalo	Nasopharyng	13	2	0.00172073	0.0455942	TERT:HLA-C
GWAScatalo	Alzheimer's d	13	2	0.00172073	0.0455942	TOMM40:APC
GO_cc	GOCC_MEMI	1118	14	0.00086105	0.04572653	HFE:HLA-C:H
GWAScatalo	Fish- and plar	180	5	0.00176284	0.0460727	HFE:PGBD1:T
GWAScatalo	Neutrophil co	361	7	0.00177002	0.0460727	GCKR:TERT:H
GWAScatalo	Systemic lupu	361	7	0.00177002	0.0460727	TERT:HSPA1A
GWAScatalo	Albumin-glob	50	3	0.00178608	0.04621875	GCKR:C2orf1
GWAScatalo	Reticulocyte	363	7	0.00182629	0.04698454	GCKR:HFE:N
GWAScatalo	Asthma (chilc	269	6	0.00188691	0.04781931	HLA-C:VWA7
GWAScatalo	Mean arteria	109	4	0.00188845	0.04781931	C2orf16:PDE
GWAScatalo	Non-albumin	51	3	0.00189116	0.04781931	GCKR:TERT:H
Curated_gen	WEST_ADREI	22	3	0.00015465	0.04783004	HLA-DPA1:HI
GWAScatalo	Lung cancer i	110	4	0.0019526	0.04909227	TNXB:NOTCH
GWAScatalo	Alcohol depe	52	3	0.00200002	0.04947235	ADH5:ADH4:
GWAScatalo	Cerebrospina	14	2	0.00200125	0.04947235	APOE:APOC1
GWAScatalo	Cholesterol	14	2	0.00200125	0.04947235	GCKR:ZNF51

link

I4:TM6SF2:SUGP1:MAU2:GATAD2A:TSSK6:NDUFA13:YJEFN3:ATP13A1
.6:ZNF512:NCAN:TM6SF2:SUGP1:GATAD2A:APOE:PNPLA3:SAMM50
http://www.gsea-msigdb.org/gsea/msigdb/human/geneset/chr6p21
H4:HLA-DQA2:HLA-DQB2:HLA-DOA:HLA-DPA1:HLA-DPB1
http://www.gsea-msigdb.org/gsea/msigdb/human/geneset/GOCC_MHC_PROTEIN_COMPLEX
EF19:PRUNE:GCKR:C2orf16:KLHL8:HSD17B13:ADH4:HFE:NOTCH4:HLA-DPB1:SERPINA1:CDH
HLA-C:VWA7:LSM2:HSPA1L:HSPA1A:HSPA1B:DXO:C4A:TNXB:ATF6B:AGPAT1:NOTCH4:HLA-DQA
7B13:TM6SF2:SUGP1:PNPLA3
I4:TM6SF2:SUGP1:MAU2:GATAD2A:TSSK6:NDUFA13:YJEFN3:ATP13A1
http://www.gsea-msigdb.org/gsea/msigdb/human/geneset/GOCC_MHC_CLASS_II_PROTEIN_C
ERPINA1:TM6SF2:SUGP1:GATAD2A:PNPLA3
http://www.gsea-msigdb.org/gsea/msigdb/human/geneset/GOBP_PEPTIDE_ANTIGEN_ASSEMBI
.6:ZNF512:KLHL8:MET:ANGPTL4:NCAN:MAU2:TOMM40:APOE:APOC1
SF2:TSSK6:APOE:PNPLA3
ERPINA1:TM6SF2:PNPLA3
2:APOE:PNPLA3
http://www.gsea-msigdb.org/gsea/msigdb/human/geneset/GOBP_PEPTIDE_ANTIGEN_ASSEMBI
R:TMPRSS11E:ADH1B:HFE:HLA-DQA2:SERPINA1:HAPLN4:TM6SF2:PVRL2:APOE:APOC1
EF19:PRUNE:KLHL8:HSD17B13:HFE:HLA-DPB1:MET:SERPINA1:TM6SF2:APOE:PNPLA3:SAMM50
http://www.gsea-msigdb.org/gsea/msigdb/human/geneset/KEGG_ANTIGEN_PROCESSING_ANI
http://www.gsea-msigdb.org/gsea/msigdb/human/geneset/KEGG_ALLOGRAFT_REJECTION
http://www.gsea-msigdb.org/gsea/msigdb/human/geneset/KEGG_GRAFT_VERSUS_HOST_DISE
http://www.gsea-msigdb.org/gsea/msigdb/human/geneset/KEGG_SYSTEMIC_LUPUS_ERYTHEM
S11E:HFE:HLA-C:SERPINA1:NCAN:SUGP1:TOMM40:APOE:APOC1
http://www.gsea-msigdb.org/gsea/msigdb/human/geneset/GOMF_MHC_CLASS_II_PROTEIN_C
http://www.gsea-msigdb.org/gsea/msigdb/human/geneset/KEGG_TYPE_I_DIABETES_MELLITUS
C:VWA7:LSM2:HSPA1L:HSPA1A:HSPA1B:HLA-DQA2:HLA-DQB2
http://www.gsea-msigdb.org/gsea/msigdb/human/geneset/GOBP_ANTIGEN_PROCESSING_ANI
PGBD1:HLA-C:TNXB:NOTCH4:HLA-DQB2:HLA-DOA:HLA-DPA1:ROS1
http://www.gsea-msigdb.org/gsea/msigdb/human/geneset/KEGG_AUTOIMMUNE_THYROID_DIS
http://www.gsea-msigdb.org/gsea/msigdb/human/geneset/KEGG_ASTHMA
LSM2:HSPA1L:HSPA1A:HSPA1B:DXO:C4A:TNXB:ATF6B:AGPAT1:NOTCH4:HLA-DQA2:HLA-DQB2
QB2:HLA-DPA1:HLA-DPB1
S11E:KLHL8:HLA-DQA2:SERPINA1:HAPLN4:APOE:APOC1:PNPLA3:SAMM50
http://www.gsea-msigdb.org/gsea/msigdb/human/geneset/GOBP_ANTIGEN_PROCESSING_ANI
http://www.gsea-msigdb.org/gsea/msigdb/human/geneset/GOMF_MHC_PROTEIN_COMPLEX_I
http://www.gsea-msigdb.org/gsea/msigdb/human/geneset/GOMF_MHC_CLASS_II_RECEPTOR_
LA-C:NCAN:TOMM40:APOE:APOC1:SAMM50
http://www.gsea-msigdb.org/gsea/msigdb/human/geneset/KEGG_ANTIGEN_PROCESSING_ANI
http://www.gsea-msigdb.org/gsea/msigdb/human/geneset/chr19p13
http://www.gsea-msigdb.org/gsea/msigdb/human/geneset/KEGG_ALLOGRAFT_REJECTION
http://www.gsea-msigdb.org/gsea/msigdb/human/geneset/KEGG_GRAFT_VERSUS_HOST_DISE
http://www.gsea-msigdb.org/gsea/msigdb/human/geneset/KEGG_SYSTEMIC_LUPUS_ERYTHEM
512:NOTCH4:ROS1:NCAN:HAPLN4:TM6SF2:SUGP1:MAU2:GATAD2A:TSSK6:NDUFA13:YJEFN3:A
http://www.gsea-msigdb.org/gsea/msigdb/human/geneset/KEGG_ANTIGEN_PROCESSING_ANI
http://www.gsea-msigdb.org/gsea/msigdb/human/geneset/GOBP_ANTIGEN_PROCESSING_ANI
http://www.gsea-msigdb.org/gsea/msigdb/human/geneset/KEGG_TYPE_I_DIABETES_MELLITUS

http://www.gsea-msigdb.org/gsea/msigdb/human/geneset/KEGG_VIRAL_MYOCARDITIS
http://www.gsea-msigdb.org/gsea/msigdb/human/geneset/GOBP_ANTIGEN_PROCESSING_ANI
H4:C6orf10:HLA-DQA2:HLA-DPA1
http://www.gsea-msigdb.org/gsea/msigdb/human/geneset/KEGG_GRAFT_VERSUS_HOST_DISE/
http://www.gsea-msigdb.org/gsea/msigdb/human/geneset/KEGG_ALLOGRAFT_REJECTION
http://www.gsea-msigdb.org/gsea/msigdb/human/geneset/KEGG_SYSTEMIC_LUPUS_ERYTHEM
)E:APOC1
)E:APOC1
http://www.gsea-msigdb.org/gsea/msigdb/human/geneset/GOCC_LUMENAL_SIDE_OF_ENDOP
.:MAU2:APOE
http://www.gsea-msigdb.org/gsea/msigdb/human/geneset/KEGG_TYPE_I_DIABETES_MELLITUS
http://www.gsea-msigdb.org/gsea/msigdb/human/geneset/KEGG_INTESTINAL_IMMUNE_NETW(
http://www.gsea-msigdb.org/gsea/msigdb/human/geneset/GOBP_ANTIGEN_PROCESSING_ANI
.:MAU2:APOE
RUNE:HSD17B13:SERPINA1:TM6SF2:APOE:PNPLA3:SAMM50
http://www.gsea-msigdb.org/gsea/msigdb/human/geneset/KEGG_CELL_ADHESION_MOLECULI
http://www.gsea-msigdb.org/gsea/msigdb/human/geneset/KEGG_AUTOIMMUNE_THYROID_DIS
http://www.gsea-msigdb.org/gsea/msigdb/human/geneset/KEGG_ASTHMA
http://www.gsea-msigdb.org/gsea/msigdb/human/geneset/GOCC_LUMENAL_SIDE_OF_MEMBF
http://www.gsea-msigdb.org/gsea/msigdb/human/geneset/GOBP_ANTIGEN_PROCESSING_ANI
http://www.gsea-msigdb.org/gsea/msigdb/human/geneset/WP_ALLOGRAFT_REJECTION
S11E:ADH4:HAPLN4:TM6SF2:MAU2:GATAD2A:PVRL2:TOMM40:APOE:APOC1
)E:APOC1
2A:ATP13A1
)QA2:HLA-DPA1:HLA-DPB1
http://www.gsea-msigdb.org/gsea/msigdb/human/geneset/CIITA_TARGET_GENES
:LSM2:HSPA1L:HSPA1A:HSPA1B:DXO:C4A:TNXB:AGPAT1:NOTCH4:HLA-DQA2:HLA-DQB2:HLA-D
http://www.gsea-msigdb.org/gsea/msigdb/human/geneset/KEGG_AUTOIMMUNE_THYROID_DIS
http://www.gsea-msigdb.org/gsea/msigdb/human/geneset/KEGG_ASTHMA
http://www.gsea-msigdb.org/gsea/msigdb/human/geneset/WP_ALLOGRAFT_REJECTION
CKR:TMPRSS11E:ADH1B:HFE:SUGP1:TOMM40:APOE:APOC1
http://www.gsea-msigdb.org/gsea/msigdb/human/geneset/WP_ALLOGRAFT_REJECTION
)PA1:HLA-DPB1
:VWA7:LSM2:HSPA1L:HSPA1A:HSPA1B:DXO:C4A:TNXB:ATF6B:AGPAT1:NOTCH4:HLA-DQA2:HLA
http://www.gsea-msigdb.org/gsea/msigdb/human/geneset/KEGG_VIRAL_MYOCARDITIS
http://www.gsea-msigdb.org/gsea/msigdb/human/geneset/GOBP_B_CELL_MEDIATED_IMMUNI
ADH1B
.6:ZNF512
http://www.gsea-msigdb.org/gsea/msigdb/human/geneset/KEGG_LEISHMANIA_INFECTION
7B13:TERT:HFE:HLA-DOA:SERPINA1:SUGP1:GATAD2A:NDUFA13:YJEFN3:ATP13A1:APOE:PNPLA3
40:APOE:APOC1
NOTCH4:TOMM40:APOE
40:APOE:APOC1
)E:APOC1
)E:APOC1
)E:APOC1
I4:HLA-DQA2:HLA-DPB1
http://www.gsea-msigdb.org/gsea/msigdb/human/geneset/REACTOME_PD_1_SIGNALING

http://www.gsea-msigdb.org/gsea/msigdb/human/geneset/KEGG_INTESTINAL_IMMUNE_NETW
http://www.gsea-msigdb.org/gsea/msigdb/human/geneset/KEGG_VIRAL_MYOCARDITIS
http://www.gsea-msigdb.org/gsea/msigdb/human/geneset/GAURNIER_PSMD4_TARGETS
TL4:PNPLA3
:PNPLA3
LA-DQB2:HLA-DPB1
http://www.gsea-msigdb.org/gsea/msigdb/human/geneset/GOCC_COPII_COATED_ER_TO_GOI
http://www.gsea-msigdb.org/gsea/msigdb/human/geneset/KEGG_CELL_ADHESION_MOLECULI
http://www.gsea-msigdb.org/gsea/msigdb/human/geneset/WP_EBOLA_VIRUS_INFECTION_IN_I
http://www.gsea-msigdb.org/gsea/msigdb/human/geneset/GOBP_IMMUNOGLOBULIN_PRODU
http://www.gsea-msigdb.org/gsea/msigdb/human/geneset/GOBP_LYMPHOCYTE_MEDIATED_IN
:TOMM40:APOE:APOC1
512:ADH1B
OTCH4:MEF2B:NCAN:HAPLN4:TM6SF2:SUGP1:MAU2:GATAD2A:TSSK6:NDUFA13:YJEFN3
.6:KLHL8:HSD17B13:ADH4:HLA-C:MET:ANGPTL4:TM6SF2:SUGP1:APOE:APOC1
TERT:SERPINA1:NCAN:MAU2:GATAD2A:ATP13A1:APOE:APOC1:PNPLA3
:ROBO2:HLA-DQA2:TM6SF2:SUGP1:GATAD2A:TOMM40:APOE:APOC1:PNPLA3:SAMM50
http://www.gsea-msigdb.org/gsea/msigdb/human/geneset/REACTOME_PD_1_SIGNALING
S11E:TM6SF2:TOMM40:APOE
http://www.gsea-msigdb.org/gsea/msigdb/human/geneset/GOBP_ADAPTIVE_IMMUNE_RESPON
http://www.gsea-msigdb.org/gsea/msigdb/human/geneset/WP_EBOLA_VIRUS_INFECTION_IN_I
http://www.gsea-msigdb.org/gsea/msigdb/human/geneset/KEGG_INTESTINAL_IMMUNE_NETW
http://www.gsea-msigdb.org/gsea/msigdb/human/geneset/GOCC_ER_TO_GOLGI_TRANSPORT_
http://www.gsea-msigdb.org/gsea/msigdb/human/geneset/GOMF_ALCOHOL_DEHYDROGENAS
LA-DPA1:HLA-DPB1
)E:APOC1
40:APOE:APOC1
http://www.gsea-msigdb.org/gsea/msigdb/human/geneset/KEGG_CELL_ADHESION_MOLECULI
http://www.gsea-msigdb.org/gsea/msigdb/human/geneset/WP_EBOLA_VIRUS_INFECTION_IN_I
3:HFE:HLA-C:C4A:NOTCH4:SERPINA1:ANGPTL4:GATAD2A:APOE
ADH1B
NOTCH4
EF19:GCKR:C2orf16:ADH1B:SERPINA1:PVRL2

)C1
)E
)E
LA-DPB1
)E
)E
APOC1
)E:APOC1
http://www.gsea-msigdb.org/gsea/msigdb/human/geneset/DURANTE_ADULT_OLFACTORY_NEU
http://www.gsea-msigdb.org/gsea/msigdb/human/geneset/GOMF_PEPTIDE_ANTIGEN_BINDIN
)E:APOC1
KLHL8:ADH5:ADH1B:TERT:HFE:MET:ANGPTL4:APOE:APOC1:PNPLA3
http://www.gsea-msigdb.org/gsea/msigdb/human/geneset/GOCC_CLATHRIN_COATED_ENDO
)E:APOC1

)E:APOC1
http://www.gsea-msigdb.org/gsea/msigdb/human/geneset/REACTOME_GENERATION_OF_SEC
http://www.gsea-msigdb.org/gsea/msigdb/human/geneset/REACTOME_PD_1_SIGNALING
ADH5:ANGPTL4:TOMM40:APOE:APOC1:PNPLA3
http://www.gsea-msigdb.org/gsea/msigdb/human/geneset/GAURNIER_PSMD4_TARGETS
http://www.gsea-msigdb.org/gsea/msigdb/human/geneset/GOMF_ALCOHOL_DEHYDROGENAS
http://www.gsea-msigdb.org/gsea/msigdb/human/geneset/KEGG_LEISHMANIA_INFECTION
CKR:TMPRSS11E:ADH1B:HFE:SUGP1:APOC1
)E:APOC1
:APOC1
)E:APOC1
http://www.gsea-msigdb.org/gsea/msigdb/human/geneset/REACTOME_ETHANOL_OXIDATION
S11E:ADH4:ADH1B:HFE:ANGPTL4:APOE:APOC1:PNPLA3
M50
3
3

)E:APOC1
.6:ZNF512
3:NOTCH4
http://www.gsea-msigdb.org/gsea/msigdb/human/geneset/REACTOME_MHC_CLASS_II_ANTIGE
http://www.gsea-msigdb.org/gsea/msigdb/human/geneset/GOBP_ETHANOL_OXIDATION
)E:APOC1
)E:APOC1
:APOC1
)MM40
C:TNXB:NOTCH4:HLA-DQB2
http://www.gsea-msigdb.org/gsea/msigdb/human/geneset/REACTOME_GENERATION_OF_SEC
3:HFE:HLA-C:SERPINA1:ANGPTL4:GATAD2A:APOE:APOC1
:APOC1
)E:APOC1
http://www.gsea-msigdb.org/gsea/msigdb/human/geneset/chr1q21
MM40:APOE:APOC1
LA-DPB1:SERPINA1
)E
40

A3
LA3
TL4:TOMM40:APOE:APOC1
http://www.gsea-msigdb.org/gsea/msigdb/human/geneset/KEGG_LEISHMANIA_INFECTION
http://www.gsea-msigdb.org/gsea/msigdb/human/geneset/GOCC_CLATHRIN_COATED_ENDO
3:HFE:NOTCH4:SERPINA1:ANGPTL4:GATAD2A:APOE:APOC1
1:CDH8
http://www.gsea-msigdb.org/gsea/msigdb/human/geneset/REACTOME_ETHANOL_OXIDATION
http://www.gsea-msigdb.org/gsea/msigdb/human/geneset/REACTOME_MHC_CLASS_II_ANTIGE
http://www.gsea-msigdb.org/gsea/msigdb/human/geneset/chr4q23
http://www.gsea-msigdb.org/gsea/msigdb/human/geneset/GOBP_LEUKOCYTE_MEDIATED_IMM

http://www.gsea-msigdb.org/gsea/msigdb/human/geneset/REACTOME_GENERATION_OF_SEC
http://www.gsea-msigdb.org/gsea/msigdb/human/geneset/REACTOME_ETHANOL_OXIDATION
http://www.gsea-msigdb.org/gsea/msigdb/human/geneset/REACTOME_MHC_CLASS_II_ANTIGE
EF19:GCKR:MET
DE:APOC1
HOD3:APOE:APOC1
NL3
32:KIAA1328
.:PVRL2
http://www.gsea-msigdb.org/gsea/msigdb/human/geneset/GOCC_COATED_VESICLE
.:PVRL2
http://www.gsea-msigdb.org/gsea/msigdb/human/geneset/REACTOME_INTERFERON_GAMMA_
3:SERPINA1:CDH8
7B13:HLA-C:LSM2:HLA-DPB1
http://www.gsea-msigdb.org/gsea/msigdb/human/geneset/GOBP_IMMUNOGLOBULIN_PRODU
http://www.gsea-msigdb.org/gsea/msigdb/human/geneset/MODULE_143
http://www.gsea-msigdb.org/gsea/msigdb/human/geneset/MODULE_23
NA1:PNPLA3
DE:APOC1

DH8
3
DE

B1
ADH1B:SERPINA1
DE:APOC1
DE:APOC1
http://www.gsea-msigdb.org/gsea/msigdb/human/geneset/KEGG_FATTY_ACID_METABOLISM
http://www.gsea-msigdb.org/gsea/msigdb/human/geneset/KEGG_TYROSINE_METABOLISM
DE:APOC1
APOC1
http://www.gsea-msigdb.org/gsea/msigdb/human/geneset/NFY_Q6_01
http://www.gsea-msigdb.org/gsea/msigdb/human/geneset/GOCC_BLOOD_MICROPARTICLE
M50
CDH8
DE:APOC1
DE:APOC1
3:SAMM50
XB:ATF6B:AGPAT1:NOTCH4:HLA-DOA
http://www.gsea-msigdb.org/gsea/msigdb/human/geneset/TARTE_PLASMA_CELL_VS_PLASMABI
http://www.gsea-msigdb.org/gsea/msigdb/human/geneset/REACTOME_INTERFERON_GAMMA_
http://www.gsea-msigdb.org/gsea/msigdb/human/geneset/REACTOME_INTERFERON_GAMMA_
http://www.gsea-msigdb.org/gsea/msigdb/human/geneset/GOCC_ENDOCYTIC_VESICLE_MEM
http://www.gsea-msigdb.org/gsea/msigdb/human/geneset/GOCC_VACUOLAR_MEMBRANE
http://www.gsea-msigdb.org/gsea/msigdb/human/geneset/GOCC_COATED_VESICLE_MEMBRA
LA-DQB2
http://www.gsea-msigdb.org/gsea/msigdb/human/geneset/GOCC_PLASMA_MEMBRANE_PROT

http://www.gsea-msigdb.org/gsea/msigdb/human/geneset/GOCC_CLATHRIN_COATED_VESICL
http://www.gsea-msigdb.org/gsea/msigdb/human/geneset/GOBP_ADAPTIVE_IMMUNE_RESPON
)E:APOC1
NGPTL4:MAU2:GATAD2A:APOE:APOC1:PNPLA3
.6:ZNF512
ADH1B
NA1:APOE:APOC1
http://www.gsea-msigdb.org/gsea/msigdb/human/geneset/MODULE_143
http://www.gsea-msigdb.org/gsea/msigdb/human/geneset/MODULE_23
S11E:ADH4:SERPINA1:TM6SF2:ATP13A1:APOC1
L
40

http://www.gsea-msigdb.org/gsea/msigdb/human/geneset/GOMF_ANTIGEN_BINDING
HLA-C:VWA7:TNXB:TM6SF2:PNPLA3
http://www.gsea-msigdb.org/gsea/msigdb/human/geneset/MODULE_84
LA-DPA1:HLA-DPB1
http://www.gsea-msigdb.org/gsea/msigdb/human/geneset/GOCC_CLATHRIN_COATED_VESICL
GBD1:TNXB:NCAN:HAPLN4:SUGP1:MAU2:GATAD2A:TSSK6:NDUFA13:YJEFN3:ATP13A1:TOMM40
:MAP4K2:MEN1:PVRL2:PNPLA3
A-DPB1
3
LA-DPB1
)E:APOC1
http://www.gsea-msigdb.org/gsea/msigdb/human/geneset/FAN_EMBRYONIC_CTX_BRAIN_B_C
HFE:NOTCH4:MET:TM6SF2:PNPLA3
)E:APOC1
)E:APOC1
http://www.gsea-msigdb.org/gsea/msigdb/human/geneset/AIZARANI_LIVER_C17_HEPATOCYTE
http://www.gsea-msigdb.org/gsea/msigdb/human/geneset/REACTOME_FORMATION_OF_THE_I
3
.6

http://www.gsea-msigdb.org/gsea/msigdb/human/geneset/KEGG_RETINOL_METABOLISM
http://www.gsea-msigdb.org/gsea/msigdb/human/geneset/REACTOME_COSTIMULATION_BY_TI
)E:APOC1
http://www.gsea-msigdb.org/gsea/msigdb/human/geneset/GOCC_CELL_SURFACE
40:APOE:APOC1
http://www.gsea-msigdb.org/gsea/msigdb/human/geneset/GOBP_ETHANOL_METABOLIC_PRO
http://www.gsea-msigdb.org/gsea/msigdb/human/geneset/KEGG_GLYCOLYSIS_GLUCONEOGE
http://www.gsea-msigdb.org/gsea/msigdb/human/geneset/KEGG_AXON_GUIDANCE
.6
PGBD1:SERPINA1:NCAN:HAPLN4:TM6SF2:SUGP1:MAU2:GATAD2A:TSSK6:NDUFA13:YJEFN3
http://www.gsea-msigdb.org/gsea/msigdb/human/geneset/KEGG_METABOLISM_OF_XENOBIOT
http://www.gsea-msigdb.org/gsea/msigdb/human/geneset/KEGG_DRUG_METABOLISM_CYTOC
)E:APOC1
2:PNPLA3
http://www.gsea-msigdb.org/gsea/msigdb/human/geneset/GOCC_MHC_CLASS_I_PROTEIN_CO

http://www.gsea-msigdb.org/gsea/msigdb/human/geneset/HAY_BONE_MARROW_DENDRITIC_

)E
http://www.gsea-msigdb.org/gsea/msigdb/human/geneset/GOCC_MEMBRANE_PROTEIN_COM
TOMM40:APOE:APOC1
ILA-C:TNXB:HLA-DPB1:ATP13A1:PNPLA3
:HSPA1B:TNXB:NOTCH4:HLA-DQA2:HLA-DOA:HLA-DPA1
.6:HLA-C
OTCH4:SERPINA1:ANGPTL4:GATAD2A:APOE
:LSM2:HSPA1L:HSPA1A:HSPA1B:HLA-DPA1
1A:HFE:MEF2B
ILA-C
http://www.gsea-msigdb.org/gsea/msigdb/human/geneset/WEST_ADRENOCORTICAL_CARCIN
I4:HLA-DQA2:HLA-DPA1
ADH1B

.2

8:TM6SF2:APOE:APOC1:PNPLA3
.2:HLA-DQB2:HLA-DMA:HLA-DOA:HLA-DPA1:HLA-DPB1

OMPLEX

.Y_WITH_MHC_CLASS_II_PROTEIN_COMPLEX

.Y_WITH_MHC_PROTEIN_COMPLEX

)_PRESENTATION

ASE
ATOSUS

OMPLEX_BINDING

)_PRESENTATION_OF_PEPTIDE_ANTIGEN

SEASE

)_PRESENTATION_OF_EXOGENOUS_PEPTIDE_ANTIGEN_VIA_MHC_CLASS_II
BINDING
.ACTIVITY

)_PRESENTATION

ASE
ATOSUS
TP13A1
)_PRESENTATION
)_PRESENTATION_OF_PEPTIDE_OR_POLYSACCHARIDE_ANTIGEN_VIA_MHC_CLASS_II

D_PRESENTATION_OF_EXOGENOUS_PEPTIDE_ANTIGEN

ASE

ATOSUS

PLASMIC_RETICULUM_MEMBRANE

ORK_FOR_IGA_PRODUCTION
D_PRESENTATION

ES_CAMS
SEASE

RANE
D_PRESENTATION_OF_EXOGENOUS_ANTIGEN

MA:HLA-DOA:HLA-DPA1:HLA-DPB1
SEASE

-DQB2

TY

;

ORK_FOR_IGA_PRODUCTION

LGI_TRANSPORT_VESICLE
ES_CAMS
HOST
CTION_INVOLVED_IN_IMMUNOGLOBULIN_MEDIATED_IMMUNE_RESPONSE
MMUNITY

NSE_BASED_ON_SOMATIC_RECOMBINATION_OF_IMMUNE_RECEPTORS_BUILT_FROM_IMMUN
HOST
ORK_FOR_IGA_PRODUCTION
_VESICLE_MEMBRANE
SE_ACTIVITY_ZINC_DEPENDENT

ES_CAMS
HOST

UROEPITHELIUM_MACROPHAGES
G

CYTIC_VESICLE_MEMBRANE

OND_MESSENGER_MOLECULES

SE_NAD_P_PLUS_ACTIVITY

EN_PRESENTATION

OND_MESSENGER_MOLECULES

CYTIC_VESICLE

EN_PRESENTATION

MUNITY

OND_MESSENGER_MOLECULES

EN_PRESENTATION

_SIGNALING

CTION

LAST_UP
_SIGNALING
_SIGNALING
BRANE

ANE

EIN_COMPLEX

LE_MEMBRANE
NSE

LE
D:APOE:APOC1

CELL

ES_3
BETA_CATENIN_TCF_TRANSACTIVATING_COMPLEX

HE_CD28_FAMILY

CESS
ENESIS

TICS_BY_CYTOCHROME_P450
CHROME_P450

OMPLEX

_CELL

1PLEX

OMA_VS_ADENOMA_DN

OGLOBULIN_SUPERFAMILY_DOMAINS

Chr:start-end	Gene	GWAS SNP	P(GWAS)	eQTL SNP	P(eQTL)
chr1:15829591-15829592	EPHA2	rs6656611	1.08E-12	rs7538216	2.27E-08
chr1:15910666-15910667	CPLANE2	rs6656611	1.08E-12	rs6693819	7.25E-15
chr19:19162581-19162582	TM6SF2	rs10401969	7.82E-48	rs4808200	0.00011812
chr19:19928696-19928697	ZNF682	rs17216588	2.02E-40	rs6511071	1.25E-12
chr1:16124337-16156069	EPHA2	rs6656611	1.10E-12	rs3820609	1.87E-05
chr1:16231688-16237164	C1orf89	rs6656611	1.10E-12	rs6693819	1.75E-06
chr14:24290600-24299833	DHRS1	rs2295308	0.00193521	rs10134537	5.12E-05
chr1:15829591-15829592	EPHA2	rs11588341	6.97E-13	rs7538216	2.27E-08
chr1:15910666-15910667	CPLANE2	rs11588341	6.97E-13	rs6693819	7.25E-15
chr19:19162581-19162582	TM6SF2	rs10401969	2.47E-33	rs4808200	0.00011812
chr1:15829591-15829592	EPHA2	rs6656611	1.63E-13	rs7538216	2.27E-08
chr1:15910666-15910667	CPLANE2	rs6656611	1.63E-13	rs6693819	7.25E-15
chr19:19162581-19162582	TM6SF2	rs10401969	1.42E-46	rs4808200	0.00011812
chr19:19508106-19508107	PBX4	rs10401969	1.42E-46	rs1476459	6.33E-05
chr19:19928696-19928697	ZNF682	rs17216588	2.65E-40	rs6511071	1.25E-12
